# Biological and therapeutic implications of sex hormone-related gene clustering in testicular cancer

**DOI:** 10.1186/s12610-025-00254-5

**Published:** 2025-02-26

**Authors:** Péter Törzsök, Frédéric R. Santer, Yannic Kunz, Nils C. H. van Creij, Piotr Tymoszuk, Gerald Klinglmair, Zoran Culig, Renate Pichler

**Affiliations:** 1 Faculty of Health and Sport Sciences, Széchenyi István University, Győr, Hungary; 2 Division of Experimental Urology, Department of Urology, Medical University of Innsbruck, Innsbruck, Austria; 3 Department of Urology, Medical University of Innsbruck, Comprehensive Cancer Center Innsbruck (CCCI), Anichstraße 35, Innsbruck, 6020 Austria; 4 Data Analytics As a Service Tirol, Innsbruck, Austria

**Keywords:** Testicular cancer, Sex hormones, Molecular cluster, Drug response, Cancer du Testicule, Hormones sexuelles, Groupe moléculaire, Réponse aux Médicaments

## Abstract

**Background:**

Gonadotropin dysregulation seems to play a potential role in the carcinogenesis of testicular germ cell tumor (TGCT). The aim of this study was to explore the expression of specific genes related to sex hormone regulation, synthesis, and metabolism in TGCT and to define specific hormonal clusters. Two publicly available databases were used for this analysis (TCGA and GSE99420). By means of hard-threshold regularized KMEANS clustering, we assigned TGCT samples into four clusters defined in respect to different expression of the sex hormone-related genes. We analysed clinical data, protein and gene expression, signaling regarding hormonal clusters. Based on whole-transcriptome gene expression, prediction of anti-cancer drug response was made by RIDGE models.

**Results:**

Cluster #1 (12–16%) consisted primarily of non-seminomatous germ cell tumor (NSGCT), characterized by high expression of *PRL*, *GNRH1*, *HSD17B2* and *SRD5A1*. Cluster #2 (42–50%) included predominantly seminomas with high expression of *SRD5A3,* being highly infiltrated by T and B cells. Cluster #3 (8.3–18%) comprised of NSGCT with high expression of *CGA*, *CYP19A1*, *HSD17B12*, *HSD17B1*, *SHBG.* Cluster #4 (23–30%), which consisted primarily of NSGCT with a small fraction of seminomas, was outlined by increased expression of *STAR*, *POMC*, *CYP11A1*, *CYP17A1*, *HSD3B2* and *HSD17B3*. Elevated fibroblast levels and increased extracellular matrix- and growth factor signaling-related gene signature scores were described in cluster #1 and #3. In the combined model of progression-free survival, S2/S3 tumor marker status, hormonal cluster #1 or #3 and teratoma histology, were independently associated with 25–30% increase of progression risk. Based on the increased receptor tyrosine kinase and growth factor signaling, cluster #1, #3 and #4 were predicted to be sensitive to tyrosine kinase inhibitors, FGFR inhibitors or EGFR/ERBB inhibitors. Cluster #2 and #4 were responsive to compounds interfering with DNA synthesis, cytoskeleton, cell cycle and epigenetics. Response to apoptosis modulators was predicted only for cluster #2.

**Conclusions:**

Hormonal cluster #1 or #3 is an independent prognostic factor regarding poor progression-free survival. Hormonal cluster assignment also affects the predicted drug response with cluster-dependent susceptibility to specific novel therapeutic compounds.

**Supplementary Information:**

The online version contains supplementary material available at 10.1186/s12610-025-00254-5.

## Résumé

**Contexte:**

La dysrégulation des gonadotrophines semble jouer un rôle potentiel dans la cancérogenèse des tumeurs germinales testiculaires (TGCT). Le but de cette étude était d’explorer l’expression de gènes spécifiques liés à la régulation, à la synthèse et au métabolisme des hormones sexuelles dans les TGCT et de définir des groupes hormonaux spécifiques. Deux bases de données, accessibles au public, ont été utilisées pour cette analyse (TCGA et GSE99420). Au moyen d’un regroupement KMEANS régularisé à seuil dur, nous avons réaffecté des échantillons TGCT en quatre groupes définis en fonction de l’expression différente des gènes liés aux hormones sexuelles. Nous avons analysé les données cliniques, l’expression des protéines et des gènes, et la signalisation concernant ces groupes hormonaux. Sur la base de l’expression génique du transcriptome entier, la prédiction de la réponse aux médicaments anticancéreux a été réalisée par des modèles RIDGE.

**Résultats:**

Le groupe #1 (12-16 %) était principalement constitué de tumeurs germinales non séminomateuses (NSGCT), caractérisées par une expression élevée de *PRL*, *GNRH1*, *HSD17B2* et *SRD5A1*. Le groupe #2 (42-50 %) comprenait principalement des séminomes, avec une forte expression de *SRD5A3*, fortement infiltrés par des lymphocytes T et B. Le groupe #3 (8,3-18 %) comprenait des NSGCT avec une expression élevée de *CGA*, *CYP19A1*, *HSD17B12*, *HSD17B1*, *SHBG*. Le groupe #4 (23-30 %), qui consistait principalement en NSGCT avec une petite fraction de séminomes, a été mis en évidence par une expression accrue de *STAR*, *POMC*, *CYP11A1*, *CYP17A1*, *HSD3B2* et *HSD17B3*. Des niveaux élevés de fibroblastes, et des scores de signature génique liés à la matrice extracellulaire et aux facteurs de croissance, ont été décrits dans les groupes #1 et #3. Dans le modèle combiné de survie sans progression, le statut du marqueur tumoral S2/S3, le groupe hormonal #1 ou #3, et l’histologie du tératome étaient indépendamment associés à une augmentation de 25 à 30 % du risque de progression. Sur la base de l’augmentation de la signalisation du récepteur tyrosine kinase et du facteur de croissance, les groupes #1, #3 et #4 ont été prédits comme étant sensibles aux inhibiteurs de la tyrosine kinase, aux inhibiteurs du FGFR ou aux inhibiteurs de l’EGFR/ERBB. Les groupes #2 et #4 étaient sensibles aux composés interférant avec la synthèse de l’ADN, le cytosquelette, le cycle cellulaire et l’épigénétique. La réponse aux modulateurs de l’apoptose n’a été prédite que pour le groupe #2.

**Conclusions:**

Les groupes hormonaux #1 ou #3 sont des facteurs pronostiques indépendants d’une faible survie sans progression. L’attribution hormonale des groupes affecte également la réponse médicamenteuse prédite avec une susceptibilité groupe-dépendante à de nouveaux composés thérapeutiques spécifiques.

**Graphical Abstract:**

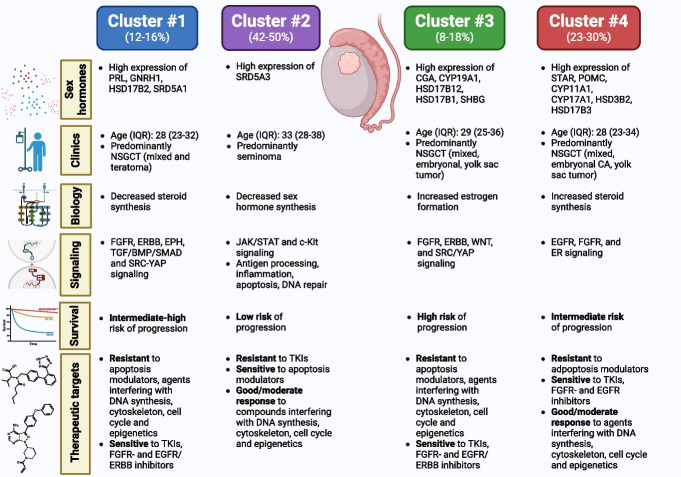

## Background

Testicular germ cell tumor (TGCT) is the most common cancer entity amongst men aged 20–40 years, accounting for one-fourth of tumors in this age range, with a rising incidence [1, 2]. Seminomatous germ cell tumors (SGCT) and non-seminomatous germ cell tumors (NSGCT) are the two subtypes distinguished by their histological and clinical features [1]. Non-seminomatous pathology is predicted by elevated preorchiectomy estradiol, while elevated preorchiectomy LH and FSH are linked with increasing tumor diameter [3]. About 60% of testicular tumors are producing one or more tumor marker [4]. However, the classical histopathological features continue to be essential in predicting relapse in testicular germ cell tumors [5]. Seminoma typically exhibits a less aggressive course with a high level of immune infiltration, whereas NSGCT tends to occur in younger individuals and have higher mortality rates [6, 7].

The hypothesis that gonadotropin dysregulation is a potential cause of TGCT has already been proposed [8, 9]. The hypothalamic-hypophyseal-gonadal axis is altered in patients with TGCT, particularly NSGCTs, already before primary treatment [10]. The interaction of maternal steroid hormones during early pregnancy can be a key factor in the development of testicular cancer in offspring [11]. The precursor stage known as germ cell neoplasia in situ (GCNIS) is the intermediate step between foetal gonocytes and TGCT. During puberty, endocrine mechanisms, such as the influence of steroid hormones and/or gonadotrophins, are believed to regulate the later progression of GCNIS cells to invasive seminomatous or non-seminomatous tumors [12]. Lack of KIT mutations were observed in all NSGCTs, including mixed TGCTs with seminoma components, indicating that only seminomas without KIT Proto-Oncogene mutations can acquire nonseminomatous histology [7].

Some of the genes involved in the hypothalamic-hypophyseal-gonadal axis, such as the key gonadotropin-releasing hormones (GNRH1, GNRH2), pituitary gonadotropins (PRL, CGA, LHB and FSHB) as well as gene involved in gonadal testosterone (CYP11A1, CYP17A1, HSD17B3, HSD3B1, HSD3B2) and estradiol synthesis (CYP19A1, HSD17B1) and the gene coding for the steroid hormone transport protein (SHBG) have been investigated previously [12–24].

As we previously reported, pre-surgery sex hormonal levels are differentially disturbed in TGCT [25]. Consecutively, our aim was to explore cancer tissue expression and co-regulation patterns of genes related to sex hormone regulation, synthesis, and metabolism from the The Cancer Genome Atlas (TCGA), GSE99420 and GSE3218 are databases [26–28]. Furthermore, we aimed to classify TGCT samples in respect to varying expression of those sex hormone-related genes by means of semi-supervised clustering, and characterized clinical, prognostic, biological, and pharmacological background of the resulting hormonal clusters.

## Methods

Bioinformatic and statistics analyses and visualizations were done with R version 4.2.3. For detailed information on methods and software, please refer to Supplementary Material.

### Data sources

Publicly available TCGA (source: cBioportal, total cancers: *n *= 149, seminoma: 43%, NSGCT: 57% of samples) [29] and GSE99420 (source: GEO, total cancers: *n* = 60, seminoma: 50%, NSGCT: 50%) or samples) transcriptomic study collectives were analyzed. For the TCGA cohort detailed information of demographics, clinical features, histology and tumor pathology, and survival were provided along with RNA sequencing gene expression quantification, protein quantification results by reverse phase protein array, somatic mutations, gene amplifications and deletions [29]. For the GSE99420 cohort, information of cancer histology (seminoma/NSGCT) and presence of relapse was provided together with transcriptome measurements with an Illumina microarray platform. Characteristic of the cohorts is provided in Table 1. Of note, because of strikingly different distribution of histological subtypes, and in particular significantly lower frequency of seminomas, we were not able to fully reproduce results of the current analysis in another transcriptomic data set, GSE3218 [27]. The analysis results for the GSE3218 collective are available in the R analysis pipeline of the project and may be explored by an interesting reader.

**Table 1:** Characteristic of the investigated cohorts of testicular cancer patients

Variable^a^	TCGA	GSE99420	Significance^b^	Effect size^b^
Age	31 [IQR: 26—37] range: 14—67 complete: *n* = 133			
Race/Ethnicity	Asian: 3.1% (4) Black or African American: 4.7% (6) White: 92% (118) complete: *n* = 128			
Tumor stage	I: 84% (102) II: 9% (11) III: 7.4% (9) complete: *n* = 122			
Metastasis stage	M0: 97% (114) M1: 3.4% (4) complete: *n* = 118			
Node stage	N0: 79% (46) N1: 17% (10) N2: 3.4% (2) complete: *n* = 58			
IGCCCG	good: 74% (32) intermediate: 21% (9) poor: 4.7% (2) complete: *n* = 43			
Histology	seminoma: 43% (62) NSGCT: 57% (82) complete: *n* = 144	seminoma: 50% (30) NSGCT: 50% (30) complete: *n* = 60	ns (*p* = 0.45)	V = 0.064
Histology, ICD-O	seminoma: 49% (65) germinal mixed histology: 20% (27) benign teratoma: 3.8% (5) embryonal carcinoma: 20% (27) malignant teratoma: 2.3% (3) teratocarcinoma: 1.5% (2) yolk sac cancer: 3% (4) complete: *n* = 133			
Marker status	S0: 36% (43) S1: 31% (37) S2: 29% (34) S3: 4.2% (5) complete: *n* = 119			
Neoadjuvant therapy	no: 100% (133) complete: *n* = 133			
Radiation	16% (21) complete: *n* = 130			
Follow-up, months	1300 [IQR: 680—2700] range: 3—7400 complete: *n* = 133			
Death	3% (4) complete: *n* = 133			
Relapse	25% (33) complete: *n* = 133	50% (30) complete: *n* = 60	*p* = 0.002	V = 0.25
Progression	26% (34) complete: *n* = 133			

^a^*IGCCCG* International Germ Cell Cancer Collaborative Group risk strata, *ICD-0* International classification of diseases for oncology, histological subtype

^b^Numeric variables: Kruskal–Wallis test with η^2^ effect size statistic. Categorical variables: χ^2^ test with Cramer‘s V effect size statistic. *P* values corrected for multiple testing with the false discovery rate method

Numeric variables are presented as medians with interquartile ranges and ranges. Qualitative variables are shown as percentages and counts of the categories within the complete observation set

Normalized, $lo{g}_{2}$-transformed expression data for genes and proteins provided by authors were used in analyses. Sex hormone-related genes used for characteristic and classification of testicular cancers consisted of genes coding for pituitary gonadotropins, and genes involved in steroid biosynthesis, inter-conversion, transport and catabolism of estrogens and androgens. Those genes were extracted from ‘Metabolism of steroidhormones’, ‘Estrogen biosynthesis’, and ‘Androgen biosynthesis’ Reactome pathways and are listed in Table 2. $lo{g}_{2}$ mRNA levels of the sex-hormone genes were adjusted for the cohort/batch effect with the ComBat algorithm [30] prior to differential gene expression, co-regulation, and clustering analyses.

**Table 2:** Sex hormone-related genes of interest

Gene classification	Gene symbol	Entrez ID
pituitary	*GNRH1*	2796
	*GNRH2*	2797
	*PRL*	5617
	*CGA*	1081
	*LHB*	3972
	*POMC*	5443
steroid	*STAR*	6770
	*STARD3*	10,948
	*STARD3NL*	83,930
	*STARD4*	134,429
	*TSPO*	706
	*TSPOAP1*	9256
	*CYP11A1*	1583
	*CYP17A1*	1586
	*FDX1*	2230
	*FDX2*	112,812
	*FDXR*	2232
	*HSD3B1*	3283
	*HSD3B2*	3284
	*SERPINA6*	866
adrenal	*CYP21A2*	1589
	*HSD11B1*	3290
	*HSD11B2*	3291
gonadal	*HSD17B1*	3292
	*HSD17B2*	3294
	*HSD17B3*	3293
	*HSD17B11*	51,170
	*HSD17B12*	51,144
	*HSD17B14*	51,171
	*CYP19A1*	1588
	*SRD5A1*	6715
	*SRD5A2*	6716
	*SRD5A3*	79,644
	*SHBG*	6462

Sex hormone-related genes used for characteristic and classification of testicular cancers consisted of genes coding for pituitary gonadotropins, and genes involved in steroid biosynthesis, inter-conversion, transport and catabolism of estrogens and androgens are listed in Table 2. Those genes were extracted from ‘Metabolism of steroid hormones’, ‘Estrogen biosynthesis’, and ‘Androgen biosynthesis’ Reactome pathways

Based on whole-transcriptome gene expression, predictions of anti-cancer drug response to 305 and 214 anti-cancer compounds for single cancer samples of the TCGA and GSE99420 cohorts, in form of log IC50 and AUC (are under the dose–response curve) were made by RIDGE linear regression models [31–33] provided by R package *htGLMNET* and trained with results of in vitro drug screening experiments GDSC [34] and CTRP2 [35]. Estimates of non-malignant cell contents in cancer specimens were computed by *QuanTIseq*, *xCell*, and *MCP Counter* immunedeconvolution algorithms [36–38]. Single sample gene set enrichment analysis scores (ssGSEA scores) were computed for Reactome pathway gene signatures (source: MSig database, version 7.5.1) with the *GSVA* algorithm [39].

Cancer and testis antigen genes, estrogen- and androgen-responsive genes were extracted from CTpedia database and literature reports [40–43]. Annotation and classification of matrisome genes, i.e. features coding for proteins of extracellular matrix (ECM), was done with *MatrisomeAnalyzeR* R package [44, 45]. Other genes of interest were manually searched in the NCBI Gene database.

### Statistical analysis

Descriptive statistics presented for numeric variables are medians, interquartile ranges, ranges and numbers of complete observations. For qualitative variables, percentages and counts of observations in the categories are presented. Statistical significance for differences in numeric variables between strata were investigated by two-tailed T tests, Mann–Whitney tests, Kruskal–Wallis tests, and one-way ANOVA with Cohen’s d, r and ${\eta }^{2}$ effect size statistics. Statistical significance for differences in distribution of categories of categorical variables between strata was determined by ${\chi }^{2}$ test with Cramer’s V effect size statistics. P values were adjusted for multiple comparisons with the false discovery rate (FDR) method [46] separately for the analysis step and cohort. If not indicated otherwise, effects with FDR-adjusted *p* values < 0.05 were considered significant.

## Results

### Sex hormone-related gene expression according to histology

**Fig. 1:**
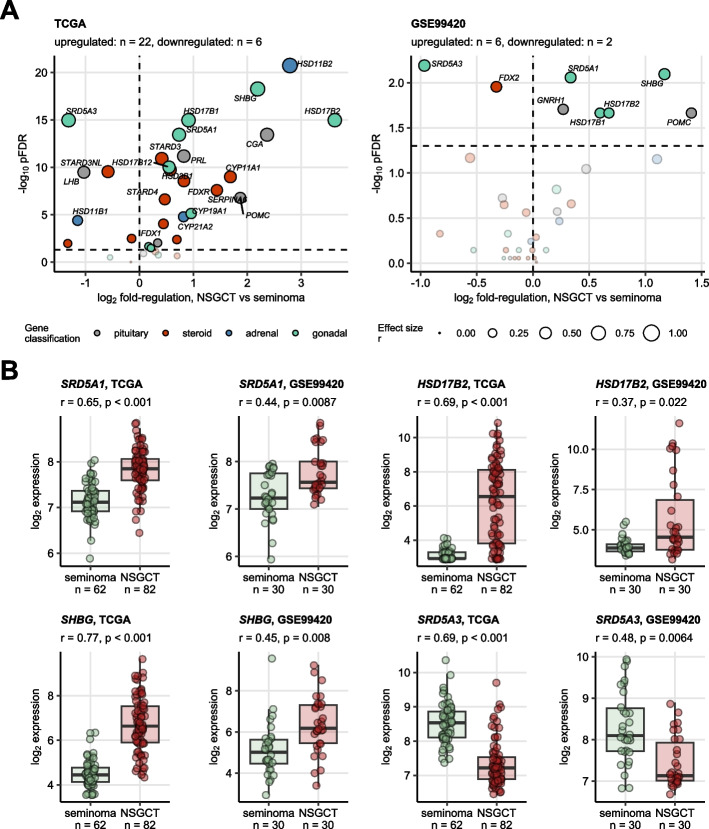
Differential expression of hormone-related genes in NSGCT and seminoma. ${\text{log}}_{2}$-transformed expression of 34 sex hormone-related genes in the cancer tissue was compared between testicular nonseminomatous germ cell tumors (NSGCT) and seminomas in the TCGA and GSE99420 cohorts by Mann–Whitney test with r effect size statistic. *P* values were corrected for multiple testing with the false discovery rate (FDR) method. **A** Differences in median ${\text{log}}_{2}$ expression between NSGCT and seminoma and FDR-corrected *p* values are presented in volcano plots. Each point represents a single gene. Point sizes represent effect size *r* value. Point color codes for gene classification. Significant genes are highlighted and labelled with their symbols. The dashed horizontal lines represent the pFDR = 0.05 significance cutoffs. Numbers of significantly up- and downregulated genes in NSGCT as compared with seminoma are displayed in the plot captions. **B** Expression of selected significant genes shared by both investigated cohorts. Median ${\text{log}}_{2}$ expression values with interquartile ranges are presented as boxes with whiskers spanning over 150% of the interquartile ranges. Cancer samples are visualized as points. Effect sizes and p values are displayed in the plot captions. Numbers of samples are indicated in the X axes

In a comparison of expression levels of the sex hormone-related genes between NSGCT and seminoma, the *POMC* gene coding for an essential regulator of steroid biosynthesis, and genes involved in metabolism (*HSD17B1*, *HSD17B2*, *SRD5A1*) and transport of sex hormones (*SHBG*) were significantly upregulated in NSGCT in both the TCGA and GSE99420 cohorts. The *SRD5A3* gene coding for a testosterone—dihydro-testosterone (DHT) converting enzyme was the sole transcript upregulated in seminoma in both collectives (Fig. 1). In the TCGA cohort, seminoma was characterized by the highest expression of *LHB* (luteinizing hormone subunit beta) and *SRD5A3*. High mRNA levels of genes coding for pituitary gonadotropin subunits *CGA* and *GNRH2* hallmarked mixed germ cell tumors and embryonal carcinomas. Expression of genes involved in general steroid biosynthesis (*STAR*, *POMC*, *CYP11A1*, *FDX1*) and the aromatase-coding gene *CYP19A1* was the highest in embryonal carcinomas. Pituitary hormone-coding *PRL* and *GNRH1* peaked in teratoma, while *SHBG* mRNA levels were the highest in yolk sac tumors (Supplementary Figure S1).

A co-expression analysis revealed a highly co-regulated set of hub genes involved in general steroidogenesis (*CYP11A1*, *CYP17A1*, *STAR*, *HSD3B1*, *HSD3B2*) and synthesis of male and female hormones (*CYP19A1*, *HSD17B12*, *HSD17B3*, *SRD5A1*, *SRD5A2*) as well as their transport and catabolism (*SHBG*, HSD17B2) (Supplementary Figure S2). Those findings combined with the results of differential gene expression analysis for testicular cancer histologies suggest that testicular cancers are equipped with the complete synthetic and regulatory machinery of steroid sex hormone metabolism.

### Clustering regarding sex hormone-relates genes

By means of hard-threshold regularized KMEANS clustering [47], we assigned cancer samples of the training TCGA cohort into four clusters defined in respect to differing expression of the sex hormone-related genes (Table 2, Supplementary Figure S3A). Subsequently, we predicted the hormonal cluster assignment of cancer samples of the GSE99420 cohort with a Random Forest classifier [48] fed with cancer tissue levels of the cluster-defining transcripts. There were no significant differences in distribution of sizes of the hormonal clusters between the training and test collective (Supplementary Figure S3B). An analysis of pairwise distances between observations of each of the TCGA and GSE99420 cohort and cross-distances between the TCGA and GSE99420 samples indicated sufficient separability of the clusters in each of the cohorts and similarity of the corresponding clusters of the training and test cohort clusters (Supplementary Figure S3C).

**Fig. 2:**
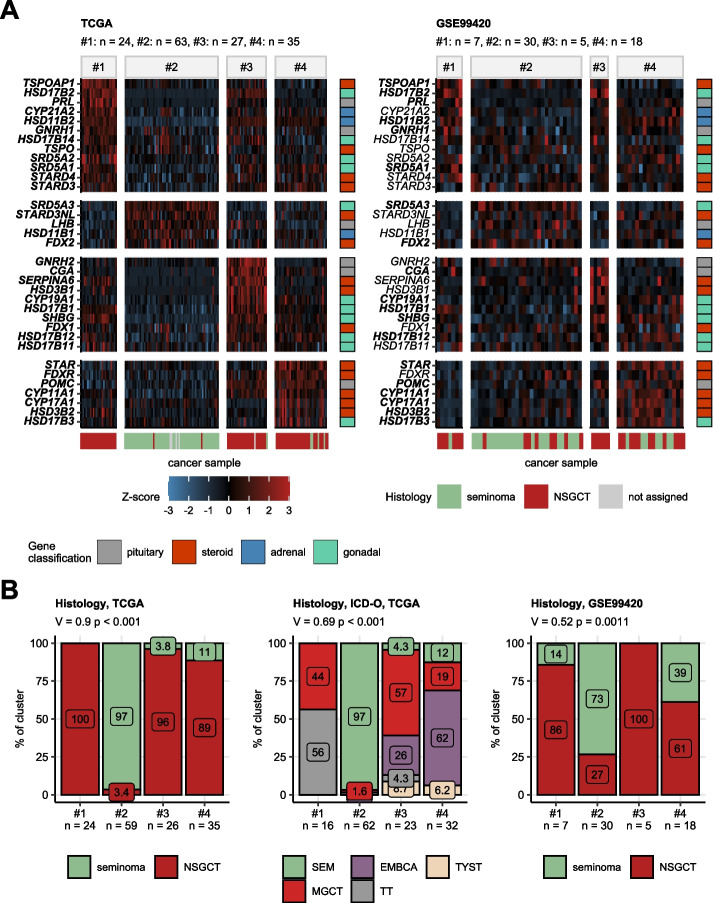
Hormonal clusters of testicular cancers. Testicular cancer samples in the TCGA training cohort were assigned to the hormonal clusters in respect to ${\text{log}}_{2}$ expression values of 34 sex hormone-related genes by hard-threshold regularized KMEANS unsupervised clustering. The hormonal cluster assignment was predicted for cancer samples in the GSE99420 training collective by a Random Forest classifier fed with ${\text{log}}_{2}$-transformed expression levels of the cluster-defining genes. **A** Normalized ${\text{log}}_{2}$-transformed expression values (Z-scores) of the cluster defining genes are presented in a heat map. Genes are arranged by their peak expression in the hormonal clusters of the TCGA cohort. Functional gene classification is color coded in the vertical rug plots. Sample histology (seminoma or nonseminomatous germ cell tumors [NSGCT]) is color coded in the horizontal rug plots. Statistical significance for differences in expression between the hormonal clusters was assessed by Kruskal–Wallis test with ${\upeta }^{2}$ effect size statistic. P values were corrected for multiple testing with the false discovery rate method. Significant differences are highlighted by bold font of the gene symbol in the Y axes of the heat maps. Numbers of samples in the clusters are displayed in the plot captions. **B** Differences in distribution of major cancer histologies and histological subtypes (ICD-O: international classification of diseases for oncology) between the hormonal clusters were investigated by ${\upchi }^{2}$ test with Cramer’s V effect size statistic. *P* values were corrected for multiple testing with the false discovery rate method. Percentages of histologies within the clusters are presented in stack plots. Effect sizes and p values are displayed in the plot captions. Numbers of samples in the clusters are indicated in the X axes

Hormonal cluster #1 consisted primarily of NSGCT malignancies (12 to 16% of samples, mixed and teratoma subtypes) and were characterized by high expression of the pituitary hormones *PRL* and *GNRH1*, *HSD17B2* coding for sex hormone-deactivating enzyme, and *SRD5A1* coding for a testosterone—DHT converting enzyme. The largest hormonal cluster #2 (42 to 50% of samples) included predominantly seminomas and was hallmarked by high expression of the testosterone—DHT converting enzyme *SRD5A3*. Hormonal cluster #3 comprised of NSGCT (8.3 to 18% of samples, mixed histology, embryonal carcinoma, yolk sac tumors) with high expression of *CGA* gonadotropin subunit as well as genes involved in estrogen and estradiol synthesis and inter-conversion (*CYP19A1*, *HSD17B12*, *HSD17B1*), and *SHBG* involved in hormone binding and transport. Finally, 23 to 30% of cancer samples were classified as hormonal cluster #4, which consisted primarily of NSGCT (embryonal carcinoma, mixed histology, yolk sac) with a small fraction of seminomas. The key feature of this cluster was increased expression of genes of general steroid (*STAR*, *POMC*, *CYP11A1*, *CYP17A1*, *HSD3B2*) and testosterone biosynthesis (*HSD17B3*) (Fig. 2, Supplementary Figure S4).

### Clinical and pathological data of the hormonal clusters

**Fig. 3:**
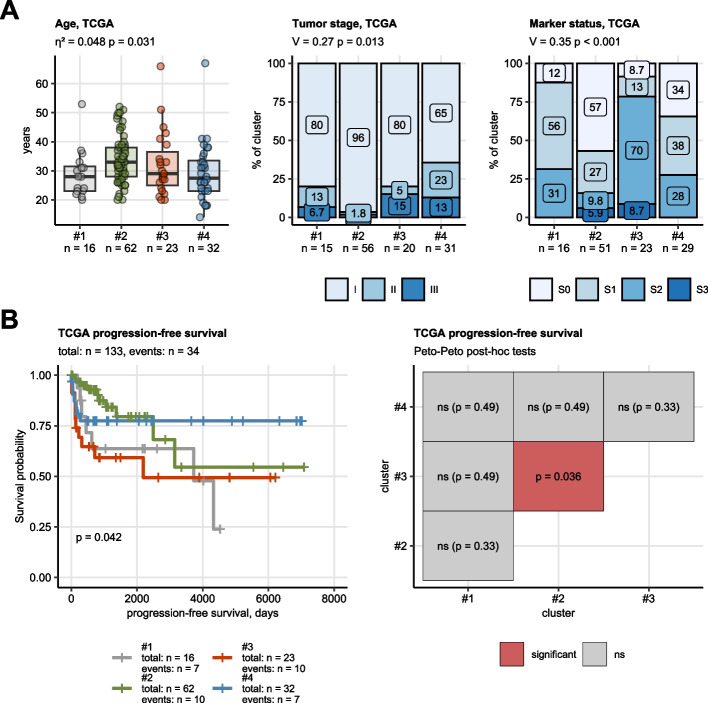
Clinical and prognostic characteristic of the hormonal clusters of testicular cancer. **A** Age at diagnosis (Kruskal–Wallis test, ${\upeta }^{2}$ effect size statistic), tumor stage and serum cancer marker status (${\upchi }^{2}$ test, Cramer’s V effect size statistic) were compared between the clusters. *P* values were corrected for multiple testing with the false discovery rate method. Median age with interquartile ranges is shown in a box plot with whiskers spanning over 150% of the interquartile ranges and single cancer samples visualized as points. Percentages of tumor stages and patients with cancer marker stages in the hormonal clusters are presented in stack plots. Effect sizes and p values are displayed in the plot captions. Numbers of samples in the clusters are indicated in the X axes. **B** General and pairwise differences in progression-free survival between the hormonal clusters were assessed by Peto-Peto tests adjusted for multiple testing with the false discovery rate method. Fractions of surviving patients are visualized in a Kaplan–Meier plot with total numbers of observations and progressions indicated in the plot caption (right panel). Number of observations and progression cases in the hormonal clusters are displayed in the Kaplan–Meier plot legend; p values for the general difference in survival is shown in the plot. *P* values of the pairwise comparison of survival between the hormonal clusters are displayed in a heat map (left panel)

Clinical and pathological features of the hormonal clusters are presented in Fig. 3A. Accordingly, cluster #2 and #3 patients exhibited, respectively, the best and poorest progression-free survival (*p* = 0.042). Analogical yet not significant effects were observed for relapse-free survival (ns (*p* = 0.056)). Hormonal cluster #1 patients were exposed to intermediate-to-high risk of progression and relapse. Progression and relapse risk in cluster #4 was low-to-intermediate (Fig. 3B, Supplementary Figure S5).

In regularized multi-parameter modeling of progression-free survival by RIDGE Cox proportional hazard regression [31, 49], appending of canonical clinical risk factors (age, serum marker status, histological subtype) with the hormonal cluster information had virtually no effect on the model’s accuracy (clinical explanatory factor model: C-index = 0.67, clinical factors and hormonal clusters: C-index = 0.65) but marginally improved its calibration (clinical factors: integrated Brier score [IBS] = 0.22, clinical factors and hormonal clusters: 0.21). In the combined clinical/hormonal cluster model of progression-free survival, S2/S3 marker status, hormonal cluster #1 or #3 assignment, and teratoma histology, were independently associated with roughly 25—30% increase of progression risk (Supplementary Figure S5).

### Gene expression of the clusters

**Fig. 4:**
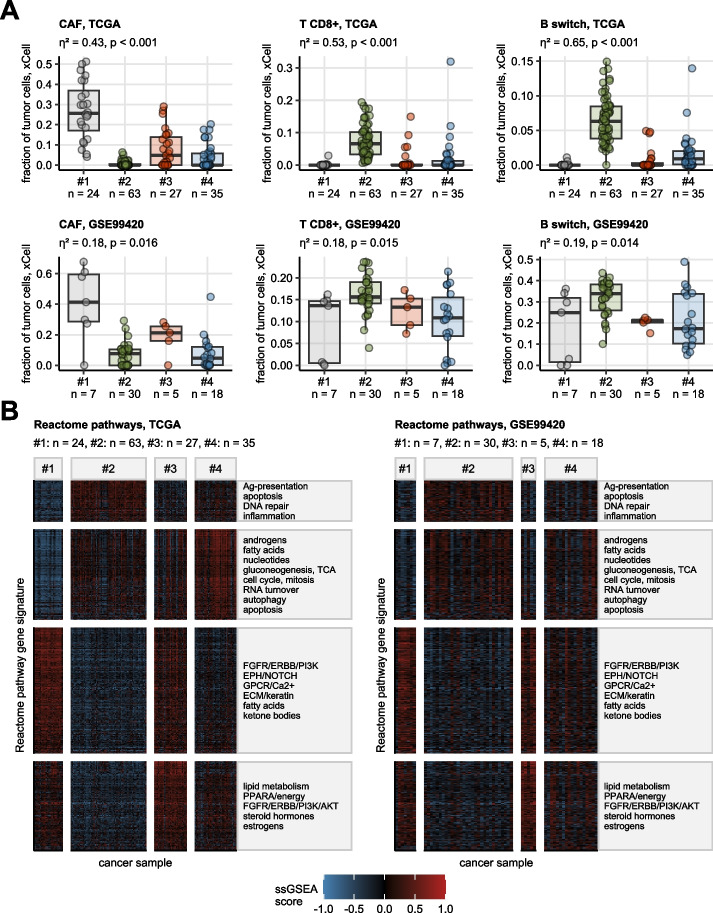
Tumor microenvironment composition and Reactome pathway gene signatures in the hormonal clusters of testicular carcinoma. **A** Fractions of non-malignant cells in cancer samples were estimated by the xCell immunedeconvolution algorithm and compared between the hormonal clusters with Kruskal–Wallis test with ${\upeta }^{2}$ effect size statistic. P values were adjusted for multiple testing with the false discovery rate (FDR) method. Median tumor cell fractions of cancer-associated fibroblasts (CAF), CD8 + T cells, and class-switched B cells with interquartile ranges are visualized as boxes with whiskers spanning over 150% of the interquartile ranges. Single cancer samples are represented by points. Effect sizes and p values are displayed in the plot captions. Numbers of cancer samples in the clusters are indicated in the X axes. **B** Single sample gene set enrichment analysis scores (ssGSEA scores) of the Reactome pathway gene signatures were compared between the hormonal clusters by one-way ANOVA with ${\upeta }^{2}$ effect size statistic. *P* values were corrected for multiple testing with the FDR method. moderate-to-large differences with pFDR < 0.05 and ${\upeta }^{2}\ge 0.14$ were considered significant. ssGSEA scores for gene signatures found to be significant in both the TCGA and GSE99420 cohort are presented in heat maps. Those common significant gene signatures were grouped by unsupervised hierarchical clustering in the TCGA cohort and the signature groups named by their shared biological features. The signature grouping is represented by horizontal facets of the heat maps. Numbers of samples in the hormonal clusters are displayed in the plot captions. Ag: antigen; TCA: tricarboxylic acid cycle/citric acid cycle; GPCR: G protein-coupled receptors; ECM: extracellular matrix; PPARA: peroxisome proliferator activated receptor alpha

As predicted by immunedeconvolution of the gene expression data sets by xCell and MCP Counter algorithms [36, 37], cluster #1 and, to a lesser extent, cluster #3, displayed significantly higher predicted fibroblast levels as compared with clusters #2 and #4. In turn, cluster #2 cancers were predicted to be abundantly infiltrated by T and B cells. Those results were also corroborated in the TCGA cohort by an analysis of cell fractions predicted by the QuanTIseq procedure [38] (Fig. 4A, Supplementary Figure S6).

### Reactome pathways of the clusters

Gene set variation analysis, i.e. comparison of scores of Reactome pathway gene signatures revealed profound differences between the hormonal clusters (all signatures: *n* = 1616, regulated between the clusters in the TCGA and GSE99420 cohorts: *n* = 719). In more detail, extracellular matrix- (ECM) and growth factor signaling-related gene signature scores were the highest in cluster #1 followed by cluster #3. Gene signatures of antigen processing, inflammatory signaling, apoptosis, and DNA repair demonstrated the highest levels in cluster #2. Cluster #3 was characterized by elevated scores of signatures of FGFR/ERBB/PI3K/AKT, lipid metabolism, steroid hormone and estrogen metabolism. Finally, fatty acid, nucleotide, sugar and androgen metabolism along with cell cycle, mitosis, RNA turnover, apoptosis and autophagy were inferred as the key biological processes primarily for cluster #4 (Fig. 4B).

The biological differences between the hormonal clusters were confirmed by an analysis of differential gene expression in particular hormonal clusters as compared with the respective mean gene expression in the TCGA and GSE99420 cohorts. A total of 2000 genes were found to be differentially regulated in cluster #1 as compared with the data set mean in both investigated cohorts; the numbers of such common differentially regulated genes were 2829, 1677, and 468 for clusters #2, #3, and #4, respectively. The top regulated are listed in Supplementary Figure S7.

### GO enrichment analysis of the hormonal clusters

Biological process gene ontology (GO) enrichment analysis was performed for sets of differentially up- or down-regulated genes in particular clusters [50]. Such analysis revealed regulation of genes and biological processes of RNA turnover, organ development, reproduction, proliferation, ECM and mesenchymal cell phenotype in cluster #1. GO terms related to ECM, lipoprotein metabolism, TGF/BMP/WNT signaling, cell adhesion and motility, transcription, organ and tissue development were found significantly enriched among genes up- and downregulated in cluster #2. GO terms significantly enriched in cluster #3 were associated with organ, sex and mesenchymal differentiation, blood coagulation, growth factor and NOTCH/WNT signaling, wound healing, adhesion and motility (Supplementary Figure S8). Of note, we could not identify significantly enriched biological process GO terms in cluster #4 shared by the TCGA and GSE99420 cohort.

### Regulons of the hormonal clusters

**Fig. 5:**
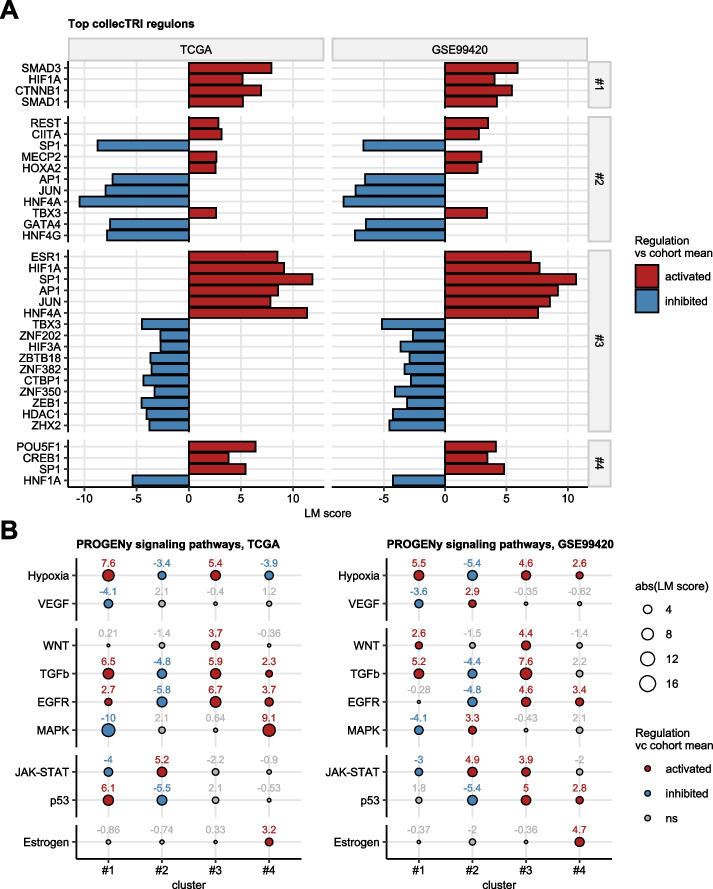
Modulation of transcriptional regulons and signaling pathways in the hormonal clusters of testicular cancer. Differential modulation of the collecTRI model transcriptional regulons and of the PROGENy signaling pathways in the hormonal clusters as comped with the cohort mean was predicted by decoupleR linear modeling tools fed with T statistics of differential gene expression for all available genes. Linear modelling score (LM score) served as an effect size metric of regulon/signaling pathway modulation (LM score < 0: inhibition, LM score > 0: activation as compared with the cohort mean). *P* values (LM score $\ne$ 0) were adjusted for multiple testing with the false discovery rate method. **A** LM scores for top strongest modulated transcriptional regulons in the hormonal clusters (horizontal facets) found to be significant in both the TCGA and GSE99420 cohorts presented in bar plots. **B** LM scores for signaling pathways in the hormonal clusters found to be significantly modulated both in the TCGA and GSE99420 cohorts depicted in bubble plots. Point sizes represent absolute values of the LM scores. Point color codes for regulation sign. Points are labelled with the corresponding LM score values

A subsequent comparison of activity of transcriptional regulons [51, 52], i.e. set of genes regulated by common transcription factors, in the hormonal clusters as compared with the cohort means revealed activity of SMAD1/3-, CTNNB1-, and HIF1A-responsive transcriptome participating among others in ECM deposition, WNT signaling, and hypoxia response in cluster #1. REST, CIITA, MECP2, and HOXA2 regulons associated with neural and embryonal development and inflammation were found to be significantly activated in both the TCGA and GSE99420 cohort. ESR1, HIF1A, SP1, AP1, JUN, and HNF4A were identified as common activated regulons in cluster #3 with functions in estrogen, hypoxia and stress response, and in control of the sex hormone transporter SHBG expression. POU5F1, CREB1, SP1-responsive transcriptome involved in RNA turnover, teratoma formation, and steroid synthesis were found to activated in cluster #4 cancers of the TCGA and GSE99420 cohorts (Fig. 5A). Concerning signaling pathways included in the PROGENy knowledge model [51, 53], high activity of hypoxia and TGF-$\beta$ signaling, and suppression of MAPK and JAK/STAT signaling as compared with the cohort average was predicted for cluster #1 in both the TCGA and GSE99420 cohort based on whole transcriptome expression regulation estimates. Activation of JAK/STAT signaling with concomitant suppression of hypoxia, TGF-$\beta$, EGFR, and p53 signaling was predicted for cluster #2. Cluster #3 was characterized by significantly higher activity of hypoxia, WNT, TGF-$\beta$, and EGFR signaling pathways as compared with the cohort mean. Finally, activation of EGFR and estrogen signaling pathways was predicted for cluster #4 in both investigated testicular cancer collectives (Fig. 5B).

### Immune cell infiltration and inflammatory processes of the hormonal clusters

Immune cell infiltration and inflammatory processes were found to differ between the hormonal clusters. In line with increased infiltration of cluster #2 cancers by T cells, clinically relevant immune checkpoint genes *CTLA4*, *TIGIT*, and *PDCD1* were found to be expressed in this cluster at the highest levels (Supplementary Figure S9).

### CTA analysis of the hormonal clusters

Cancer and testis antigens (CTA) represented a highly prominent group of genes significantly differentially regulated between the hormonal clusters. Out of 1170 CTA genes reported in literature [40, 43], 282 transcripts were expressed at significantly different levels in the hormonal clusters in both the TCGA and GSE99420 cohort. Interestingly, a vast majority of those common regulated CTA transcript exhibited the highest expression levels in cluster #2 followed by cluster #4. By contrast, only few CTA genes were found to be upregulated in cluster #3 (Supplementary Figure S10). This may suggest a normal tissue-like testicular differentiation of cluster #2 seminomas and, in part, cluster #4 malignancies, and extensive de-differentiation of cluster #1 and cluster #3 tumors. This may also reflect high immunogenicity of cluster #2 cancers in line with their T and B cell rich tumor microenvironment [54].

#### Summary of Section 3.5 to 3.9

Gene set variation analysis, GO enrichment analysis and the analysis of the regulons revealed a substantial difference between the clusters. Several genes were found to be regulated differently regarding the clusters, as a hallmark of the biological difference. The GO enrichment analysis demonstrated characteristic results. For example, ECM was shown in cluster #1 and #2, whereas in cluster #4 we could not identify significantly enriched biological process, shared by both the TCGA and GSE99420 cohorts. Regulons demonstrated high hypoxia response in cluster #1 and #3, while in cluster #4 activation of EGFR and estrogen signaling pathways were dominant. Immune checkpoint genes were expressed in cluster #2 at the highest levels. Through the CTA analysis, we identified 282 transcripts being differently expressed within the clusters, with the highest expression levels in cluster #2 and #4, suggesting a de-differentiation of cluster #1 and #3.

### Signaling of the hormonal clusters

ERBB and FGFR signaling were identified as important biological hallmarks of hormonal clusters #1 and #3. In a detailed analysis of ERBB- (*EGFR*, *ERBB2*, *ERBB3*, *ERBB4*) and FGFR- (*FGFR1*, *FGFR2*, *FGFR3*, *FGFR4*) coding genes and genes coding for their ligands, we could observe peak mRNA levels of *EGFR* and *ERBB2* in cluster #1 followed by cluster #3. *FGFR1* and *FGFR4* genes were upregulated in cluster #3 followed by clusters #4 and #2. *FGFR3* was expression was the highest in cluster #2. Among ERBB receptor ligands, solely *NRG3* was differentially regulated in the hormonal clusters and its expression was significantly elevated in cluster #2. Interestingly, FGF-coding genes, *FGF2*, *FGF4*, *FGF5*, *FGF10*, *FGF17*, and *FGF23* were over-expressed in clusters #1, #3, and #4, suggestive of an auto- or paracrine growth-promoting signaling via FGFR1 and FGFR4 (Supplementary Figure S11A).

Regarding transcripts of receptors of gonadotropins and sex hormones, *FSHR* (FSH receptor), *LHCGR* (LH receptor), *ESR2* (estrogen receptor beta), and *AR* (androgen receptor) were found to be differentially regulated between the hormonal clusters (Supplementary Figure S11B). Out of 87 published high confidence estrogen responsive genes [41], 42 were found to be significantly differentially regulated between the hormonal clusters in both the TCGA and GSE99420 cohorts (Supplementary Figure S12A). Among 177 genes [42], mRNA levels of 46 of them differed significantly between the hormonal clusters in both analyzed cohorts (Supplementary Figure S12B). Collectively, the results of the analysis of differential gene expression gonadotropin and sex hormone genes, and estrogen- and androgen-regulated transcriptomes suggest that the FSH—estrogen axis may be active in testicular cancers assigned to hormonal clusters #1 and #3. Analogically, co-expression of the major testosterone regulator LH receptor and genes involved in steroidogenesis and testosterone synthesis indicate that the LH—testosterone circuit may be functional in cluster #4 testicular tumors.

### Tumor microenvironment characteristics of the hormonal clusters

Extensive infiltration by fibroblasts and ECM deposition in cluster #1 and, to a lesser extent, in cluster #3 were inferred from the immune deconvolution data, gene set variation analysis of Reactome pathways, and GO enrichment analysis. We intended to investigate modulation of matrisome, i.e. genes coding for structural proteins of ECM, ECM regulators, and ECM-associated secreted factors [44, 45], between the hormonal clusters. Out of 1001 matrisome genes available for analysis, 333 were found to be significantly differentially regulated between the hormonal clusters in the TCGA and GSE99420 cohort. As shown in Supplementary Figure S13, sizable fractions of collagens (e.g. *COL1A1*, *COL3A1*, *COL5A1*), ECM glycoproteins (e.g. laminins and elastin), and proteoglycans (e.g. *DCN*, *OGN*) were found to be strongly upregulated in cluster #1. By contrast, only few matrisome transcripts were found abundantly expressed in cluster #2. Fibrinogen chain-coding transcripts (*FGA*, *FGB*, *FGG*) as well as transcripts of ECM regulators, e.g. proteases and protease inhibitors (serpins, *ADAMTS18*) were expressed in cluster #3 at highest levels followed by cluster #1. Among few matrisome transcripts specific for cluster #4, secreted factors such as *CSH1*, *GDF3*, *FGF2*, *FGF4*, and *IL23* involved in prolactin, growth factor, and inflammatory signaling, constituted the largest gene set (Supplementary Figure S13).

### Metabolic pathway analysis of the clusters

Activity of RECON2 model metabolic reactions in the hormonal clusters as compared with the respective cohort averages was assessed by a Monte Carlo algorithm provided with differential expression estimates and errors for all available genes [55, 56]. To assess activation or inhibition of metabolic pathways, we analyzed enrichment of RECON metabolic subsystems with significantly activated and inhibited reactions in particular clusters. Collectively, for clusters #1 and #3, a general suppression of oxidative sugar metabolism via citric acid cycle and oxidative phosphorylation can be inferred. A total of 50 reactions of the RECON metabolic subsystems ‘Steroid metabolism’ and ‘Androgen and estrogen synthesis and metabolism’ were predicted to be significantly regulated in at least one of the hormonal clusters of the TCGA and GSE99420 cancers as compared with the respective cohort averages (total reactions: *n* = 104, Supplementary Figure S14).

### Analysis of protein expression of the clusters

**Fig. 6:**
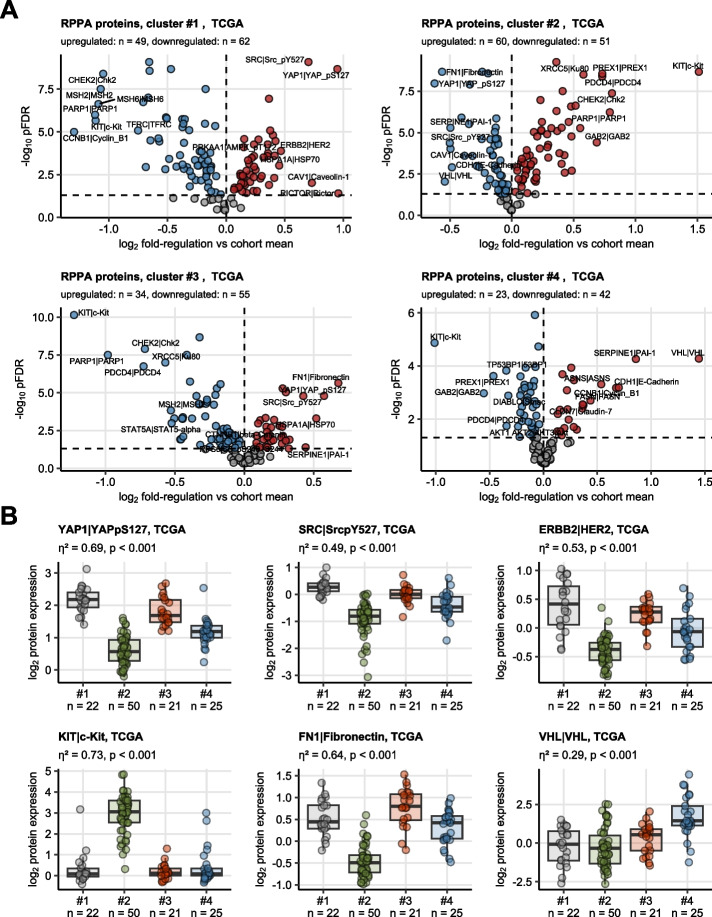
Differential protein expression in the hormonal clusters of testicular cancers in the TCGA cohort. Expression of 194 cancer biology-relevant proteins was investigated in cancer samples of the TCGA cohort with reverse phase protein array. ${\text{log}}_{2}$-transformed expression levels were compared between the hormonal clusters by one-way ANOVA with ${\upeta }^{2}$ effect size statistic. Differences in ${\text{log}}_{2}$-transformed expression between the cluster and the cohort mean were assessed by one-sample T test. *P* values were corrected for multiple testing with the false discovery rate (FDR) method. Proteins with pFDR(ANOVA) < 0.05, ${\upeta }^{2}\ge 0.14$, and pFDR(T test) < 0.05 were deemed differentially regulated. **A** Differences in ${\text{log}}_{2}$ expression between the cluster mean and the cohort mean along with FDR-adjusted p values of the T test are presented in Volcano plots. Each point represents a single gene. Point color codes for significance and regulation sign. Top most strongly regulated genes are labeled with their symbols. Numbers of significantly up- and downregulated proteins are displayed in the plot captions: (**B**) ${\text{log}}_{2}$-transformed expression levels of selected proteins discriminating between the hormonal clusters. Median ${\text{log}}_{2}$ expression values with interquartile ranges are shown as boxes with whiskers spanning over 150% of the interquartile ranges. Cancer samples are visualized as points. Effect sizes and p values for comparison of the clusters with one-way ANOVA are displayed in the plot caption. Numbers of samples in the clusters are indicated in the X axes

For the TCGA cohort cancer samples, levels of 194 proteins of relevance for cancer biology were measured by a reverse phase protein array [29]. Among them, 125 were found to be significantly differentially regulated in at least one hormonal cluster as compared with the respective whole data set average expression. Specifically, in clusters #1 and #3, active phosphorylated forms of SRC and YAP were upregulated. Other proteins of relevance for pro-oncogenic signaling, EGFR and ERBB2 with their active tyrosine phosphorylated forms were found significantly upregulated in cluster #1 and, to a lesser extent, also in cluster #3. In turn, fibronectin protein levels were the highest in cluster #3. Cluster #2 was characterized by increased levels of the KIT oncogene protein and proteins involved in DNA repair and apoptosis (e.g. PARP1, CHEK2). The negative regulator of hypoxia response VHL, protease inhibitor SEPRINE1, and the adhesion molecule E-cadherin were specifically upregulated at protein level in cluster #4 (Fig. 6).

Elevated expression of proteins and phosphoproteins of downstream of EGFR/ERBB in cluster #3 and #1 suggests EGFR/ERBB, SRC, c-RAF/MAPK, and PI3K/AKT/mTOR/RICTOR signaling as a major tumor-promoting circuits of these cancers. Those signaling pathways may be also responsible for downregulation of the pro-apoptotic BIM and BAX proteins [57] in clusters #1 and #3. Of note, in cluster #2, B-RAF, C-RAF, and mTOR proteins were upregulated despite suppression of activated forms of ERBB2 and EGFR. Cluster #2 was also characterized by significant upregulation of pro-apoptotic BIM and BAX proteins (Supplementary Figure S15). In general, upregulation of multiple DNA damage sensors and apoptosis activators at protein levels in cluster #2 is congruent with the Reactome pathway gene signature analysis results and may reflect predisposition of those cancers to cell death inducing signals and anti-cancer treatment.

An analysis of co-expression of proteins in the hormonal clusters #1 and #3 of TCGA testicular cancers underline the relevance of EGF/ERBB, MAPK and AKT/mTOR signaling pathways, whose members were identified as prominent hubs of communities of co-regulated proteins. Interestingly, diverse isoforms of PKC, p38, and JNK were found to be hub proteins in clusters #2 and #4, which may indicate their roles in fueling progression of those testicular cancers. Furthermore, ER-alpha, its phosphorylated form ER-alpha pS118, and AR were also put forward as highly connected proteins in all hormonal clusters (Supplementary Figures S16 and S17). This later finding delivers another piece of evidence of functionality and relevance of sex hormone signaling in testicular cancer.

### Predicted drug response in the hormonal clusters

**Fig. 7:**
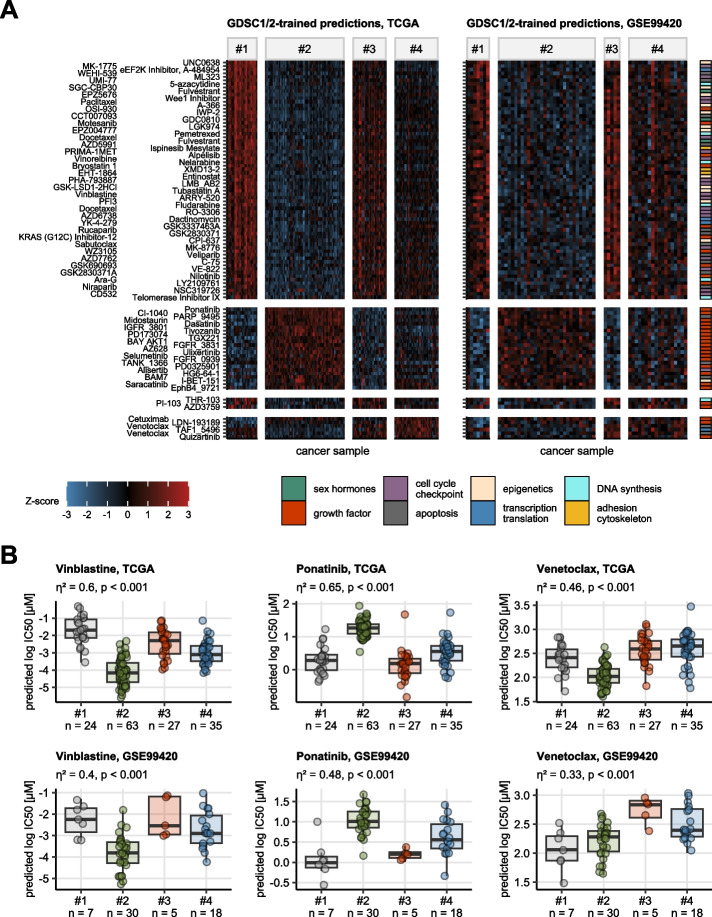
Predicted anti-cancer drug response in the hormonal clusters. Anti-cancer drug response in form of log50 (50% inhibitory concentration) in cancer samples was predicted by RIDGE linear models trained with the GDSC drug screening data set. The log IC50 values were compared between the hormonal clusters by one-way ANOVA with ${\upeta }^{2}$ effect size statistic. Differences in the log IC50 values between the cluster and the cohort mean were assessed by one-sample T test. P values were corrected for multiple testing with the false discovery rate (FDR) method. Compounds with pFDR(ANOVA) < 0.05, ${\upeta }^{2}\ge 0.14$, and pFDR(T test) < 0.05 were considered significant. **A** Normalized log IC50 values (Z-scores) of compounds found to be significant in both the TCGA and GSE99420 cohort are presented in heat maps. The compounds are arranged by their peak expression in the hormonal clusters of the TCGA cohort. Compound classification is color coded in the vertical rug plot. **B** Predicted log IC50 for representative compounds targeting cytoskeleton (vinblastine), growth factor signaling (ponatinib), and apoptosis pathways (venetoclax). Median log IC50 values with interquartile ranges are visualized as boxes with whiskers spanning over 150% of the interquartile ranges. Single cancer samples are depicted as points. Effect sizes and p values for differences between the clusters investigated by one-way ANOVA are displayed in the plot captions. Numbers of samples in the clusters are indicated in the X axes

Among the GDSC-trained data, predicted responses to 124 compounds differed between the hormonal clusters of the TCGA and GSE99420 cohorts. For the CTRP2-trained predictions, differential response to 84 compounds was observed in both collectives. Independently of the training data, cluster #1, and, to a lesser extend cluster #3 cancers, were predicted to be resistant against numerous compounds interfering with DNA synthesis (e.g. folate anti-metabolites: pemetrexed, metothrexate, telomerase inhibitors: MST-312, Telomerase Inhibitor IX), cytoskeleton and cell cycle (e.g. vinblastine, taxane drugs, cell cycle checkpoint inhibitors: rigosertib, BI-2536, MK-1775, PHA-793887, SNS-032, alvocidib), and epigenetics (belinostat, entinostat). Cluster #2 and #4 malignancies were predicted to be, respectively, highly and moderately responsive to that class of drugs. The best response to apoptosis modulators, e.g. obatoclax, venetoclax, PAC-1 or BRD-K35604418, was predicted for cluster #2, while resistance to those substances was predicted for clusters #1, #3, and #4. Of note, this later phenomenon fits well to the increased expression of DNA damage sensors and pro-apoptotic proteins by cluster #2 cancers at both mRNA and protein level. In line with the highly active receptor tyrosine kinase and growth factor signaling, clusters #1 and #3, were estimated to respond well towards tyrosine kinase inhibitors (TKI) such as multi-TKI- and SRC-targeting ponatinib, dasatinib and saracatinib, VEGFR-targeting cabozantinib, foretinib and tivozanib, FGFR inhibitors AZD4547 and PD173074, or EGFR/ERBB inhibitors neratinib and cetuximab. Cluster #2 tumors were in turn predicted to be resistant to such compounds (Fig. 7, Supplementary Figure S18).

#### Summary of sections 3.10- 3.14

According to the signaling of the hormonal clusters, we suggest that the FSH- estrogen axis may be active in cluster #1 and #3, whereas the LH – testosterone circuit may be functional in cluster #4. We demonstrated 333 significantly different regulated matrisome genes, whereas cluster #2 revealed only few abundantly expressed matrisome transcripts. Our data suggests a general downregulation of oxidative sugar metabolism in clusters #1 and #3, affecting both the citric acid cycle and oxidative phosphorylation. The protein expression of several oncogene was significantly upregulated in different clusters: EGFR and ERBB2 in cluster #1 and #3, whereas KIT in cluster #2. In cluster #2, increased protein levels of multiple DNA damage sensors and apoptosis activators align with Reactome pathway gene signature analysis, possibly indicating these cancers' vulnerability to cell death and anticancer therapies. Predictions for 124 compounds varied between TCGA and GSE99420 hormonal clusters within the GDSC-trained dataset.

## Discussion

In this study, we were able to determine four hormonal subsets of TGCT: cluster #1 consisted of mixed NSGCT and teratoma (12 to 16% of samples), cluster #2 including predominantly seminomas (42 to 50% of samples), cluster #3 consisted of mixed histology, embryonal carcinoma, yolk sac tumors (8.3 to 18% of samples) and cluster #4 consisted primarily of NSGCT (embryonal carcinoma, mixed histology, yolk sac) with a small fraction of seminomas (23 to 30% of samples). In the protein analysis and in the metabolic analysis of the clusters we could identify substantial differences between the hormonal clusters. Furthermore, we could find differential predicted drug response in the hormonal clusters, which could bear with clinical significance regarding the therapy of therapy-resistant testicular cancers.

Beside the hormonal clusters we described some substantial differences between NSGCT and seminoma: in both cohorts, the POMC gene, which regulates steroid biosynthesis, along with genes involved in metabolism (HSD17B1, HSD17B2, SRD5A1), and sex hormone transport (SHBG), showed significant upregulation in NSGCT compared to seminoma.

Impaired spermiogenesis, testicular dysgenesis and cryptorchidism are well-described risk factors of testicular cancer [58–60]. Furthermore, the therapy of cryptorchidism involves the administration of hormones, which can improve the later sperm quality as well [17]. According to earlier publications, each histologic subtype has different gene signatures identified by transcriptional profiling: seminomas have overexpressed spermatogenesis-associated genes like PRAME, MAGEA4 and SPAG1 while NSGCTs have overexpressed regulatory genes such as DNMT3B and SOX2 [61, 62]. Based on our data, it appears that both testicular cancers in general and NSGCT specifically possess the necessary machinery for steroid sex hormone metabolism.

It is widely known that seminomas have a better overall survival rate and are less likely to recur than NSGCT [63]. Nonetheless, we demonstrated that hormonal cluster #1 or #3 assignment, similar to S2/S3 marker status or teratoma histology, was independently associated with a notable elevated risk of progression. Angiogenesis, characteristic of NSGCTs has been widely investigated and described [64].

According to earlier publications, testicular cancers that produce HCG completely suppress the gonadotropin response to GnRH at the pituitary level, leading to inhibition of LH and FSH secretion. However, men with HCG negative tumors also have suppressed serum gonadotropin levels, pointing out the possible role of other factors in the suppression of the gonadotropins [65]. Cluster 3# revealed high expression of *CGA* gonadotropin subunit as well as genes involved in estrogen and estradiol synthesis and inter-conversion (*CYP19A1*, *HSD17B12*, *HSD17B1*), and *SHBG* involved in hormone binding and transport.

The third decade of life is when peak incidence for NSGCT occurs, while seminoma patients experience it in their fourth decade [66]. Comparatively, patients in clusters #1 and #3 were significantly younger than the remaining groups.

The “burned-out” phenotype of seminomas is thought to be influenced by the tumor microenvironment (TME), which is made up of various immune cell types, endothelial cells and fibroblasts, and may affect the clinical outcome [67, 68]. The testicular inflammation patterns related to various inflammatory conditions such as hypo-spermatogenesis or seminoma, with or without neoplastic cells have been shown to have a basic difference. T cells were detected in samples with impaired spermatogenesis, while B cells and dendritic cells were hardly present. Alongside T cells, dendritic cells and B cells were also found in high numbers within GCNIS and seminoma. The B cells were organized in cell clusters. There were no mast cells present [69]. Cluster #2, that mainly consisted of seminoma, was predicted to be abundantly infiltrated by T and B cells, furthermore these samples demonstrated the highest level of gene signatures of antigen processing, inflammatory signaling, apoptosis, and DNA repair. The activation of immune cells by seminoma cells creates a powerful pro-inflammatory environment, whereas T cells promote the growth, spread, and stemness of tumor cells [70]. The exceptional clinical outcome of TGCT patients might be due to the crucial role of spontaneous T-cell immunity [71]. Various processes and pathways, such as immunity, extracellular matrix organization, and T cell differentiation were linked to a newly published risk model based on CpG-Pattern. The high-risk group had in a recent study significantly different levels of immune cells than the low-risk group, with higher levels of monocytes, activated NK cells, M2 macrophages, and resting mast cells and lower levels of plasma cells, activated CD4 memory T cells, regulatory T cells, naive B cells and gamma delta T cells [72]. Proinflammatory TME is a well-known aspect of seminomas, but not of NSGCTs. The seminomatous TCam-2 cell line caused a strong activation of immune cells and the release of several pro-inflammatory cytokines, while the non-seminomatous NTERA-2 cell line suppressed T cell and monocyte activation markers and failed to stimulate the secretion of pro-inflammatory cytokines [73]. In line with these findings, we described elevated B cell, regulatory T cells (Treg) and CD8^+^ T cells, effector memory and central memory cytotoxic T lymphocytes in Cluster #2 subset than in the remaining hormonal classes. In line with recent studies, an enhanced immunity level might improve TGCT cancer prognosis [74, 75]. Furthermore, as we previously published, seminomas revealed high IFNG and TNF expression, while the expression of these genes were low in NSGCT [76].

Specifically, in clusters #1 and #3, active phosphorylated forms of SRC and YAP were upregulated. Of interest, this signaling axis was demonstrated to integrate signals from HIPPO, WNT, and growth signaling pathways and drive lung cancer progression [77, 78]. According to previous studies, the interaction between WNT and mTOR signaling influences tumor metabolism, cancer cell growth, and spermatozoa formation [79, 80]. It may be worthwile to mention that the Wnt pathway is considered a driver of noninvasive Sertoli cell tumor [81]. In cluster #2, B-RAF, C-RAF, and mTOR proteins were upregulated despite suppression of activated forms of ERBB2 and EGFR, which may suggest an alternative mode of activation of mTOR and MAPK signaling, e.g. by KIT [82]. Furthermore, Cluster #2 was characterized by increased levels of the KIT oncogene protein, as it consisted of seminomas. Earlier the lack of KIT mutations was described in all NSGCTs [7]. NSGCT showed higher levels of c-MET protein compared to GCNIS and seminoma [83].

Leydig cells produce testosterone, which can be transformed into estradiol via aromatase (CYP19A1) [24]. Compared to the surgery only group, post-orchiectomy cytotoxic treatments like radio or chemotherapy result in decreased testosterone levels in TGCT patients. Polymorphisms of the LH-Receptor, the androgen receptor or the 5a-reductase II gene can play a role in the different post treatment hormone levels [84]. We have described a different regulation of FSHR, ESR2 and AR between the hormonal clusters, whereas *LHCGR* was detected at the highest levels in cluster #4. Estrone is converted to the potent ligand estradiol by HSD17B1 in estrogen synthesis [85]. Along this enzyme the genes involved in estrogen and estradiol synthesis and inter-conversion were hallmarked in cluster #3. It has been reported that the single nucleotide polymorphisms (SNP) of CYP1A1 and HSD17B1 were significantly linked to NSGCT histology, being associated with a worse prognosis, as we previously mentioned [86]. According to these findings, we found that hormonal cluster 1# or 3# assignment was independently associated with worse prognosis. The polymorphism of HSD17B4 and CYOP1A1 genes might increase the likelihood of developing TGCT, particularly seminoma over NSGCT [13]. The risk of TGCT might be affected by chlordane’s and polychlorinated biphenyls when combined with certain CYP1A1 and HSD17B4 polymorphisms [15].

AFP can be only expressed by NSGCT, while HCG can be produced by seminomas and NSGCTs, as well [87]. AFP gene was strongly up-regulated in cluster #3, as well.

In case of relapse or therapy refractory tumors there a salvage therapy with high-dosis chemotherapy can be indicated, however, with poor outcome [1, 88]. As we demonstrated, hormonal clustering of testicular cancer might add some therapeutic choice in form of personalized therapy: Cluster #2 and #4 malignancies were predicted to be highly and moderately responsive to numerous compounds interfering with DNA synthesis cytoskeleton and cell cycle. The best response to apoptosis modulators, e.g. obatoclax, venetoclax, PAC-1 or BRD-K35604418, was predicted for cluster #2. Of note, this later phenomenon fits well to the increased expression of DNA damage sensors and pro-apoptotic proteins by cluster #2 cancers at both mRNA and protein level. Clusters #1 and #3, were estimated to respond well towards (TKI). We described substantial differences in the TME of the hormonal clusters. There appears to be a close relationship between the TME and the chemoresistance of TGCT [89].

The most important limitation of the analyses with the TCGA testicular cancer dataset concerned the hormonal subset assignment done with Gaussian mixture modeling. Notably, sex hormone-related genes used in modeling were hand-picked and the classification scheme may change with inclusion of other transcripts, e.g. of genes involved in degradation of the sex hormones and their receptors.

### Perspectives and Significance

Results of the survival analysis clearly demonstrate the potential of sex hormone-related gene expression for prediction of progression, resulting in distinct patterns of tumor biology, TME composition, metabolism and immunity between the subgroups. Clustering by sex hormone-related genes, we identified cluster #1 or #3 as an independent prognostic factor for poor progression-free survival. Thus, varying expression of the sex-hormone genes correlates with significant differences in the TME composition, signaling and metabolism. Novel targeted therapies according to the hormonal clusters may provide a further step towards personalized medicine.

## Conclusions

Hormonal cluster #1 or #3 assignment is a poor prognostic factor regarding survival. Hormonal clustering may pose an attractive novel therapeutic approach to further improve patient outcome introducing personalized treatment also in TGCT.

## Supplementary Information

Supplementary Material 1.

Supplementary Material 2: Furthermore,“Supplementary Material” is as a PDF file also available with a detailed description of the Methods ( Software; Data sources; Descriptive statistics, significance and effect size; Differential expression of sex hormone-related genes in histologies of testicular cancers; Co-regulation networks of sex hormone-related genes; Clustering of testicular cancer samples in respect to sex hormone-related gene expression; Demographic, clinical, pathological, and prognostic characteristic of the clusters; Tumor microenvironment of the hormonal clusters; Gene set variation analysis for Reactome pathway gene signatures; Differential gene and protein expression, and differences in predicted drug sensitivity; Protein co-regulation networks; GO enrichment analysis and semantic clustering of GO terms; Transcriptional regulons and signaling; Metabolic reaction and metabolic subsystem activity; Genetics of the hormonal clusters; Data and code availability). Supplementary Tables and Supplementary Figures regarding detailed results of our analysis are involved, as well.

## Data Availability

Data from publicly available sources were analyzed. The R analysis pipeline is available as a GitHub repository. No datasets were generated or analysed during the current study.

## Abbreviations

AR: Androgen receptor
AUC: Area under the dose–response curve
BIC: Bayesian information criterion
CTA: Cancer and testis antigens
DHT: Dihydro-testosterone
ECM: Extracellular matrix
ESR2: Estrogen receptor beta
FDR: False discovery rate
FSH: Follicle stimulating hormone
FSHR: FSH receptor
GCNIS: Germ cell neoplasia in situ
GO: Gene ontology
GSVA: Gene set variance analysis
IBS: Integrated Brier score
ICD-O: International classification of diseases for oncology
KIT: KIT Proto-Oncogene, Receptor Tyrosine Kinase
LH: Luteinizing hormone
LHB: Luteinizing hormone subunit beta
LHCGR: LH receptor
NSGCT: Non-Seminomatous Germ Cell Tumor
RFS: Relapse-free survival
SGCT: Seminomatous Germ Cell Tumor
SNP: Single nucleotide polymorphisms
ssGSEA: Single sample gene set enrichment analysis
TCGA: The Cancer Genome Atlas
TGCT: Testicular germ cell tumor
TKI: Tyrosine kinase inhibitors
TME: Tumor microenvironment
Treg: Regulatory T cells
