# Supplementary material for: Biological and therapeutic implications of sex hormone-related gene clustering in testicular cancer
Source: Basic Clin Androl. 2025 Feb 26;35:8. doi: 10.1186/s12610-025-00254-5 (PMC11863433; doi:10.1186/s12610-025-00254-5)
Supplement: Supplementary file 1 — Supplementary Material 1. [file 12610_2025_254_MOESM1_ESM.pdf]

Supplementary Material

Methods

Software

Analyses were done with R version 4.2.3 with the *tidyverse* environment for data transformation and visualization (1). Meta-programming and handling of data frames and lists was supported by *rlang* (2) and *trafo*, respectively. Handling of stings was accomplished by *stringi* (3).

For exploratory data analysis, statistical hypothesis testing, effect size estimation, and differential gene, protein and drug sensitivity regulation, packages *ExDA* and *microViz* were used, which implement functions provided by base R, *stats*, *rstatix* (4), *rcompanion* (5), *caret* (6), and *DescTools* (7). Predictions of drug responses for cancer samples were made with *htGLMNET*, which internally utilizes regularized linear modeling algorithms of *glmnet* (8).

For co-regulation network analyses, packages *igraph* (9) and *ggnetwork* (10) were utilized. Clustering analysis was done with package *clustTools*, which uses among others algorithms genuinely implemented by base R, *stats*, *cluster* (11), and *clusterHD* (12). Cluster predictions were done with the Random Forest algorithm provided by *ranger* (13). For survival analyses and regularized Cox modeling, packages *survival* (14), *survminer* (15), *rms* (16), and *coxExtensions* were used. Modeling of *collecTRI* transcriptional regulon and *PROGENy* signaling pathway activity was accomplished by *decoupleR* (17–19). Modeling of activity of RECON2 metabolic reactions (20,21) was done with *biggrExtra*.

Visualization of the analysis results was done with packages *ggplot2* (dot, scatter, bar, and rug plots), *ExDA* (box and stack plots), *microViz* (Volcano and Forest plots, heat maps), *ggnetwork* (network visualizations), and *survminer* (Kaplan-Meier plots). Tables and figures of the manuscript and Supplementary Material were generated with *flextable* (22) and *cowplot* (23). Parts of the manuscript and Supplementary Material were written in *rmarkdown* environment (24) with *bookdown* package (25), and rendered as Word files with *knitr* (26).

Data sources

Two published cohorts of testicular cancers were retrieved from the cBioportal and GEO repositories (search criteria: availability of normalized transcriptome data, at least 50 cancer samples) and were analyzed in the current report: TCGA (27) and GSE99420 (28). The search returned also another collective, GSE3218, which, however, due to a low

frequency of seminomas (11%, total samples: n = 107), did not yield congruent differential gene expression and clustering results with the TCGA and GSE99420 cohorts and was not presented in the current report.

The TCGA cohort consisted of 149 cancer samples (seminoma: 43%, nonseminomatous germ cell tumor [NSGCT]: 57%) with clinical information, RNA sequencing of the transcriptome, somatic mutation, gene amplification and deletion data. Furthermore, expression levels of 194 proteins of relevance for tumor biology measured by a reverse phase protein array were provided for the TCGA cohort cancers. The clinical information encompassed data on age and ethnicity of the patient, histology, pathological and marker staging, total mutation burden (TMB) and microsatellite instability (MSI) of the tumor, as well as relapse- and progression-free survival. The GSE99420 cohort consisted of 60 cancer samples (seminoma: 50%, NSGCT: 50%) for which transcriptome data obtained by a microarray measurement (Illumina HumanHT-12 WG-DASL V4.0 R2 expression beadchip) as well as information of tumor histology and presence of relapse during the study follow-up was provided. Demographic and clinical characteristic of the cohorts is provided in **Table 1**.

RSEM and quantile-normalized transcriptome data provided by the study data were expressed as $log_2(counts + 1)$ and $log_2(signal)$ for the TCGA and GSE99420 data sets, respectively, and used in the analyses.

Based on the transcriptome data, fractions and counts of infiltrating non-malignant cells in the tumor samples were computed with the xCell, MCP Counter, and QuanTIseq immunedeconvolution algorithms (function `deconvolute()`, R package *immunedeconv*) (29–32).

Estimates of anti-cancer drug response for single tumor specimens based on the whole-transcriptome data were obtained with RIDGE regularized linear regression with R package *htGLMNET* implementing a fast modification of the previously published algorithm *pRRophetic* (8,33,34). The linear models used for prediction of log IC50 (50% inhibitory concentration) and AUC (area under the dose response curve) were trained, respectively, with results of the GDSC (35) and CTRP2 (36) in vitro drug screening experiments. The training data sets were restricted to cell lines derived from solid, epithelial-origin cell lines. Reliable predictions were considered for linear models characterized by cross-validated $R^2 \geq 0.13$ and correlation between the observed and predicted response of $r \geq 0.5$ in the training data. Applying those model selection criteria, reliable predictions were obtained for 305 compounds in the GDSC experiment, and 214 substances in the CTRP2 data set.

Genes associated with Reactome pathways were extracted from a local copy of the MSig database (version 7.5.1) with a regular expression term `^REACTOME`. Single sample gene set enrichment analysis scores (ssGSEA scores) for those gene signatures were computed with function `calculate()` provided by R package *gseaTools*, which implements the original algorithm by Hänzelmann and colleagues (37).

Genetic data for the TCGA cohort, i.e. presence of at least one somatic mutation, amplification, or deletion of a protein-coding gene were binarized (0: no alteration, 1: alteration present). Protein expression levels were analyzed in log_2-transformed form.

The sex hormone-related genes investigated in the current report included pituitary gonadotropins, and genes involved in steroidogenesis, formation, binding, and catabolism of estrogens and androgens. The respective gene symbols and Entrez identifiers were extracted from 'Metabolism of steroid hormones', 'Estrogen biosynthesis', and 'Androgen biosynthesis' Reactome pathways and are listed in **Table 2**. Three genes genuinely included in the Reactome pathways of steroid hormone metabolism, *FSHB*, *CYP11B1*, and *CYP11B2* exhibited negligible variability between cancer samples as measured by Gini coefficient and low median expression, and were hence excluded from further analyses. log_2-transformed expression levels of the sex hormone-related genes were adjusted for the cohort/batch effect by the ComBat algorithm (38) prior to comparison of expression between histological strata, co-regulation network analysis, and clustering (function `pre_process()`, package *htGLMNET*).

Cancer and testis antigen genes were derived from a publication by Wang at al. and the CTpedia database (39,40). High confidence estrogen responsive genes (late, intermediate and early transcripts) and transcripts whose levels were described to be regulated by androgens in prostate cancer were extracted from literature reports (41,42). Annotation and classification of ECM-related genes, so called matrisome, were obtained by with the `matriannotate()` function from *MatrisomeAnalyzeR* package (43,44). Other genes of interest such as ERBB or immune checkpoint features were fetched by a manual search in the NCBI Gene database.

Descriptive statistics, significance and effect size

Descriptive statistics presented for numeric variables were medians, interquartile ranges, ranges and numbers of complete observations. For qualitative variables, percentages and counts of observations in the categories were shown .

Normality and variance equality of numeric variables were routinely tested with Shapiro-Wilk and Levene test, respectively (functions `explore()` and `compare_variables()`, *ExDA*). Statistical significance for differences in numeric variables between strata were investigated by two-tailed T tests, Mann-Whitney tests, Kruskal-Wallis tests, and one-way ANOVA with Cohen's d, r and η^2 effect size statistics. Statistical significance for differences in distribution of categories of categorical variables between strata was determined by χ^2 test with Cramer's V effect size statistics. Statistical significance for correlations was assessed by Spearman's rank and Pearson's test with ρ and r correlation coefficients. `compare_variables()` and `correlate_variables()` from *ExDA* were used for statistical tests of comparisons and correlations.

Effects size metrics and model performance statistics were interpreted as follows (45–47):

- *r effect size statistic for Mann-Whitney test*: small effect: r < 0.3, moderate: 0.3 ≤ r < 0.5, large: r ≥ 0.5
- *Cohen's d effect size statistic for T tests*: small effect: d < 0.5, moderate: 0.5 ≤ d < 0.8, large: d ≥ 0.8
- η^2 *effect size for Kruskal-Wallis test and one-way ANOVA*: small effect: $\eta^2 < 0.06$, moderate: $0.06 \leq \eta^2 < 0.14$, large: $\eta^2 \geq 0.14$
- *Pearson's r and Spearman's ρ correlation coefficients*: r < 0.3, moderate: 0.3 ≤ r < 0.5, large: r ≥ 0.5
- *Cramer's V effect size statistic for* χ^2 *tests*: V < 0.3, moderate: 0.3 ≤ V < 0.5, large: V ≥ 0.5
- R^2 *measure of explained variance*: small effect: $R^2 < 0.13$, moderate: $0.13 \leq R^2 < 0.26$, large: $R^2 \geq 0.26$

If not indicated otherwise, p values were adjusted for multiple comparisons with the false discovery rate (FDR) method (48) separately for the analysis step and cohort (e.g. separate adjustments for differential gene expression analyses in the TCGA and GSE99420 collectives). Effects with p < 0.05 were considered significant.

Differential expression of sex hormone-related genes in histologies of testicular cancers

ComBat-adjusted log_2-transformed mRNA levels of some of the sex hormone-related genes in the tumor tissue (e.g. *HSD3B1* or *CGA*) were strongly non-normally distributed as assessed by Shapiro-Wilk test. For this reason, expression of the sex hormone-related genes in cancer histologies was explored by non-parametric tests. Differential gene expression between seminoma and NSGCT in the TCGA and GSE99420 cohort was compared by Mann-Whitney test with r effect size statistic (**Supplementary Table S1**). Detailed ICD-O classification of tumor histologies was provided only for TCGA cohort specimens. Differences in gene expression between seminoma (SEM), mixed germ cell tumors (MGCT), embryonal carcinoma (EMBCA), teratomas (TT, consisting of benign and malignant teratomas and teratocarcinomas), and yolk sac tumors (TYST) were assessed by Kruskal-Wallis test with η^2 effect size statistic (**Supplementary Table S2**).

Co-regulation networks of sex hormone-related genes

Co-regulation of expression of the sex hormone-related genes in the cancer tissue was explored by pairwise Spearman's ρ correlation coefficients. Subsequently, correlation matrices were analyzed and visualized as graph structures with tools provided by *igraph* (9) and *ggnetwork* packages (10).

For ComBat-adjusted mRNA levels positively correlated with at least moderate effect size, i.e. $\rho \geq 0.3$, graph objects were constructed using a wrapper around the function `graph_from_adjacency_matrix()`. The graph structures were plotted as dots representing the nodes, i.e. sex hormone-related genes, and edges representing correlations of mRNA levels with $\rho \geq 0.3$. Functional classification of the genes (**Table 2**) was coded by node color. Edge colors and widths represented ρ values (functions `geom_nodes()`, `geom_edges()`, `geom_nodelabel_repel()`).

The following importance statistics were computed for the nodes: degree (number of edges projecting from the node), betweenness (number of shortest paths between node pairs crossing the node of interest divided by the total number of shortest paths), and hub score (eigenvector of the matrix of correlations with $\rho \geq 0.3$) with the functions `degree()`, `betweenness()`, and `hub_score()`, respectively (**Supplementary Table S3**).

Clustering of testicular cancer samples in respect to sex hormone-related gene expression

In clustering analyses, functions provided by R packages *clustTools* (clustering and evaluation of clustering structures) and *ranger* (cluster assignment prediction) (13) were employed.

Testicular cancer samples in the TCGA training cohort were classified into four clusters (named 'hormonal clusters') in respect to their ComBat-adjusted log_2-transformed mRNA levels of the sex hormone-related genes (**Table 2**) by the hard threshold-regularized KMEANS algorithm (12). Choice of the regularization parameter λ = 0.15 which controls the number of cluster-defining factors was motivated by the minimum of Bayesian information criterion (BIC) (12). The criterion of selection of the cluster k was the maximum of mean silhouette width, i.e. a metric that reflects cluster separation (49) (**Supplementary Figure S3A**). The function `htk_cluster()` was used to construct the clustering object.

Prediction of cluster assignment of cancer samples in the GSE99420 test collective was done with a 500-tree Random Forest classifier with the ComBat-adjusted log_2-transformed expression levels of the sex hormone-related genes serving as explanatory factors (a wrapper around the `ranger()` function) (13). The optimal set of parameters of the classifier, `mtry` = 3 (number of randomly selected explanatory variables per tree), `min.node.size` = 1 (minimal number of observations in the tree's terminal nodes), and Gini splitting rule, was selected by minimizing the out-of-bag classification error. The Random Forest-generated cluster assignment predictions were subsequently turned into a clustering analysis object with the `clust_analysis()` function.

Performance of the clusters in the training TCGA cohort and the test GSE99420 collective was assessed with the `summary()` method, which returns metrics of cluster separation (mean silhouette width), misclassification rate (fraction of observations with negative

silhouette widths), explained clustering variance (ratio of the total between-cluster sum of squares to the total sum of squares), and neighborhood preservation (fraction of the five nearest neighbors assigned to the same cluster) (49,50). The performance statistics are listed in **Supplementary Table S4**.

Quality of the clustering solutions was also explored by comparison of cluster size distribution between the test and training cohort by χ^2 test with Cramer's V effect size statistic (**Supplementary Figure S3B**). Furthermore, to assess separability and similarity of single clusters, we compared pairwise sum-of-square distances between observations in the training and test cohort. Similarity of the corresponding clusters (e.g. cluster #1 in the TCGA cohort and cluster #1 in the GSE99420 cohort) and separation of the non-corresponding clusters in the training and test cohorts (e.g. cluster #1 in the TCGA cohort and cluster #2 in the GSE99420 cohort) were investigated by comparison of sum-of-square cross-distances. The distances were computed with the function `cross_distance()` and visualized as heat maps with the method `plot(type = 'mean')` (**Supplementary Figure S3C**).

In the final step of quality control of the clustering structures, we compared ComBat-adjusted log_2-transformed mRNA levels of the cluster-defining sex hormone-related genes between the clusters with Kruskal-Wallis test with η^2 effect size. In this analysis, all clustering genes differed significantly between the clusters in the training TCGA cohort. In the GSE99420 test collective, expression levels of 19 out of 34 cluster-defining genes varied significantly between the hormonal clusters (**Supplementary Table S5**).

Demographic, clinical, pathological, and prognostic characteristic of the clusters

Differences in demographic, clinical, and pathological features between the hormonal clusters were explored by χ^2 and Kruskal-Wallis tests with, respectively, Cramer's V and η^2 effect size statistics (**Supplementary Table S6**).

Survival information was provided solely for the TCGA cohort. Global and pairwise differences in relapse- and progression-free survival between the hormonal clusters were assessed by Peto-Peto tests and visualized in Kaplan-Meier plots (functions `surv_fit()`, `surv_pvalue()`, `ggsurvplot()`, packages *survival* and *survminer*) (14,15).

Effects of hormonal cluster assignment on progression-free survival in a wider context of canonical risk factors (age, serum marker staging, ICD-O histological subtyping) were modeled in the TCGA collective by RIDGE Cox regression (51) using tools implemented by *survival*, *rms*, *glmnet* (8,14,16), and *coxExtensions* packages. Three RIDGE Cox models were constructed: (1) a model with hormonal cluster assignment as the sole explanatory variable, (2) a model with clinical prognostic factors (age, serum marker stage, ICD-O histological subtypes), and (3) a model with hormonal cluster assignment and clinical prognostic factor as explanatory variables. The optimal values of the regularization parameters (model 1: λ = 0.012, model 2: λ = 0.2, model 3: λ = 0.2) were obtained by

minimizing the model's deviance in 200-repeats 5-fold cross-validation (function `cv.glmnet()`). Subsequently, `glmnet` models were constructed for the optimal λ values and evaluated. Harrell's concordance index served as a measure of concordance between the predicted and observed progression-free survival times (52), integrated Brier score was used to gauge model's calibration (53), R^2 was utilized to measure variance explained by the model (**Supplementary Table S7**). For the evaluation of Cox model performance, functions `glmnet()`, `coxph()`, `as_coxex()`, and `summary()` were used. RIDGE `glmnet` model coefficient estimates were extracted with the `coef()` method, transformed to hazard ratios (HR) and visualized in a bar plot.

Tumor microenvironment of the hormonal clusters

Fractions (xCell, MCP counter) and counts (MCP Counter) of non-malignant cells in cancer samples predicted by immunedeconvolution (30–32) were compared between the hormonal clusters by Kruskal-Wallis test with η^2 effect size statistic (**Supplementary Table S8**).

Gene set variation analysis for Reactome pathway gene signatures

Differences in ssGSEA scores of Reactome pathway gene signatures between the clusters were explored by one-way ANOVA with η^2 effect size statistic (function `test_anova()`, package *microViz*,). Gene signatures differing between the clusters with pFDR < 0.05 and $\eta^2 \geq 0.14$ in both cohorts (i.e. significant, moderate-to-large difference, 719 out of 1615 gene signatures) were deemed common significant gene signatures (**Supplementary Table S9**). The common significant gene signatures were grouped by similarity of expression of their ssGSEA scores in the TCGA cohort by Ward's hierarchical clustering (function `hcluster()`, k = 4 clusters, cosine distance between signatures). The resultant signature subsets were named by their key biological processes and visualized in heat maps.

Differential gene and protein expression, and differences in predicted drug sensitivity

Significantly differentially expressed genes (TCGA and GSE99420) and proteins (TCGA only), and drugs (TCGA and GSE99420) differing in predicted response in the hormonal clusters were identified with a two step procedure operating with log_2-transformed expression levels for genes and proteins, and log IC50 or AUC for drug sensitivity predictions. First, the difference between the hormonal clusters was assessed by one-way ANOVA with η^2 effect size statistic (function `test_anova`, package *microViz*). Second, the difference between the cluster mean of a gene or protein expression, or sensitivity metric and the respective mean value in the entire cohort was investigated by one-sample T test (function `avg_deviation()`, package *microViz*). Features with pFDR(ANOVA) < 0.05, $\eta^2 \geq$

0.14, and pFDR(T test) < 0.05, which translates to a significant moderate-to-large difference between the hormonal clusters and significant departure from the cohort's average, were considered significantly differentially regulated. The differential expression and differential drug sensitivity analysis results are listed in **Supplementary Tables S10**, **S16**, and **S19**.

Protein co-regulation networks

log_2-transformed expression levels of 194 cancer biology-relevant proteins were measured by reverse protein arrays for TCGA cancer specimens (27). Co-regulation of those protein amounts in each hormonal cluster was explored by Pearson's r correlation coefficients and graph analysis essentially as described for the co-regulation networks of the sex hormone-related genes. In brief, graph objects were generated for log_2-transformed protein levels positively correlated with at large effect size, i.e. r ≥ 0.5. Communities of tightly co-regulated proteins were identified in each hormonal cluster by the function `cluster_edge_betweenness()` (package _igraph), which iteratively removes edges with the largest betweenness statistic (i.e. number of shortest paths between node pairs passing via the edge of interest) till a maximum of modularity statistics Q is reached (9,54). The modularity statistics for the community solutions in the clusters were: cluster #1: Q = 0.53, cluster #2: Q = 0.62, cluster #3: Q = 0.47, and cluster #4: Q = 0.5 indicative of informative division of the protein co-regulation network (54). The graph objects with node color coding for protein community assignment, and edge size and color representing values of r correlation coefficient were visualized as described for the sex hormone-related gene networks.

GO enrichment analysis and semantic clustering of GO terms

Genes found to be differentially regulated between the hormonal clusters were subjected to biological process gene ontology (GO) enrichment analysis employing the `goana` algorithm originally implemented by *limma* package (function `GOana()`, package *microViz*). Effect size of enrichment was measured by odds ratio (OR) defined as

$$OR = \frac{N_{GODGE} \times N_{universe}}{N_{GO} \times N_{DGE}}$$

where N_{GODGE} represents the number of differentially regulated genes assigned to the particular GO term, $N_{universe}$ stands for the total number of investigated genes, N_{GO} represents the total number of genes assigned to the particular GO term, and N_{DGE} indicates the number of differentially regulated genes. Significantly enriched GO terms in the hormonal clusters shared by the TCGA and GSE99420 cohorts are listed in **Supplementary Table S11**.

Those common significantly enriched GO terms in each hormonal cluster were subsequently subjected to clustering in respect to pairwise semantic Wang distances between the terms (55,56) (function `go_sem()`, *microViz*).
In more detail, the matrices of Wang distances were subjected to two-dimensional scaling (multi-dimensional scaling [MDS], function `reduce_data()`, package *clustTools*). Next, the MDS layouts were clustered with the PAM algorithm (function `kcluster(clust_fun = 'pam')`, package *clustTools*). The resultant clusters of semantically similar GO terms were named after their key biological features.

Transcriptional regulons and signaling

Modulation of *collecTRI* model transcriptional regulons, i.e. sets of genes whose expression is orchestrated by a common transcription factor, and of *PROGENy* model signaling pathways was investigated by uni- and multi-variable linear modeling tools provided by package *decoupler* (functions `ulm()` and `mlm()`) (17–19). The linear models were fed with T statistic values of regulation of gene expression in the cluster as compared with the respective cohort average expression obtained for all available genes. Sign and magnitude of modulation of a regulon or signaling pathway was gauged by linear modeling score (LM score) (18). P values ($p(LM \neq 0)$) were corrected for multiple testing with the FDR method. Regulons and signaling pathways found to be significantly activated or inhibited in the hormonal clusters both in the TCGA and GSE99420 collectives are listed in **Supplementary Tables S12** and **S13**.

Metabolic reaction and metabolic subsystem activity

Activity of RECON2 model metabolic reactions in the hormonal clusters as compared with the respective average activities in the cohort were modeled by Monte Carlo simulations with *biggrExtra* package as reported by us previously (57,58). In brief, the Monte Carlo models were constructed with log_2 fold-regulation estimates of mRNA levels in the clusters as compared with the cohort average and their standard errors computed for all available genes. log_2 fold-regulation estimates of metabolic reaction activity with 95% BCA confidence intervals were obtained by evaluation of the gene - reaction assignment rules in n = 3000 random draws from normal distribution of gene expression regulation estimates (function `build_geneSBML()`). P values of reaction activity regulation ($p(log_2 fold - regulation \neq 0)$) were corrected for multiple comparisons with the FDR method separately for each cluster and cohort. Activated and inhibited reactions in the hormonal clusters as compared with the cohort's means found to be significant in both the TCGA and GSE99420 cohorts are listed in **Supplementary Table S14**.

Enrichment of RECON metabolic subsystems with significantly activated or inhibited reactions was assessed by comparing frequencies of significantly activated/inhibited reactions assigned to a particular subsystem with 10000 random draws from the entire reaction pool (function `suba()`). Because floors of the p values was strictly dependent on

the number of random draws in this kind of simulation test, no multiple testing correction was applied. Effect size of the metabolic subsystem enrichment was quantified with an odds ratio statistic defined as

$$OR_i = \frac{N_{reg,i} \times N_{universe}}{N_i \times N_{reg}}$$

where $N_{reg,i}$ stands for the number of significantly activated or inhibited reactions assigned to the particular subsystem i, $N_{universe}$ represents the total number of reactions, N_i represents the total number of reactions in the subsystem i, and N_{reg} is the total number of significantly activated or inhibited reactions. Metabolic subsystems found to be significantly enriched with activated or inhibited reactions in the corresponding clusters of the TCGA and GSE99420 cohorts are listed in **Supplementary Table S15**.

Genetics of the hormonal clusters

Information of somatic mutations, deletions, and amplifications of protein-coding genes was available for the TCGA cohort (27) and was coded and analyzed in binarized form (i.e. any mutation/amplification/deletion of a gene coded as 1, lack of alteration/WT form of a gene coded as 0). Total mutation burdens (TMB), as well as microsatellite instability (MSI) SENSOR and MANTIS scores were provided for TCGA cancer specimens as well (27).

Differences in TMB, MSI scores, and total numbers of mutations, deletions and amplification of protein-coding genes were compared between the hormonal clusters by Kruskal-Wallis test with η^2 effect size metric (**Supplementary Table S17**). Differences in frequency of particular mutations, deletions or amplifications between the hormonal clusters were investigated by χ^2 test with Cramer's V effect size statistic (**Supplementary Table S18**).

Data and code availability

Data from publicly available sources were analyzed. The R analysis pipeline is available as a GitHub repository.

Supplementary Tables

Supplementary Table S1: Differential expression of hormone-related genes in seminoma and NGSCT in the TCGA and GSE99420 cohorts. Log2-transformed expression levels are presented as medians with interquartile ranges and ranges. Significant effects are shown. The full table is available as a supplementary Excel table.

Cohort	Variable	Seminoma	NSGCT	Significance[a]	Effect size[a]
TCGA	Samples, N	62	82		
	GNRH1	6 [IQR: 5.7 - 6.4]<br>range: 5.2 - 7.4	6.4 [IQR: 6 - 6.7]<br>range: 5.1 - 7.3	p = 0.0094	r = 0.23
	PRL	3.1 [IQR: 3.1 - 3.1]<br>range: 3.1 - 4.6	4 [IQR: 3.5 - 5]<br>range: 3.1 - 9.6	p < 0.001	r = 0.59
	CGA	2.7 [IQR: 2.7 - 2.7]<br>range: 2.7 - 11	5.1 [IQR: 3.6 - 8]<br>range: 2.7 - 13	p < 0.001	r = 0.65
	LHB	5.4 [IQR: 5 - 5.8]<br>range: 3.4 - 7.8	4.4 [IQR: 4 - 4.8]<br>range: 3.4 - 7.3	p < 0.001	r = 0.54
	POMC	7 [IQR: 6.3 - 7.8]<br>range: 4.9 - 9.3	8.9 [IQR: 7.4 - 9.5]<br>range: 3.7 - 11	p < 0.001	r = 0.45
	STARD3	8.8 [IQR: 8.6 - 9]<br>range: 7.7 - 9.4	9.3 [IQR: 9.1 - 9.4]<br>range: 8.4 - 9.8	p < 0.001	r = 0.58
	STARD3NL	10 [IQR: 10 - 11]<br>range: 8.8 - 11	9.8 [IQR: 9.5 - 10]<br>range: 9.1 - 11	p < 0.001	r = 0.54
	STARD4	6.7 [IQR: 6.1 - 7.1]<br>range: 4.9 - 7.8	7.2 [IQR: 6.8 - 7.6]<br>range: 5.8 - 8.3	p < 0.001	r = 0.44
	CYP11A1	5.9 [IQR: 5.1 - 6.9]<br>range: 2.6 - 11	7.6 [IQR: 6.8 - 8.8]<br>range: 4.6 - 12	p < 0.001	r = 0.52
	CYP17A1	5.2 [IQR: 4.1 - 6.6]<br>range: 3.2 - 12	3.9 [IQR: 3.2 - 6.6]<br>range: 3.2 - 13	p = 0.011	r = 0.22
	FDX1	7.9 [IQR: 7.6 - 8.3]<br>range: 6.6 - 9.6	8.3 [IQR: 8 - 8.6]<br>range: 7.3 - 9.3	p < 0.001	r = 0.33
	FDX2	8.7 [IQR: 8.5 - 9]<br>range: 8.1 - 9.8	8.5 [IQR: 8.3 - 8.8]<br>range: 7.9 - 9.6	p = 0.0033	r = 0.26
	FDXR	7.9 [IQR: 7.4 - 8.2]<br>range: 6.6 - 9.1	8.7 [IQR: 8.2 - 9.2]<br>range: 6.9 - 10	p < 0.001	r = 0.51

Cohort	Variable	Seminoma	NSGCT	Significance[a]	Effect size[a]
	HSD3B1	3 [IQR: 3 - 3]<br>range: 3 - 7	3.6 [IQR: 3 - 5.4]<br>range: 3 - 9.2	p < 0.001	r = 0.55
	HSD3B2	4.2 [IQR: 3.8 - 4.7]<br>range: 3.1 - 9.9	4.9 [IQR: 4 - 5.7]<br>range: 3.1 - 12	p = 0.0042	r = 0.25
	SERPINA6	3.3 [IQR: 3.3 - 3.8]<br>range: 3.3 - 5.9	4.7 [IQR: 3.6 - 6.9]<br>range: 3.3 - 8.9	p < 0.001	r = 0.47
	CYP21A2	5.2 [IQR: 4.6 - 5.9]<br>range: 3.3 - 7.7	6 [IQR: 5.3 - 6.5]<br>range: 4.2 - 8.1	p < 0.001	r = 0.37
	HSD11B1	7.8 [IQR: 6.9 - 8.9]<br>range: 3.5 - 11	6.6 [IQR: 5.9 - 7.6]<br>range: 3.3 - 9.7	p < 0.001	r = 0.35
	HSD11B2	5.1 [IQR: 4.8 - 5.6]<br>range: 3.9 - 7.6	7.9 [IQR: 7.6 - 8.3]<br>range: 4.2 - 9.2	p < 0.001	r = 0.82
	HSD17B1	5.5 [IQR: 5.3 - 5.9]<br>range: 3.9 - 7.2	6.5 [IQR: 6.1 - 6.9]<br>range: 4.4 - 8.8	p < 0.001	r = 0.69
	HSD17B2	2.9 [IQR: 2.9 - 3.3]<br>range: 2.9 - 4.1	6.5 [IQR: 3.8 - 8.1]<br>range: 2.9 - 11	p < 0.001	r = 0.69
	HSD17B11	9.1 [IQR: 8.8 - 9.5]<br>range: 7.9 - 10	9.3 [IQR: 9.1 - 9.7]<br>range: 7.5 - 11	p = 0.022	r = 0.2
	HSD17B12	10 [IQR: 10 - 10]<br>range: 9.4 - 12	11 [IQR: 11 - 11]<br>range: 10 - 12	p < 0.001	r = 0.56
	CYP19A1	3.7 [IQR: 3.2 - 4.3]<br>range: 2.5 - 6.6	4.6 [IQR: 3.8 - 5.7]<br>range: 2.5 - 8.6	p < 0.001	r = 0.38
	SRD5A1	7.1 [IQR: 6.9 - 7.4]<br>range: 5.9 - 8	7.8 [IQR: 7.6 - 8.1]<br>range: 6.4 - 8.8	p < 0.001	r = 0.65
	SRD5A2	4.4 [IQR: 3.8 - 4.8]<br>range: 3.4 - 5.4	4.6 [IQR: 4 - 5.7]<br>range: 3.4 - 8.7	p = 0.033	r = 0.18
	SRD5A3	8.5 [IQR: 8.1 - 8.9]<br>range: 7.4 - 10	7.2 [IQR: 6.9 - 7.5]<br>range: 6.4 - 9.7	p < 0.001	r = 0.69
	SHBG	4.4 [IQR: 4.1 - 4.8]<br>range: 3.6 - 6.4	6.6 [IQR: 5.9 - 7.5]<br>range: 4.3 - 9.7	p < 0.001	r = 0.77
GSE99420	Samples, N	30	30		
	GNRH1	6.1 [IQR: 5.9 - 6.2]<br>range: 4.8 - 7	6.3 [IQR: 6.2 - 6.8]<br>range: 5.4 - 7.7	p = 0.02	r = 0.39

Cohort	Variable	Seminoma	NSGCT	Significance[a]	Effect size[a]
	POMC	6.9 [IQR: 6.3 - 8]<br>range: 5.3 - 9.3	8.3 [IQR: 7.2 - 9.4]<br>range: 5.5 - 11	p = 0.022	r = 0.37
	FDX2	8.9 [IQR: 8.6 - 9]<br>range: 8 - 9.8	8.5 [IQR: 8.2 - 8.7]<br>range: 7.3 - 9.3	p = 0.011	r = 0.42
	HSD17B1	5.8 [IQR: 5.2 - 6.2]<br>range: 4.2 - 8	6.4 [IQR: 5.9 - 7]<br>range: 4.8 - 7.8	p = 0.022	r = 0.36
	HSD17B2	3.9 [IQR: 3.7 - 4.1]<br>range: 3.4 - 5.5	4.5 [IQR: 3.8 - 6.8]<br>range: 3.2 - 12	p = 0.022	r = 0.37
	SRD5A1	7.2 [IQR: 7 - 7.8]<br>range: 5.9 - 7.9	7.6 [IQR: 7.4 - 8]<br>range: 7 - 8.8	p = 0.0087	r = 0.44
	SRD5A3	8.1 [IQR: 7.7 - 8.8]<br>range: 6.8 - 10	7.1 [IQR: 7 - 7.9]<br>range: 6.7 - 8.9	p = 0.0064	r = 0.48
	SHBG	5 [IQR: 4.5 - 5.6]<br>range: 3 - 9.5	6.2 [IQR: 5.5 - 7.3]<br>range: 3.3 - 9.3	p = 0.008	r = 0.45

[a]Mann-Whitney test with r effect size statistic. P values adjusted for multiple testing with the false discovery rate method.

Supplementary Table S2: Expression of hormone-related genes in testicular cancers of the TCGA cohort stratified by ICD-O histological subtypes. Log2-transformed expression levels are presented as medians with interquartile ranges and ranges.

**Variable**	**SEM[a]**	**MGCT[a]**	**EMBCA[a]**	**TT[a]**	**TYST[a]**	**Significance [b]**	**Effect size[b]**
Samples, N	65	27	27	10	4		
GNRH1	6<br>[IQR: 5.8 - 6.4]<br>range: 5.2 - 7.4	6.4<br>[IQR: 6 - 6.6]<br>range: 5.1 - 7.1	6.3<br>[IQR: 5.9 - 6.7]<br>range: 5.4 - 7.3	6.5<br>[IQR: 6.2 - 6.8]<br>range: 6.1 - 7.1	5.9<br>[IQR: 5.9 - 6]<br>range: 5.8 - 6.1	$p = 0.033$	$\eta^2 = 0.051$
GNRH2	4.1<br>[IQR: 3.6 - 4.7]<br>range: 3.3 - 6.3	4.8<br>[IQR: 4.1 - 7]<br>range: 3.3 - 10	3.8<br>[IQR: 3.6 - 5.2]<br>range: 3.3 - 9.6	3.7<br>[IQR: 3.3 - 4.3]<br>range: 3.3 - 7.2	4.4<br>[IQR: 3.7 - 5.7]<br>range: 3.3 - 8	$p = 0.012$	$\eta^2 = 0.069$
PRL	3.1<br>[IQR: 3.1 - 3.4]<br>range: 3.1 - 4.6	4<br>[IQR: 3.5 - 5]<br>range: 3.1 - 7	3.7<br>[IQR: 3.5 - 4.3]<br>range: 3.1 - 5.8	5.7<br>[IQR: 5.4 - 7.2]<br>range: 3.6 - 9.6	3.3<br>[IQR: 3.1 - 3.5]<br>range: 3.1 - 3.5	$p < 0.001$	$\eta^2 = 0.43$
CGA	2.7<br>[IQR: 2.7 - 2.7]<br>range: 2.7 - 11	7<br>[IQR: 4.4 - 9.6]<br>range: 2.7 - 12	5.3<br>[IQR: 3.7 - 8.3]<br>range: 2.7 - 13	3.5<br>[IQR: 2.9 - 5.3]<br>range: 2.7 - 6.3	6.6<br>[IQR: 5.6 - 7.1]<br>range: 2.7 - 8.5	$p < 0.001$	$\eta^2 = 0.47$
LHB	5.4<br>[IQR: 5 - 5.9]<br>range: 3.4 - 7.8	4.4<br>[IQR: 4 - 5.3]<br>range: 3.4 - 6.5	4.4<br>[IQR: 4.1 - 5.1]<br>range: 3.4 - 7.3	4.1<br>[IQR: 3.6 - 4.4]<br>range: 3.4 - 4.8	4<br>[IQR: 3.7 - 4.3]<br>range: 3.4 - 4.7	$p < 0.001$	$\eta^2 = 0.31$
POMC	7<br>[IQR: 6.2 - 7.7]<br>range: 4.9 - 9.3	8.5<br>[IQR: 7.3 - 9]<br>range: 5.9 - 9.9	9.4<br>[IQR: 9.1 - 9.9]<br>range: 8.1 - 11	5.7<br>[IQR: 5.3 - 7.1]<br>range: 3.7 - 9.7	9.3<br>[IQR: 9.1 - 9.6]<br>range: 9 - 9.9	$p < 0.001$	$\eta^2 = 0.5$
STAR	6.5<br>[IQR: 6 - 7.7]<br>range: 4.5 - 11	6.8<br>[IQR: 5.7 - 7.4]<br>range: 3.4 - 12	7.9<br>[IQR: 7.4 - 9.3]<br>range: 5 - 12	5.7<br>[IQR: 5.2 - 6]<br>range: 4.1 - 7.5	6.9<br>[IQR: 6.2 - 7.8]<br>range: 4.7 - 10	$p < 0.001$	$\eta^2 = 0.2$
STARD3	8.8<br>[IQR: 8.7 - 9]<br>range: 7.7 - 9.4	9.1<br>[IQR: 8.9 - 9.3]<br>range: 8.4 - 9.8	9.3<br>[IQR: 9.2 - 9.4]<br>range: 8.9 - 9.8	9.3<br>[IQR: 8.9 - 9.5]<br>range: 8.6 - 9.7	9<br>[IQR: 8.9 - 9.2]<br>range: 8.7 - 9.5	$p < 0.001$	$\eta^2 = 0.31$
STARD3NL	10<br>[IQR: 10 - 11]<br>range: 8.8 - 11	9.9<br>[IQR: 9.7 - 10]<br>range: 9.3 - 11	9.8<br>[IQR: 9.6 - 10]<br>range: 9.2 - 10	9.8<br>[IQR: 9.6 - 9.9]<br>range: 9.1 - 10	9.9<br>[IQR: 9.6 - 10]<br>range: 9.3 - 10	$p < 0.001$	$\eta^2 = 0.25$
STARD4	6.7<br>[IQR: 6 - 7]<br>range: 4.9 - 7.8	7.3<br>[IQR: 6.8 - 7.7]<br>range: 6.2 - 8	7<br>[IQR: 6.6 - 7.5]<br>range: 5.9 - 8.2	7.6<br>[IQR: 7.3 - 7.8]<br>range: 6.9 - 8.3	7.2<br>[IQR: 6.9 - 7.3]<br>range: 6 - 7.5	$p < 0.001$	$\eta^2 = 0.25$
TSPO	9.4<br>[IQR: 8.8 - 9.9]	9.2<br>[IQR: 8.8 - 9.7]	9<br>[IQR: 8.7 - 9.6]	9.5<br>[IQR: 9.3 - 10]	8<br>[IQR: 7.9 - 8.5]	ns	$\eta^2 = 0.029$

Variable	SEM[a]	MGCT[a]	EMBCA[a]	TT[a]	TYST[a]	Significance [b]	Effect size[b]
	range: 6.6 - 12	range: 8.4 - 12	range: 7.8 - 11	range: 8.3 - 11	range: 7.9 - 9.3	(p = 0.1)	
TSPOAP1	7.1 [IQR: 6.7 - 7.6] range: 5.6 - 8.9	7.5 [IQR: 6.9 - 8.2] range: 5.5 - 9.4	6.5 [IQR: 6.2 - 7] range: 4.7 - 7.7	9.5 [IQR: 8.9 - 9.7] range: 8 - 10	8.3 [IQR: 7.4 - 9] range: 6.8 - 9.4	$p < 0.001$	$\eta^2 = 0.31$
CYP11A1	6 [IQR: 5.1 - 7] range: 2.6 - 11	7.4 [IQR: 6.8 - 8.4] range: 6.2 - 12	8 [IQR: 7.2 - 9.3] range: 5.4 - 11	7 [IQR: 6.6 - 7.4] range: 6.2 - 8	8.2 [IQR: 7.6 - 8.7] range: 6.3 - 9.6	$p < 0.001$	$\eta^2 = 0.28$
CYP17A1	5.3 [IQR: 4.1 - 7.1] range: 3.2 - 12	3.7 [IQR: 3.2 - 4.2] range: 3.2 - 13	5.5 [IQR: 3.5 - 8.4] range: 3.2 - 11	3.8 [IQR: 3.2 - 4.2] range: 3.2 - 8	3.8 [IQR: 3.6 - 5.2] range: 3.2 - 9.4	$p < 0.001$	$\eta^2 = 0.12$
FDX1	7.9 [IQR: 7.6 - 8.3] range: 6.6 - 9.6	8.2 [IQR: 8 - 8.5] range: 7.6 - 9.2	8.5 [IQR: 8.3 - 8.7] range: 7.9 - 9.3	7.9 [IQR: 7.8 - 8.2] range: 7.5 - 8.7	8.5 [IQR: 8.4 - 8.6] range: 8 - 8.9	$p < 0.001$	$\eta^2 = 0.17$
FDX2	8.7 [IQR: 8.5 - 9] range: 8.1 - 9.8	8.5 [IQR: 8.3 - 8.7] range: 8.1 - 9.2	8.6 [IQR: 8.3 - 8.8] range: 7.9 - 9.3	8.2 [IQR: 8.1 - 8.4] range: 7.9 - 8.9	8.4 [IQR: 8.2 - 8.6] range: 7.9 - 9.1	$p = 0.001$	$\eta^2 = 0.11$
FDXR	7.9 [IQR: 7.4 - 8.2] range: 6.6 - 9.1	8.3 [IQR: 8 - 9] range: 7.1 - 9.4	9.1 [IQR: 8.9 - 9.5] range: 8.3 - 10	7.5 [IQR: 7.3 - 8.1] range: 6.9 - 8.7	8.3 [IQR: 8.1 - 8.4] range: 7.4 - 8.7	$p < 0.001$	$\eta^2 = 0.43$
HSD3B1	3 [IQR: 3 - 3] range: 3 - 7	4.6 [IQR: 3.2 - 6] range: 3 - 8	3.5 [IQR: 3 - 5] range: 3 - 9.2	3.5 [IQR: 3 - 3.7] range: 3 - 5.2	3 [IQR: 3 - 3.8] range: 3 - 6.3	$p < 0.001$	$\eta^2 = 0.3$
HSD3B2	4.2 [IQR: 3.8 - 4.8] range: 3.1 - 9.9	4.4 [IQR: 4.2 - 5.3] range: 3.1 - 12	5.2 [IQR: 4 - 5.9] range: 3.1 - 7.7	4.9 [IQR: 4.5 - 5.1] range: 3.1 - 7.3	3.8 [IQR: 3.6 - 4.7] range: 3.6 - 6.8	ns ($p = 0.088$)	$\eta^2 = 0.032$
SERPINA6	3.3 [IQR: 3.3 - 3.8] range: 3.3 - 5.9	6 [IQR: 3.4 - 7.4] range: 3.3 - 8.3	3.7 [IQR: 3.3 - 4.9] range: 3.3 - 8.8	4.6 [IQR: 4.2 - 5.9] range: 3.3 - 7.9	7.1 [IQR: 5.8 - 7.5] range: 3.3 - 7.7	$p < 0.001$	$\eta^2 = 0.21$
CYP21A2	5.2 [IQR: 4.6 - 5.8] range: 3.3 - 7.7	5.7 [IQR: 5.2 - 6.3] range: 4.5 - 6.9	5.8 [IQR: 5.2 - 6.3] range: 4.2 - 7.7	6.7 [IQR: 6.4 - 6.9] range: 5.7 - 8	5.1 [IQR: 5 - 5.4] range: 4.9 - 6.1	$p < 0.001$	$\eta^2 = 0.17$
HSD11B1	7.7 [IQR: 6.9 - 8.8] range: 3.5 - 11	7.1 [IQR: 6.5 - 7.7] range: 3.3 - 8.5	6.8 [IQR: 6.2 - 7.9] range: 3.7 - 8.9	6.3 [IQR: 5.7 - 6.8] range: 4.2 - 9.7	4.4 [IQR: 4.2 - 5.1] range: 3.8 - 7.3	$p < 0.001$	$\eta^2 = 0.12$
HSD11B2	5.2 [IQR: 4.8 - 5.6]	8 [IQR: 7.7 - 8.3]	7.7 [IQR: 7.3 - 8]	8.2 [IQR: 8 - 8.5]	8.1 [IQR: 8 - 8.3]	$p < 0.001$	$\eta^2 = 0.71$

Variable	SEM[a]	MGCT[a]	EMBCA[a]	TT[a]	TYST[a]	Significance [b]	Effect size[b]
	range: 3.9 - 7.6	range: 5 - 9	range: 6.2 - 8.6	range: 7 - 8.8	range: 7.7 - 8.4		
HSD17B1	5.5<br>[IQR: 5.3 - 5.9]<br>range: 3.9 - 7.2	6.4<br>[IQR: 6.1 - 7]<br>range: 5.6 - 8.4	6.3<br>[IQR: 6 - 6.7]<br>range: 5.4 - 8.8	6.6<br>[IQR: 6.4 - 6.8]<br>range: 6.1 - 7	6.2<br>[IQR: 6 - 6.5]<br>range: 5.8 - 7	$p < 0.001$	$\eta^2 = 0.49$
HSD17B2	2.9<br>[IQR: 2.9 - 3.3]<br>range: 2.9 - 4.1	7.6<br>[IQR: 5.1 - 8.5]<br>range: 2.9 - 10	3.8<br>[IQR: 3.1 - 4.6]<br>range: 2.9 - 8.2	7.3<br>[IQR: 7.1 - 8]<br>range: 4.3 - 11	6.3<br>[IQR: 5.4 - 6.7]<br>range: 2.9 - 7.8	$p < 0.001$	$\eta^2 = 0.55$
HSD17B3	5.4<br>[IQR: 4.4 - 6.2]<br>range: 3.3 - 10	4.5<br>[IQR: 4.1 - 5.2]<br>range: 3.3 - 10	5.3<br>[IQR: 4.1 - 6.8]<br>range: 3.3 - 10	4.6<br>[IQR: 4.4 - 5.2]<br>range: 3.3 - 6.3	4.3<br>[IQR: 3.9 - 5.8]<br>range: 3.3 - 10	ns<br>($p = 0.26$)	$\eta^2 = 0.01$
HSD17B11	9.1<br>[IQR: 8.7 - 9.5]<br>range: 7.9 - 10	9.3<br>[IQR: 9 - 9.7]<br>range: 8.2 - 11	9.3<br>[IQR: 9.1 - 9.5]<br>range: 8.9 - 10	9.1<br>[IQR: 8.7 - 9.6]<br>range: 8.4 - 11	9.5<br>[IQR: 9.3 - 9.7]<br>range: 9.1 - 9.8	ns<br>($p = 0.11$)	$\eta^2 = 0.028$
HSD17B12	10<br>[IQR: 10 - 10]<br>range: 9.4 - 12	11<br>[IQR: 11 - 11]<br>range: 10 - 12	11<br>[IQR: 11 - 11]<br>range: 10 - 11	11<br>[IQR: 10 - 11]<br>range: 10 - 11	11<br>[IQR: 11 - 11]<br>range: 10 - 11	$p < 0.001$	$\eta^2 = 0.33$
HSD17B14	7.2<br>[IQR: 6.8 - 7.8]<br>range: 5.5 - 9.8	7.5<br>[IQR: 7.1 - 8.4]<br>range: 5.9 - 9.3	6.8<br>[IQR: 6.3 - 7.3]<br>range: 5.2 - 8.3	7.8<br>[IQR: 7.7 - 8.4]<br>range: 7 - 9	8.7<br>[IQR: 8.1 - 8.9]<br>range: 6.7 - 9.3	$p < 0.001$	$\eta^2 = 0.16$
CYP19A1	3.7<br>[IQR: 3.2 - 4.3]<br>range: 2.5 - 6.6	4.8<br>[IQR: 3.7 - 6.1]<br>range: 2.5 - 8.3	4.9<br>[IQR: 4.4 - 5.8]<br>range: 2.9 - 8.6	4.1<br>[IQR: 3.5 - 4.2]<br>range: 3.3 - 4.8	4.6<br>[IQR: 4.4 - 5.2]<br>range: 3.8 - 6.8	$p < 0.001$	$\eta^2 = 0.23$
SRD5A1	7.1<br>[IQR: 6.9 - 7.4]<br>range: 5.9 - 8	7.8<br>[IQR: 7.5 - 8]<br>range: 6.9 - 8.7	7.9<br>[IQR: 7.6 - 8.3]<br>range: 6.4 - 8.8	8<br>[IQR: 7.8 - 8.1]<br>range: 7.4 - 8.8	7.7<br>[IQR: 7.1 - 8.3]<br>range: 6.8 - 8.5	$p < 0.001$	$\eta^2 = 0.43$
SRD5A2	4.4<br>[IQR: 3.8 - 4.8]<br>range: 3.4 - 5.4	5.2<br>[IQR: 4.3 - 6.2]<br>range: 3.4 - 8.1	4.1<br>[IQR: 3.9 - 4.5]<br>range: 3.4 - 5.8	5.9<br>[IQR: 5 - 7.5]<br>range: 4.2 - 8.7	3.6<br>[IQR: 3.4 - 4.1]<br>range: 3.4 - 5	$p < 0.001$	$\eta^2 = 0.18$
SRD5A3	8.5<br>[IQR: 8.1 - 8.8]<br>range: 7.4 - 10	7.2<br>[IQR: 6.8 - 7.7]<br>range: 6.5 - 9.7	7.2<br>[IQR: 6.9 - 7.4]<br>range: 6.4 - 9	7<br>[IQR: 6.9 - 7.1]<br>range: 6.7 - 7.3	7.7<br>[IQR: 7.3 - 8.1]<br>range: 6.6 - 9.1	$p < 0.001$	$\eta^2 = 0.48$
SHBG	4.5<br>[IQR: 4.1 - 4.8]<br>range: 3.6 - 6.4	6.6<br>[IQR: 6.2 - 7.5]<br>range: 4.1 - 9.7	6.4<br>[IQR: 5.5 - 6.7]<br>range: 4.3 - 8.2	6.9<br>[IQR: 6.6 - 7.3]<br>range: 5.3 - 8.7	7.9<br>[IQR: 7 - 8.3]<br>range: 4.7 - 9.1	$p < 0.001$	$\eta^2 = 0.58$

[a]SEM: seminoma; MGCT: mixed germ cell tumor; EMBCA: embronal carcinoma; TT: teratoma and teratocarcinoma; TYST: yolk sac tumor.

Variable	SEM[a]	MGCT[a]	EMBCA[a]	TT[a]	TYST[a]	Significance[b]	Effect size[b]

[b]Kruskal-Wallis test with η^2 effect size statistic. P values corrected for multiple testing with the false discovery rate method.

Supplementary Table S3: Statistics for co-expression networks of hormone-related genes in in the TCGA and GSE99420 cohorts. Top genes with the largest degrees, betweenness, and hub scores in each cohort are presented. The complete table is available as a supplementary Excel file.

Cohort	Gene symbol	Gen classification	Degree	Betweenness	Hub score
TCGA	*PRL*	pituitary	13	0.028	0.66
	CGA	pituitary	16	0.011	0.89
	LHB	pituitary	2	0.12	3e-04
	POMC	pituitary	11	0.0019	0.57
	STAR	steroid	8	0.049	0.19
	STARD3	steroid	17	0.051	0.76
	TSPO	steroid	7	0.027	0.13
	CYP11A1	steroid	20	0.22	0.82
	FDX1	steroid	15	0.055	0.51
	FDX2	steroid	2	0.17	0.005
	FDXR	steroid	13	0.011	0.61
	HSD3B1	steroid	15	0.0019	0.86
	SERPINA6	steroid	14	0.025	0.71
	CYP21A2	adrenal	12	0.062	0.44
	HSD11B2	adrenal	16	0.0076	0.94
	HSD17B1	gonadal	17	0.0095	1
	HSD17B2	gonadal	18	0.078	0.88
	HSD17B3	gonadal	7	0.24	0.1
	HSD17B12	gonadal	15	0.034	0.62
	CYP19A1	gonadal	14	0.03	0.67
	SRD5A1	gonadal	13	0.028	0.65
	SRD5A3	gonadal	2	0.061	1.9e-05

Cohort	Gene symbol	Gen classification	Degree	Betweenness	Hub score
	SHBG	gonadal	18	0.0095	1
GSE99420	*GNRH1*	pituitary	3	0	0.0066
	GNRH2	pituitary	2	0.17	5.9e-06
	PRL	pituitary	1	0	0.018
	CGA	pituitary	1	0	0.0079
	POMC	pituitary	2	0	0.043
	STAR	steroid	5	0	0.98
	STARD3NL	steroid	3	0.051	0.2
	TSPOAP1	steroid	2	0.23	7e-04
	CYP11A1	steroid	8	0.076	1
	CYP17A1	steroid	5	0	0.97
	FDX2	steroid	3	0.047	7.5e-08
	HSD3B1	steroid	2	0.047	0.07
	HSD3B2	steroid	5	0	0.93
	HSD11B2	adrenal	6	0.32	0.053
	HSD17B1	gonadal	2	0.047	0.00073
	HSD17B2	gonadal	5	0.32	0.0068
	HSD17B3	gonadal	6	0.023	0.94
	HSD17B12	gonadal	5	0.12	0.14
	HSD17B14	gonadal	2	0.2	6.7e-05
	CYP19A1	gonadal	7	0.21	0.79
	SRD5A1	gonadal	3	0	0.0069
	SRD5A2	gonadal	3	0	0.038
	SRD5A3	gonadal	3	0.13	5.4e-07
	SHBG	gonadal	6	0.2	0.22

Supplementary Table S4: Metrics of cluster separation, potential misclassification, explained variance, and neighborhood misclassification for the hormonal clusters in the TCGA training cohort and the GSE99420 test collective.

Cohort	Silhouette width	Misclassification rate[a]	Explained clustering variance[b]	Neighborhood misclassification[c]
GSE99420	0.33	0.027	0.51	0.11
TCGA	0.18	0.170	0.34	0.32

[a]Fraction of observations with negative silhouette widths.

[b]Ratio of the total between sum of squares to the total sum of squares.

[c]Mean fraction of the five nearest neighbors assigned to different clusters.

Supplementary Table S5: Expression of the hormone-related cluster-defining genes in the hormonal clusters of testicular cancer samples in the training TCGA cohort and the GSE99420 test collective. Statistical significance of differences between the clusters was determined by Kruskal-Wallis test with with eta-square effect size statistic. P values were corrected for multiple testing with the false discovery rate method. Log2-transformed expression levels are shown as medians with interquartile ranges and ranges. The table is available as a supplementary Excel file.

Supplementary Table S6: Clinical characteristic of the hormonal clusters in the TCGA and GSE99420 cohorts. Quantitative variables are presented as medians with interquartile ranges and ranges. Qualitative variables are presented as percentages and counts of the categories within the clusters.

**Cohort**	**Variable**[a]	**#1**	**#2**	**#3**	**#4**	**Significance**[b]	**Effect size**[b]
TCGA	Age	28<br>[IQR: 23 - 32]<br>range: 20 - 53<br>complete: n = 16	33<br>[IQR: 28 - 38]<br>range: 20 - 52<br>complete: n = 62	29<br>[IQR: 25 - 36]<br>range: 20 - 66<br>complete: n = 23	28<br>[IQR: 23 - 34]<br>range: 14 - 67<br>complete: n = 32	p = 0.031	η^2 = 0.048
	Race/Ethnicity	Asian: 6.7% (1)<br>Black or African American: 0% (0)<br>White: 93% (14)<br>complete: n = 15	Asian: 5.1% (3)<br>Black or African American: 5.1% (3)<br>White: 90% (53)<br>complete: n = 59	Asian: 0% (0)<br>Black or African American: 9.1% (2)<br>White: 91% (20)<br>complete: n = 22	Asian: 0% (0)<br>Black or African American: 3.1% (1)<br>White: 97% (31)<br>complete: n = 32	ns<br>(p = 0.55)	V = 0.14
	Tumor stage	I: 80% (12)<br>II: 13% (2)<br>III: 6.7% (1)<br>complete: n = 15	I: 96% (54)<br>II: 1.8% (1)<br>III: 1.8% (1)<br>complete: n = 56	I: 80% (16)<br>II: 5% (1)<br>III: 15% (3)<br>complete: n = 20	I: 65% (20)<br>II: 23% (7)<br>III: 13% (4)<br>complete: n = 31	p = 0.013	V = 0.27
	Metastasis stage	M0: 93% (14)<br>M1: 6.7% (1)<br>complete: n = 15	M0: 100% (53)<br>M1: 0% (0)<br>complete: n = 53	M0: 89% (16)<br>M1: 11% (2)<br>complete: n = 18	M0: 97% (31)<br>M1: 3.1% (1)<br>complete: n = 32	ns<br>(p = 0.16)	V = 0.22
	Node stage	N0: 75% (6)<br>N1: 25% (2)<br>N2: 0% (0)<br>complete: n = 8	N0: 90% (18)<br>N1: 5% (1)<br>N2: 5% (1)<br>complete: n = 20	N0: 91% (10)<br>N1: 9.1% (1)<br>N2: 0% (0)<br>complete: n = 11	N0: 63% (12)<br>N1: 32% (6)<br>N2: 5.3% (1)<br>complete: n = 19	ns<br>(p = 0.38)	V = 0.24
	IGCCCG	good: 60% (3)<br>intermediate: 40% (2)<br>poor: 0% (0)<br>complete: n = 5	good: 86% (12)<br>intermediate: 14% (2)<br>poor: 0% (0)<br>complete: n = 14	good: 50% (4)<br>intermediate: 25% (2)<br>poor: 25% (2)<br>complete: n = 8	good: 81% (13)<br>intermediate: 19% (3)<br>poor: 0% (0)<br>complete: n = 16	ns<br>(p = 0.12)	V = 0.36
	Histology	seminoma: 0% (0)<br>NSGCT: 100% (24)<br>complete: n = 24	seminoma: 97% (57)<br>NSGCT: 3.4% (2)<br>complete: n = 59	seminoma: 3.8% (1)<br>NSGCT: 96% (25)<br>complete: n = 26	seminoma: 11% (4)<br>NSGCT: 89% (31)<br>complete: n = 35	p < 0.001	V = 0.9
	Histology, ICD-O	SEM: 0% (0)<br>MGCT: 44% (7)<br>EMBCA: 0% (0)<br>TT: 56% (9)	SEM: 97% (60)<br>MGCT: 1.6% (1)<br>EMBCA: 1.6% (1)<br>TT: 0% (0)	SEM: 4.3% (1)<br>MGCT: 57% (13)<br>EMBCA: 26% (6)<br>TT: 4.3% (1)	SEM: 12% (4)<br>MGCT: 19% (6)<br>EMBCA: 62% (20)	p < 0.001	V = 0.69

Cohort	Variable[a]	#1	#2	#3	#4	Significance[b]	Effect size[b]
		TYST: 0% (0)<br>complete: n = 16	TYST: 0% (0)<br>complete: n = 62	TYST: 8.7% (2)<br>complete: n = 23	TT: 0% (0)<br>TYST: 6.2% (2)<br>complete: n = 32		
	Marker status	S0: 12% (2)<br>S1: 56% (9)<br>S2: 31% (5)<br>S3: 0% (0)<br>complete: n = 16	S0: 57% (29)<br>S1: 27% (14)<br>S2: 9.8% (5)<br>S3: 5.9% (3)<br>complete: n = 51	S0: 8.7% (2)<br>S1: 13% (3)<br>S2: 70% (16)<br>S3: 8.7% (2)<br>complete: n = 23	S0: 34% (10)<br>S1: 38% (11)<br>S2: 28% (8)<br>S3: 0% (0)<br>complete: n = 29	$p < 0.001$	V = 0.35
	Radiation	0% (0)<br>complete: n = 16	35% (21)<br>complete: n = 60	0% (0)<br>complete: n = 23	0% (0)<br>complete: n = 31	$p < 0.001$	V = 0.47
GSE99420	Histology	seminoma: 14% (1)<br>NSGCT: 86% (6)<br>complete: n = 7	seminoma: 73% (22)<br>NSGCT: 27% (8)<br>complete: n = 30	seminoma: 0% (0)<br>NSGCT: 100% (5)<br>complete: n = 5	seminoma: 39% (7)<br>NSGCT: 61% (11)<br>complete: n = 18	$p = 0.0011$	V = 0.52

[a]IGCCCG: International Germ Cell Cancer Collaborative Group risk strata; ICD-0: international classification of diseases for oncology, histological subtype; SEM: seminoma; MGCT: mixed germ cell tumor; EMBCA: embronal carcinoma; TT: teratoma and teratocarcinoma; TYST: yolk sac tumor.

[b]Numeric variables: Kruskal-Wallis test with η^2 effect size statistic. Categorical variables: χ^2 test with Cramer's V effect size statistic. P values corrected for multiple testing with the false discovery rate method.

Supplementary Table S7: Numeric statistics of performance of RIDGE Cox proportional hazard models of progression-free survival in the TCGA cohort.

**Model type**[a]	**Progression cases, N**	**Observations, N**	**C-index**[b]	**R-square**[c]	**IBS**
Cluster-only PFS model	33	119	0.64<br>[95% CI: 0.55 to 0.72]	0.1	0.21
Clinical PFS model	33	119	0.67<br>[95% CI: 0.57 to 0.76]	0.14	0.22
Cluster/clinical PFS model	33	119	0.65<br>[95% CI: 0.56 to 0.75]	0.15	0.21

[a]Clinical factors: age, serum marker stage, and histological subtype.

[b]C-index: Harrell's concordance index with 95% confidence interval.

[c]IBS: integrated Brier score.

Supplementary Table S8: Non-malignant cell content in the hormonal clusters of the TCGA and GSE99420 cohorts was estimated by the QuanTIseq, xCell, and MCP Counter algorithms. Differences in the predicted cell levels the clusters were investigated by Kruskal-Wallis test with eta-square effect size statistic. P values were corrected for multiple tesing with the false discovery rate method. Median infiltration levels with interquartile ranges and ranges are shown. The table is available as a supplementary Excel file.

Supplementary Table S9: Differences in single sample gene set enrichment analysis scores (ssGSEA scores) of the Reactome pathway gene signatures were compared between the hormonal clusters by one-way ANOVA with eta-square effect size statistic. P values were corrected for multiple testing with the false discovery rate method. Median ssGSEA scores with interquartile ranges and ranges are shown for gene signatures found to differ significantly between the clusters in both the TCGA and GSE99420 cohorts. The table is available as a supplementary Excel file.

Supplementary Table S10: Genes differentially regulated in the hormonal clusters as compared with the cohort means. Statistical significance of differences in log2-transformed expression levels between the clusters was assessed by one-way ANOVA with eta-square effect size statistic. Statistical significance of differences between log2-transformed expression in the cluster and the cohort mean was determined by one-sample two-tailed T test P values were corrected for multiple testing with the false discovery rate method. Differential regulation in a particular cluster was considered for pFDR(ANOVA) < 0.05, eta-squared of at least 0.14, and pFDR(T test) < 0.05. The table is available as a supplementary Excel file.

Supplementary Table S11: Biological process gene ontology (GO) term enrichment analysis for genes found differentially regulated in the hormonal clusters was performed with the goana algorithm. Enrichment p values were corrected for multiple testing with the false discovery rate method (FDR). Odds ratio of enrichment in the differentially regulated gene set as compared with the entire genome served as enrichment effect size metric. GO terms found to be significantly enriched in the corresponding clusters of both the TCGA and GSE99420 cohort are shown. The table is available as a supplementary Excel file.

Supplementary Table S12: Differential modulation of transcriptional collecTRI regulons in the hormonal clusters as compared with the cohort mean. Analysis of regulon activity was investigated by univariable linear modeling algorithm from the decoupleR package fed with T statistic values of differential gene expression of all available genes. Magnitude of differential regulon activity was measured by linear modeling score (LM score). P values of non-zero LM score were corrected for multiple testing with the false discovery rate method. Regulons found

to be differentially modulated in both the TCGA and GSE99420 cohorts are shown. The table is available as a supplementary Excel file.

Supplementary Table S13: Differential regulation of PROGENy signaling pathways in the hormonal clusters as compared with the cohort mean. Analysis of signaling pathway activity was investigated by multivariable linear modeling algorithm from the decoupleR package fed with T statistic values of differential gene expression of all available genes. Magnitude of differential pathway activity was measured by linear modeling score (LM score). P values of non-zero LM score were corrected for multiple testing with the false discovery rate method. Signaling pathways found to be differentially regulated in both the TCGA and GSE99420 cohorts are shown.

Cluster	Signaling pathway	Cohort	Status vs cohort mean	LM score	Significance
#1	Hypoxia	GSE99420	activated	5.5	p < 0.001
		TCGA	activated	7.6	p < 0.001
	JAK-STAT	GSE99420	inhibited	-3.0	p = 0.0082
		TCGA	inhibited	-4.0	p < 0.001
	MAPK	GSE99420	inhibited	-4.1	p < 0.001
		TCGA	inhibited	-10.0	p < 0.001
	TGFb	GSE99420	activated	5.2	p < 0.001
		TCGA	activated	6.5	p < 0.001
	VEGF	GSE99420	inhibited	-3.6	p = 0.001
		TCGA	inhibited	-4.1	p < 0.001
#2	EGFR	GSE99420	inhibited	-4.8	p < 0.001
		TCGA	inhibited	-5.8	p < 0.001
	Hypoxia	GSE99420	inhibited	-5.4	p < 0.001
		TCGA	inhibited	-3.4	p = 0.0016
	JAK-STAT	GSE99420	activated	4.9	p < 0.001
		TCGA	activated	5.2	p < 0.001
	TGFb	GSE99420	inhibited	-4.4	p < 0.001
		TCGA	inhibited	-4.8	p < 0.001
	p53	GSE99420	inhibited	-5.4	p < 0.001

Cluster	Signaling pathway	Cohort	Status vs cohort mean	LM score	Significance
		TCGA	inhibited	-5.5	p < 0.001
#3	EGFR	GSE99420	activated	4.6	p < 0.001
		TCGA	activated	6.7	p < 0.001
	Hypoxia	GSE99420	activated	4.6	p < 0.001
		TCGA	activated	5.4	p < 0.001
	TGFb	GSE99420	activated	7.6	p < 0.001
		TCGA	activated	5.9	p < 0.001
	WNT	GSE99420	activated	4.4	p < 0.001
		TCGA	activated	3.7	p < 0.001
#4	EGFR	GSE99420	activated	3.4	p = 0.0053
		TCGA	activated	3.7	p < 0.001
	Estrogen	GSE99420	activated	4.7	p < 0.001
		TCGA	activated	3.2	p = 0.0032

Supplementary Table S14: Differential regulation of RECON2 model reactions in the hormonal clusters as compared with the cohort mean was investigated by Monte Carlo simulation fed with log2 fold-regulation estimates of differential expression and their standard errors for all available genes. Log2 fold-regulation estimates of reaction activity with 95% confidence intervals (95% CI) and false discovery rate (FDR) corrected p values are presented for metabolic reactions found to be significantly regulated both in the TCGA and GSE99420 cohort. The table is available as a supplementary Excel file.

Supplementary Table S15: Enrichment of RECON metabolic subsystems with significantly activated and inhibited reactions in the hormonal clusters of testicular cancer. Statistical significance was investigated by comparing frequency of the subsystem reaction within the activated or inhibited reaction set with 100000 random draws from the total reaction pool. Odds ratio (OR) of enrichment in the regulated reaction set over the entire reaction pool served as an effect size metric. Because p values decrease with increasing numbers or random draws, no multiple testing correction was applied. Metabolic subsystems found significantly enriched with activated or inhibited reactions in both the TCGA and GSE99420 cohorts are presented. The table is available as a supplementary Excel file.

Supplementary Table S16: Expression of proteins in the hormonal clusters of the TCGA cohorts was compared with the cohort means. Statistical significance of differences in log2-transformed expression levels between the clusters was assessed by one-way ANOVA with eta-square effect size statistic. Statistical significance of differences between log2-transformed expression in the cluster and the cohort mean was determined by one-sample two-tailed T test. P values were corrected for multiple testing with the false discovery rate method. Differential regulation in a particular cluster was considered for pFDR(ANOVA) < 0.05, eta-squared of at least 0.14, and pFDR(T test) < 0.05. The table is available as a supplementary Excel file.

Supplementary Table S17: Total mutation numbers, scores of microsatellite instability, and numbers of gene mutations deletions, and amplifications in the hormonal clusters of the TCGA cohort. Medians with interquartile ranges and ranges are shown.

**Variable**[a]	**#1**	**#2**	**#3**	**#4**	**Significance**[b]	**Effect size**[b]
Samples, N	24	63	27	35		
Total mutation burden, alterations per MB	0.48 [IQR: 0.3 - 0.68] range: 0.17 - 1.2	0.4 [IQR: 0.27 - 0.57] range: 0 - 1.3	0.4 [IQR: 0.32 - 0.63] range: 0 - 1.4	0.4 [IQR: 0.32 - 0.7] range: 0.033 - 1.9	ns (p = 0.6)	η^2 = -0.0047
MANTIS MSI score	0.33 [IQR: 0.32 - 0.34] range: 0.3 - 0.35	0.32 [IQR: 0.31 - 0.33] range: 0.29 - 0.34	0.32 [IQR: 0.32 - 0.33] range: 0.31 - 0.35	0.33 [IQR: 0.32 - 0.33] range: 0.31 - 0.34	p = 0.0072	η^2 = 0.073
SENSOR MSI score	0.28 [IQR: 0.21 - 0.4] range: 0 - 1.1	0.02 [IQR: 0 - 0.16] range: 0 - 0.63	0.29 [IQR: 0.085 - 0.54] range: 0 - 1.6	0.1 [IQR: 0.015 - 0.3] range: 0 - 1.1	p < 0.001	η^2 = 0.21
Mutation number	14 [IQR: 9.8 - 20] range: 5 - 35	12 [IQR: 8.5 - 17] range: 0 - 38	13 [IQR: 9.5 - 18] range: 0 - 42	12 [IQR: 10 - 20] range: 2 - 58	ns (p = 0.6)	η^2 = -0.004
Gene deletion number	7 [IQR: 2.5 - 68] range: 0 - 530	1 [IQR: 0 - 6] range: 0 - 620	19 [IQR: 2.5 - 64] range: 0 - 510	8 [IQR: 3.5 - 42] range: 0 - 750	p < 0.001	η^2 = 0.12
Gene amplificatio n number	0 [IQR: 0 - 26] range: 0 - 280	0 [IQR: 0 - 48] range: 0 - 1100	0 [IQR: 0 - 24] range: 0 - 560	0 [IQR: 0 - 70] range: 0 - 470	ns (p = 0.6)	η^2 = -0.0077

[a]MB: million base pairs; MSI: microsatellite instability.

[b]Kruskal-Wallis test with η^2 effect size statistic. P values corrected for multiple testing with the false discovery rate method.

Supplementary Table S18: Frequencies of gene mutations, deletions, and amplifications in the hormonal clusters of the TCGA cohort. Statistical significance of differences between the clusters was determined by chi-square test with Cramer's V effect size statistic. P values were corrected for multiple testing with the false discovery rate method. The table is available as a supplementary Excel file.

Supplementary Table S19: Drug response in form of log IC50 and area under response curve (AUC) was predicted for cancer samples by whole-transcriptome RIDGE linear models trained with the CTRP2 and GDSC drug screening data sets. Statistical significance of differences in the drug response metrics between the clusters was assessed by one-way ANOVA with eta-square effect size statistic. Statistical significance of differences between the drug response metrics in the cluster and the cohort mean was determined by one-sample two-tailed T test P values were corrected for multiple testing with the false discovery rate method. Differential drug response in a particular cluster was considered for pFDR(ANOVA) < 0.05, eta-squared of at least 0.14, and pFDR(T test) < 0.05. The table is available as a supplementary Excel file.

Supplementary Figures

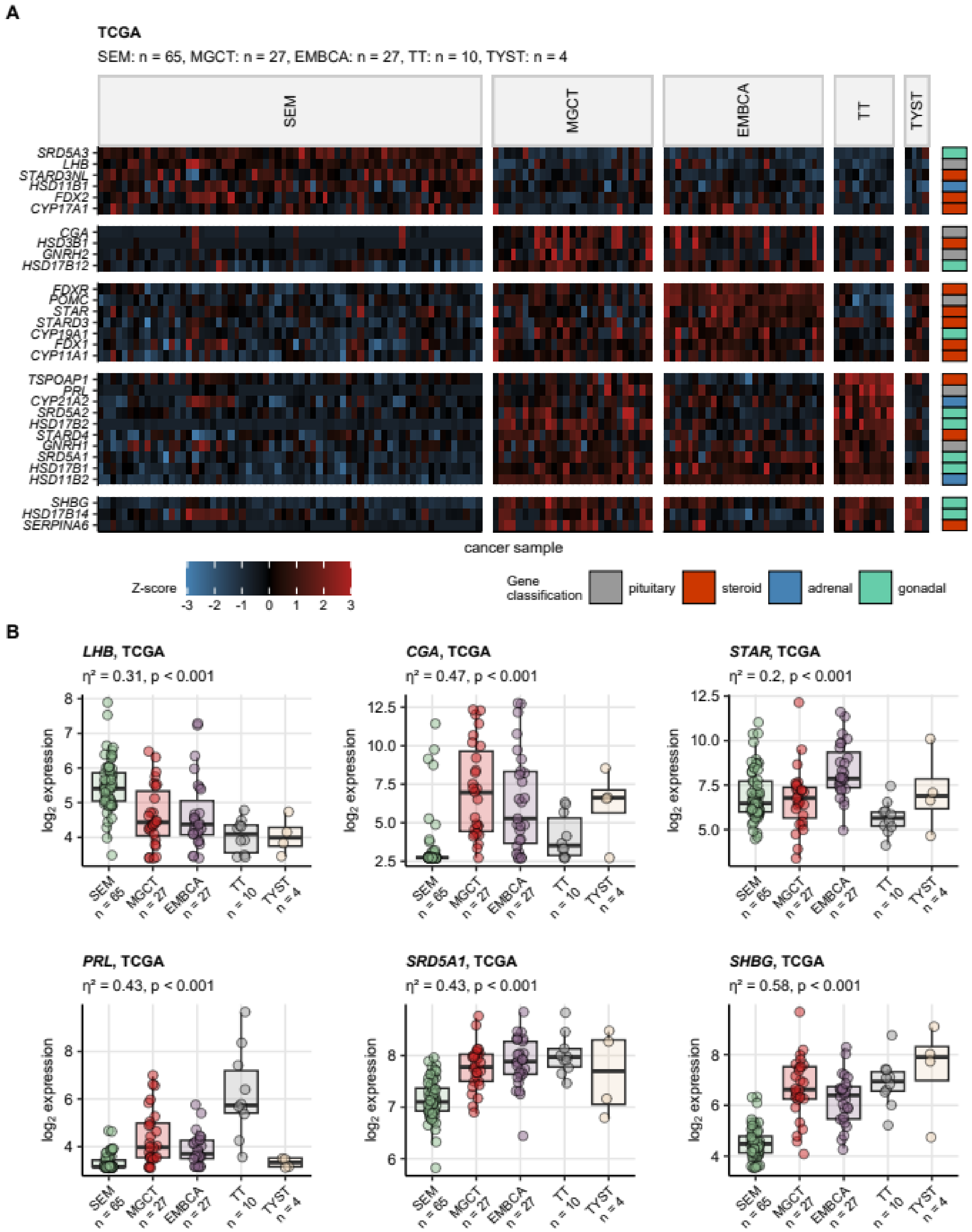
A
TCGA
SEM: n = 65, MGCT: n = 27, EMBCA: n = 27, TT: n = 10, TYST: n = 4
SEM
MGCT
EMBCA
TT
TYST
SRD5A3
LHB
STARD3NL
HSD11B1
FDX2
CYP17A1
CGA
HSD3B1
GNRH2
HSD17B12
FDXR
POMC
STAR
STARD3
CYP19A1
FDX1
CYP11A1
TSPOAP1
PRL
CYP21A2
SRD5A2
HSD17B2
STARD4
GNRH1
SRD5A1
HSD17B1
HSD11B2
SHBG
HSD17B14
SERPINA6
cancer sample
Z-score
-3 -2 -1 0 1 2 3
Gene classification
pituitary
steroid
adrenal
gonadal
B
LHB, TCGA
η² = 0.31, p < 0.001
CGA, TCGA
η² = 0.47, p < 0.001
STAR, TCGA
η² = 0.2, p < 0.001
PRL, TCGA
η² = 0.43, p < 0.001
SRD5A1, TCGA
η² = 0.43, p < 0.001
SHBG, TCGA
η² = 0.58, p < 0.001
log2 expression
SEM n = 65
MGCT n = 27
EMBCA n = 27
TT n = 10
TYST n = 4

Supplementary Figure S1. Differential expression of hormone-related genes in histological subtypes of testicular cancer in the TCGA cohort.

Detailed histological subtyping according to ICD-O (international classification of diseases for oncology) was available for the TCGA cancer samples. log_2*-transformed cancer tissue expression of 34 sex hormonal-related genes was compared between the histological subtypes by Kruskal-Wallis test with* η^2 *effect size statistic. P values were corrected for multiple testing with the false discovery rate method.*

(A) *Normalized* log_2 *expression levels (Z-scores) of significantly regulated genes (pFDR < 0.05) in the histological subtypes are presented in a heat map. The genes are arranged by their peak expression in the histological subsets. Functional gene classification is color coded in the vertical rug plot.*

(B) log_2 *expression levels for selected, strongly regulated hormone-related genes characteristic for particular histological subtypes. Median* log_2 *expression levels with interquartile ranges are presented in boxes with whiskers spanning over 150% of the interquartile ranges. Single cancer samples are visualized as points. Effect sizes and p values are displayed in the plot captions. Numbers of samples assigned to the histological subtypes are indicated in the X axis.*

SEM: seminoma; MGCT: mixed germ cell tumor; EMBCA: embryonal carcinoma; TT: teratoma and teratocarcinoma; TYST: yolk sac tumor.

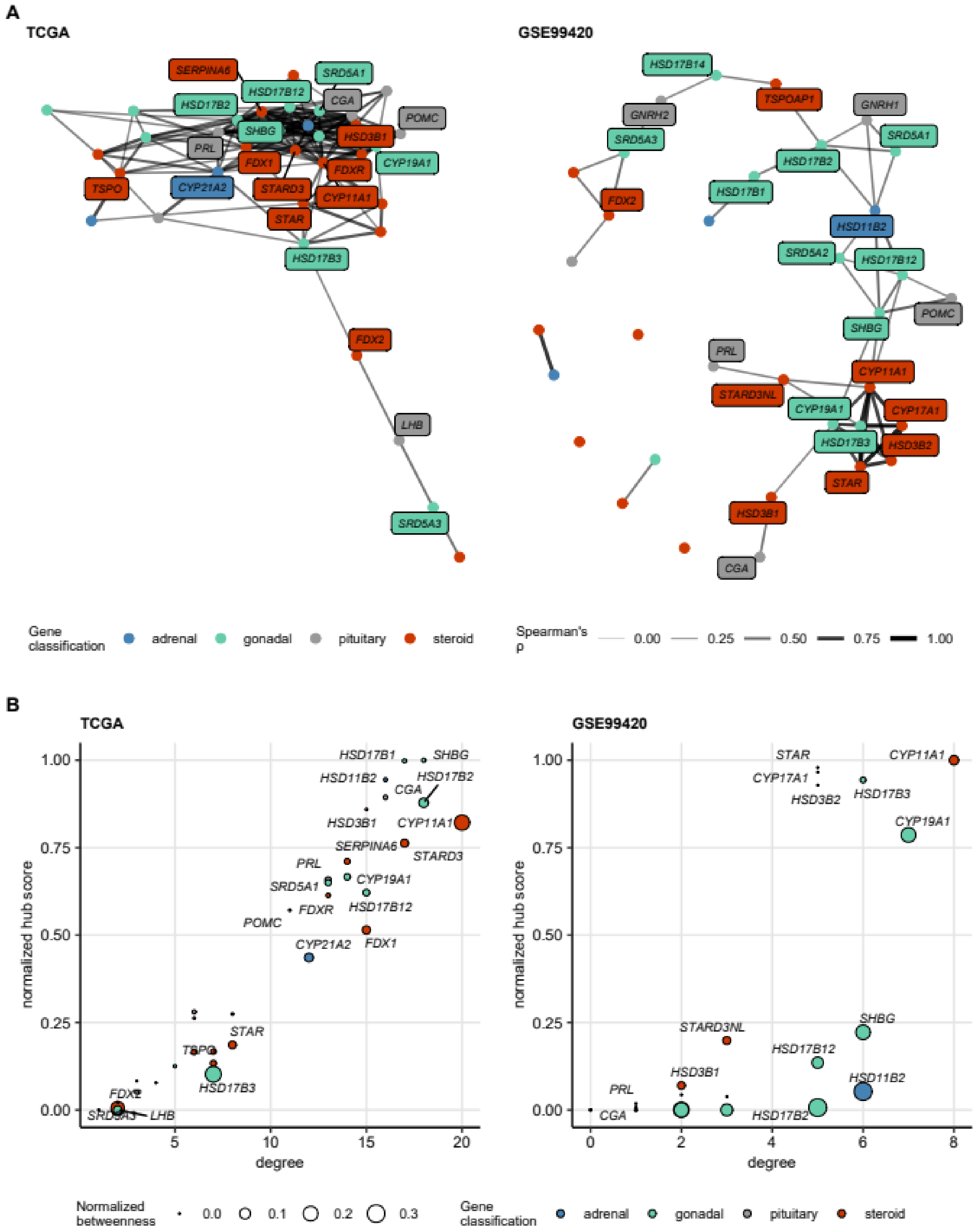
A
TCGA
GSE99420
Gene classification
adrenal
gonadal
pituitary
steroid
Spearman's ρ
0.00
0.25
0.50
0.75
1.00
B
TCGA
GSE99420
normalized hub score
degree
Normalized betweenness
0.0
0.1
0.2
0.3

Supplementary Figure S2. Networks of co-expressed hormone-related genes in testicular carcinoma.

Pairwise correlation of cancer tissue expression of 34 sex hormone-related genes was investigated by Spearman's ρ correlation coefficients. Associations with $\rho \geq 0.3$ *were analyzed and visualized as undirected graphs.*

(A) *Visualization of the gene co-expression graph in the TCGA and GSE99420 cohort. Graph nodes are depicted as points, whose color codes for functional gene classification. Edge color and width codes for Spearman's ρ value. Note the clusters of co-expressed genes involved in general steroid biosynthesis (STAR, CYP11A1, HSD3B1/2, CYP17A1), and gonadal hormone production and transport (CYP19A1, HSD17B3, SHBG).*

(B) *Graph node importance statistics for the hormone-related genes. Degree represents the numbers of node neighbors. Betweenness is defined as the number of shortest paths between node pairs passing through the particular node divided by the total node pair number. Hub scores are defined as the principal eigenvector of the matrix of Spearman's ρ coefficients. Degree and betweenness for the sex hormone-related genes are presented in the scatter plot axes. Point size codes for hub score value. Functional classification of the genes is color-coded.*

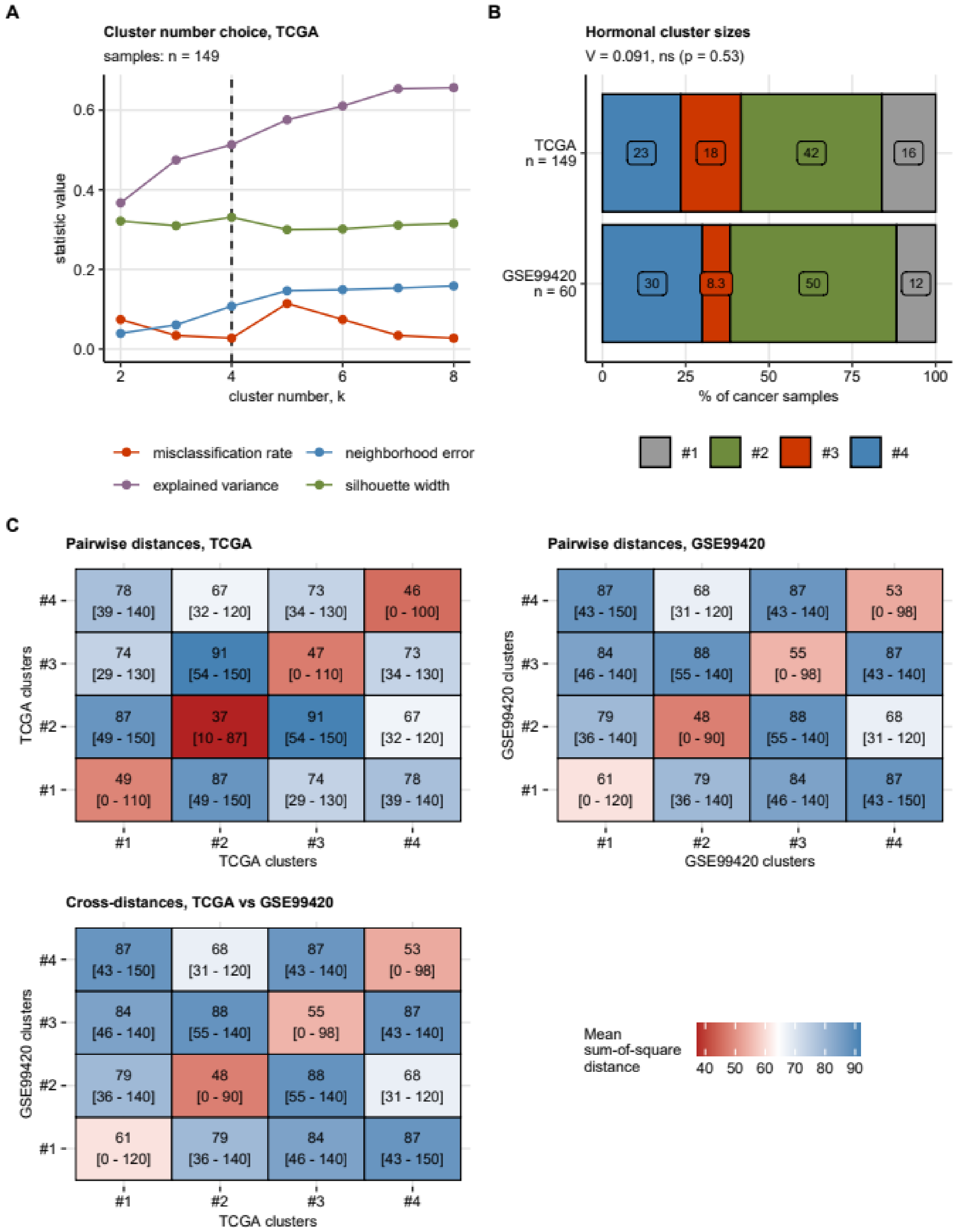
A
Cluster number choice, TCGA
samples: n = 149
statistic value
cluster number, k
misclassification rate
neighborhood error
explained variance
silhouette width
B
Hormonal cluster sizes
V = 0.091, ns (p = 0.53)
TCGA
n = 149
GSE99420
n = 60
23
18
42
16
30
8.3
50
12
% of cancer samples
#1
#2
#3
#4
C
Pairwise distances, TCGA
TCGA clusters
Pairwise distances, GSE99420
GSE99420 clusters
Cross-distances, TCGA vs GSE99420
GSE99420 clusters
TCGA clusters
Mean
sum-of-square
distance

Supplementary Figure S3. Development of the hormonal clusters with the TCGA training data set and evaluation of the clustering structures in the TCGA and GSE99420 cohorts.

Testicular cancer samples in the TCGA training cohort were assigned to the hormonal clusters in respect to ComBat-adjusted log_2 *expression values of 34 sex hormone-related genes by hard-threshold regularized KMEANS unsupervised clustering. The hormonal cluster assignment was predicted for cancer samples in the GSE99420 training collective by a Random Forest classifier fed with ComBat-adjusted* log_2*-transformed expression levels of the cluster-defining genes.*

(A) *Choice of cluster number k = 4 in the unsupervised clustering of the TCGA training cohort samples was motivated by the maximum of mean silhouette width. Statistics of cluster separation (mean silhouette width), misclassification rate (fraction of observations with negative silhouette widths), explained variance (ratio of the total between-cluster sum of squares to the total sum of squares), and neighborhood error (mean fraction of the five nearest neighbors assigned to different clusters) for various k values are presented.*

(B) *Comparison of sizes of the hormonal clusters in the training TCGA cohort and the test GSE99420 collective. Statistical significance was determined by* χ^2 *test with Cramer's V effect size statistic. Percentages of samples in the hormonal clusters are shown in a stack plot. The effect size and p value are displayed in the plot caption. Numbers of cancer samples in the cohorts are indicated in the Y axis.*

(C) *Similarity and separation of the hormonal clusters was investigated by comparing pairwise sum-of-square distances between observations in each of the TCGA and GSE99420 cohort. Similarity of the clusters in the training TCGA cohort and the test GSE99420 cohort was assessed by comparing sum-of-square cross-distances (i.e. each of observations in the GSE99420 cohort with all observations in the TCGA cohort). Mean pairwise distances and cross-distances are presented in heat maps. The heat map tiles are labeled with mean distances with 95 percentile ranges.*

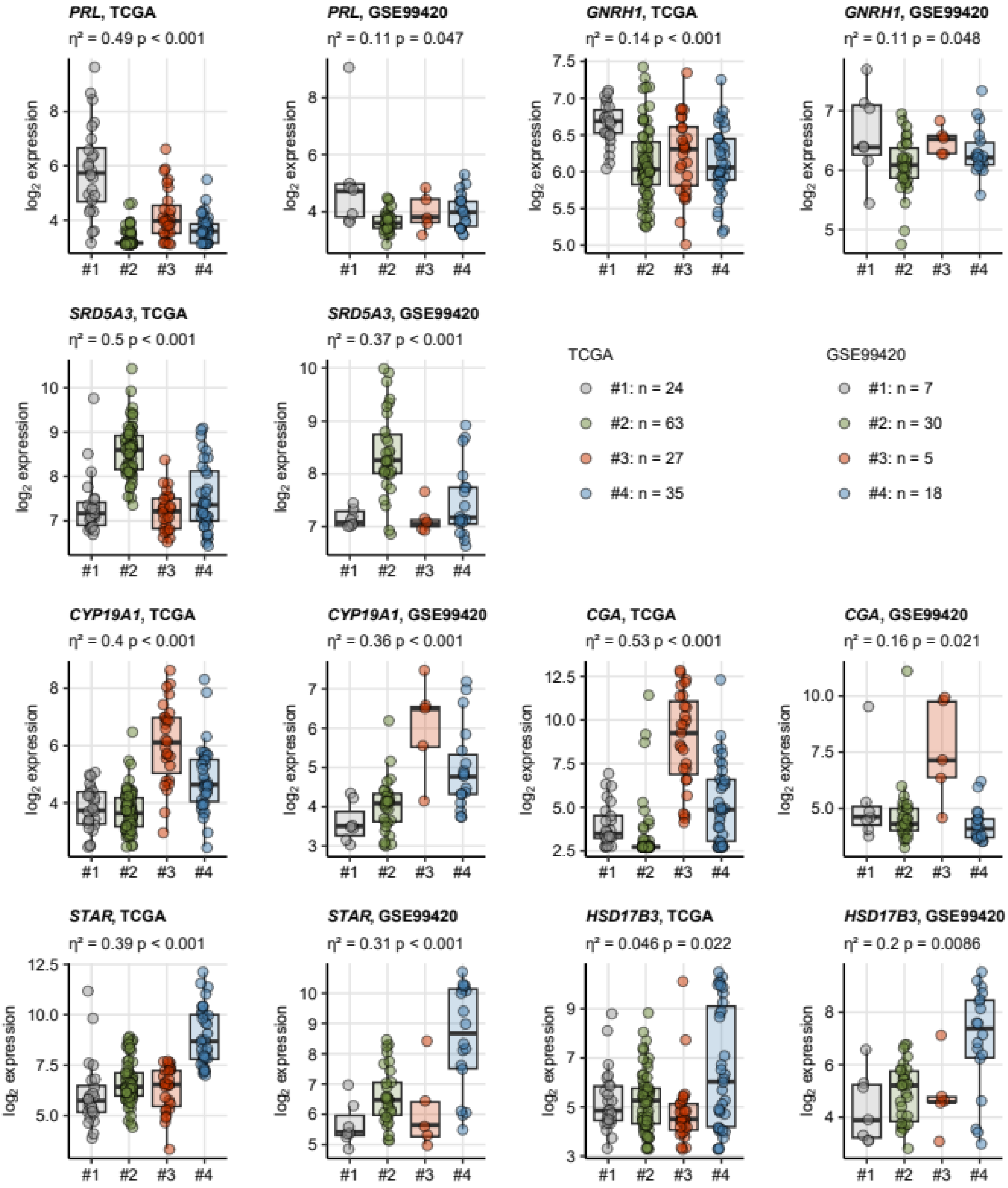

Supplementary Figure S4. Expression of the key cluster-defining hormone-related genes.

log_2 expression of the hormonal cluster-defining genes was compared between the hormonal clusters by Kruskal-Wallis test with η^2 effect size statistic. P values were adjusted for multiple testing with the false discovery rate method. Expression of PRL (prolactin, maximum in cluster #1), GNRH1 (gonadotropin releasing hormone 1, cluster #1), SRD5A3 (steroid 5 alpha-reductase 3, testosterone catabolism, #2), CYP19A1 (aromatase, synthesis of estradiol, cluster #3), CGA (glycoprotein hormones, alpha polypeptide or gonadotropins, cluster #3), STAR (steroidogenic acute regulatory protein, steroid synthesis, cluster #4), and HSD17B3 (hydroxysteroid 17-beta dehydrogenase 3, testosterone synthesis, cluster #4) was found to differ strongly between the hormonal clusters in both the TCGA and GSE99420 cohorts. Median log_2 expression values with interquartile ranges are depicted as boxes with whiskers spanning over 150% of the interquartile ranges. Single cancer samples are visualized as points. Effect sizes and p values are displayed in the plot captions. Numbers of samples in the clusters are provided in the plot legends.

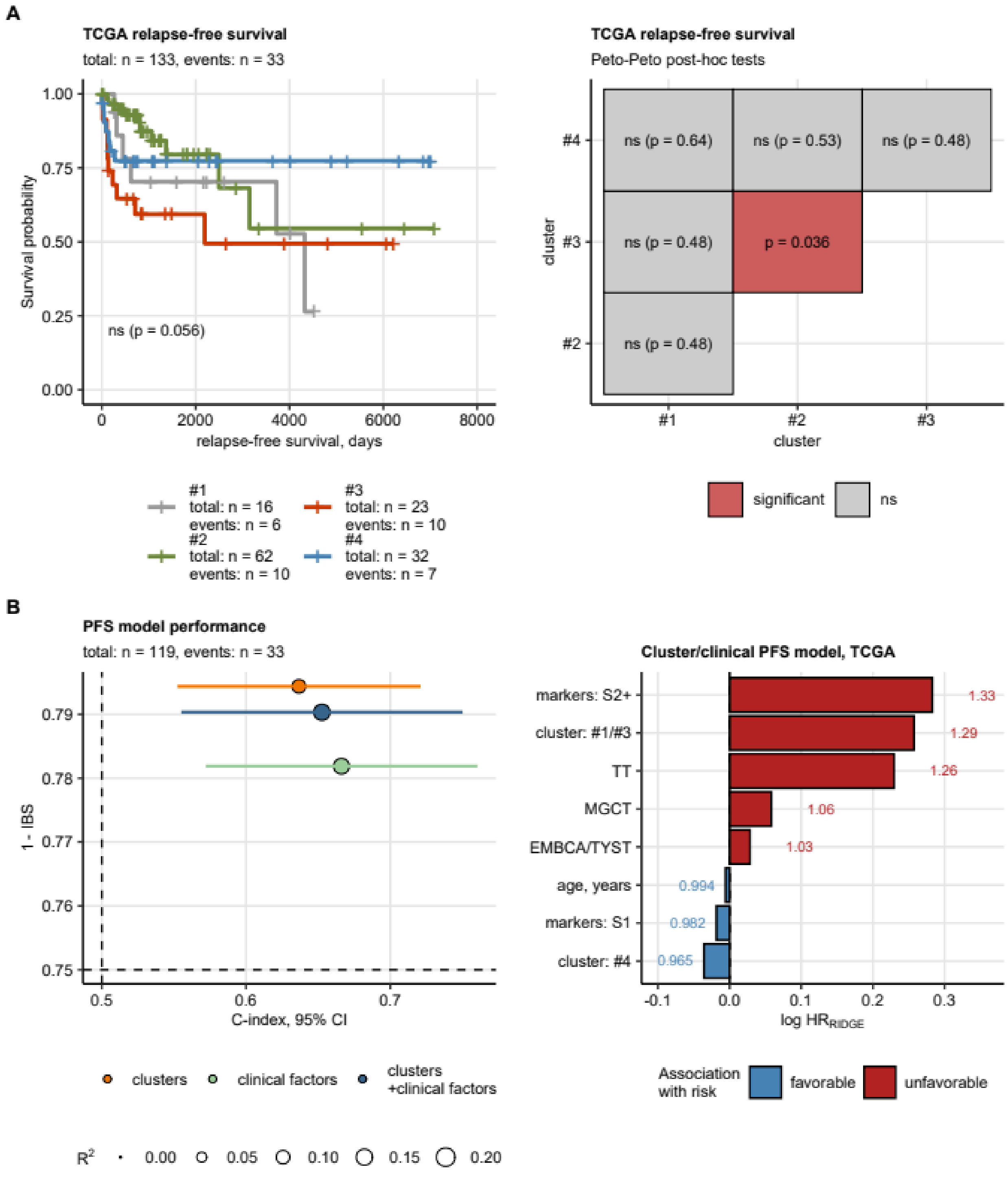

Supplementary Figure S5. Relapse-free survival in the hormonal clusters. Multi-parameter modeling of progression-free survival.

(A) *General and pairwise differences in relapse-free survival between the hormonal clusters of the TCGA cohort were assessed by Peto-Peto tests adjusted for multiple testing with the false discovery rate method. Fractions of surviving patients are visualized in a Kaplan-Meier plot with total numbers of observations and relapses indicated in the plot caption (right panel). Number of observations and relapse cases in the hormonal clusters are displayed in the Kaplan-Meier plot legend; p values for the general difference in survival is shown in the plot. P values of the pairwise comparison of survival between the hormonal clusters are displayed in a heat map (left panel).*

(B) *Multi-parameter modeling of progression-free survival (PFS) by RIDGE Cox proportional hazard regression in the TCGA cohort. Three models were constructed: (1) a model with hormonal cluster assignment (cluster #1/3 and cluster #4; baseline: cluster #2), (2) a model with clinical prognostic factors (age, serum cancer marker positivity, histological subtypes), and (3) a model with hormonal cluster assignment and the clinical prognostic factors. PFS models were evaluated by Harrell's concordance index with 95% confidence interval, integrated Brier score, and* R^2*, as measures of model predictive performance, calibration, and explanatory performance, respectively (left panel; dashed lines represent values expected for a nonsense model). Coefficient estimates for the clinical/cluster PFS model were expressed as hazard ratios (HR) and presented in a bar plot (right panel).*

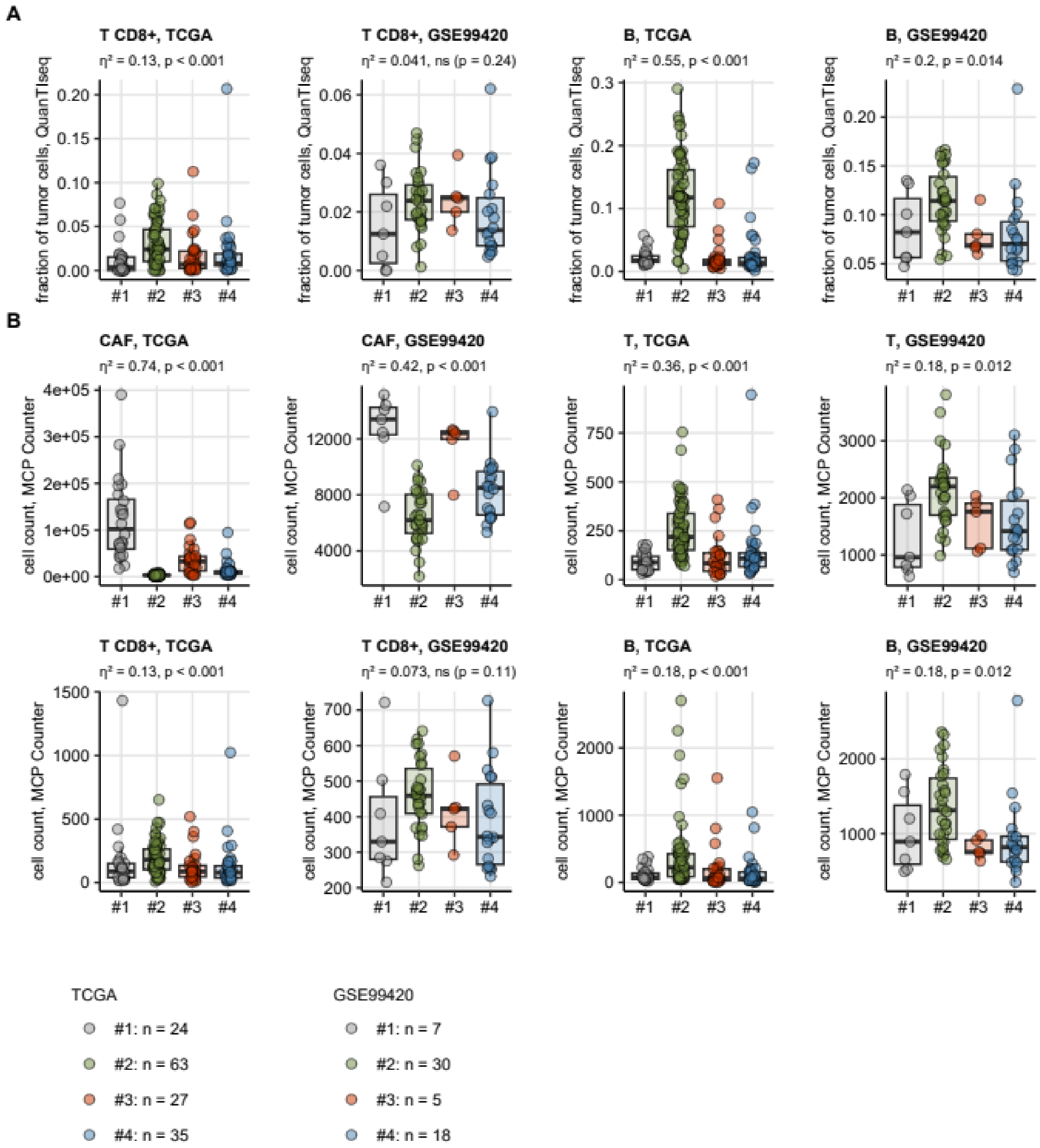

Supplementary Figure S6. Infiltration of cancer-associated fibroblasts, T and B cells in the hormonal clusters predicted by the QuanTIseq and MCP Counter algorithms.

Fractions of non-malignant cells and counts of non-malignant cells in cancer samples were estimated with the QuanTIseq and MCP Counter immunedeconvolution algorithms and compared between the hormonal clusters by Kruskal-Wallis test with η^2 effect size statistic. P values were corrected for multiple testing with the false discovery rate method. Levels of T cells (T), CD8+ T cells, B cells (B), and cancer-associated fibroblasts (CAF) were identified to differ significantly between the hormonal clusters in at least one of the TCGA or GSE99420 cohorts. Median cell levels with interquartile ranges are presented as boxes with whiskers spanning over 150% of the interquartile ranges. Single cancer samples are visualized as points. Effect sizes and p values are displayed in the plot captions. Numbers of samples in the clusters are indicated in the plot legends.

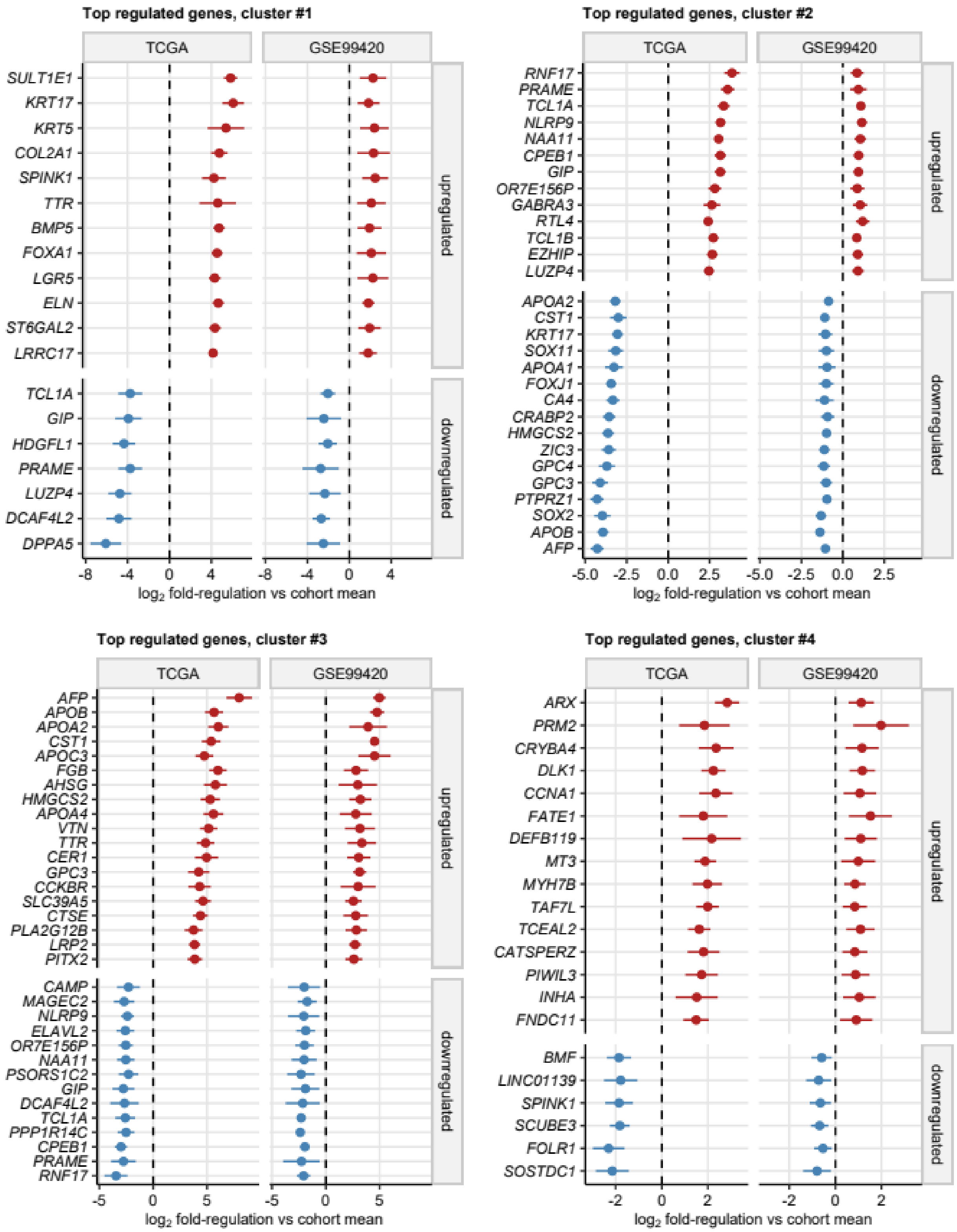
Top regulated genes, cluster #1
TCGA
GSE99420
upregulated
downregulated
SULT1E1
KRT17
KRT5
COL2A1
SPINK1
TTR
BMP5
FOXA1
LGR5
ELN
ST6GAL2
LRRC17
TCL1A
GIP
HDGFL1
PRAME
LUZP4
DCAF4L2
DPPA5
-8 -4 0 4 -8 -4 0 4
log2 fold-regulation vs cohort mean
Top regulated genes, cluster #2
TCGA
GSE99420
upregulated
downregulated
RNF17
PRAME
TCL1A
NLRP9
NAA11
CPEB1
GIP
OR7E156P
GABRA3
RTL4
TCL1B
EZHIP
LUZP4
APOA2
CST1
KRT17
SOX11
APOA1
FOXJ1
CA4
CRABP2
HMGCS2
ZIC3
GPC4
GPC3
PTPRZ1
SOX2
APOB
AFP
-5.0 -2.5 0.0 2.5 -5.0 -2.5 0.0 2.5
log2 fold-regulation vs cohort mean
Top regulated genes, cluster #3
TCGA
GSE99420
upregulated
downregulated
AFP
APOB
APOA2
CST1
APOC3
FGB
AHSG
HMGCS2
APOA4
VTN
TTR
CER1
GPC3
CCKBR
SLC39A5
CTSE
PLA2G12B
LRP2
PITX2
CAMP
MAGEC2
NLRP9
ELAVL2
OR7E156P
NAA11
PSORS1C2
GIP
DCAF4L2
TCL1A
PPP1R14C
CPEB1
PRAME
RNF17
-5 0 5 -5 0 5
log2 fold-regulation vs cohort mean
Top regulated genes, cluster #4
TCGA
GSE99420
upregulated
downregulated
ARX
PRM2
CRYBA4
DLK1
CCNA1
FATE1
DEFB119
MT3
MYH7B
TAF7L
TCEAL2
CATSPERZ
PIWIL3
INHA
FNDC11
BMF
LINC01139
SPINK1
SCUBE3
FOLR1
SOSTDC1
-2 0 2 -2 0 2
log2 fold-regulation vs cohort mean

Supplementary Figure S7. Differential gene expression in the hormonal clusters: common top regulated genes.

log_2-transformed gene expression levels were compared between the hormonal clusters of the TCGA and GSE99420 cohorts by one-way ANOVA with η^2 effect size statistic. Differences in log_2-transformed expression between the cluster and the cohort mean were assessed by one-sample T test. P values were corrected for multiple testing with the false discovery rate (FDR) method. Genes with pFDR(ANOVA) < 0.05, $\eta^2 \geq 0.14$, and pFDR(T test) < 0.05 were deemed differentially regulated. Differences in log_2 mean expression in the cluster and log_2 cohort mean expression with 95% confidence intervals for the top most strongly up- and downregulated genes in the hormonal clusters are presented in Forest plots.

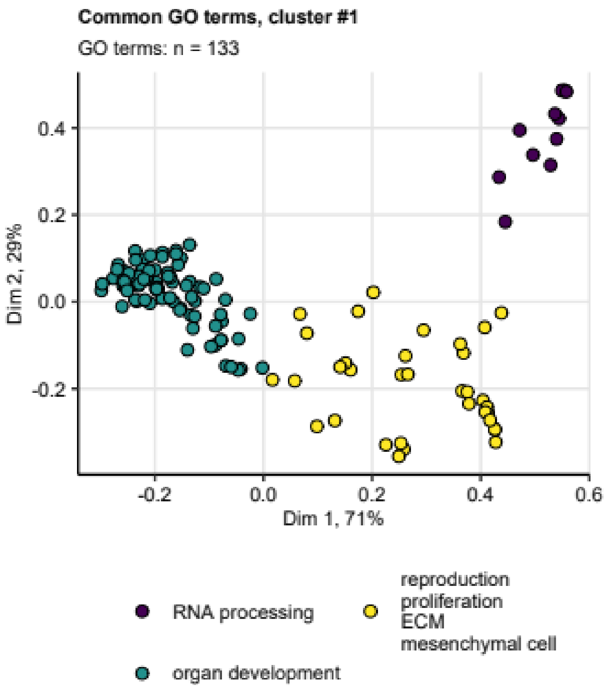
Common GO terms, cluster #1
GO terms: n = 133
Dim 2, 29%
Dim 1, 71%
0.4
0.2
0.0
-0.2
-0.2
0.0
0.2
0.4
0.6
RNA processing
reproduction
proliferation
ECM
mesenchymal cell
organ development

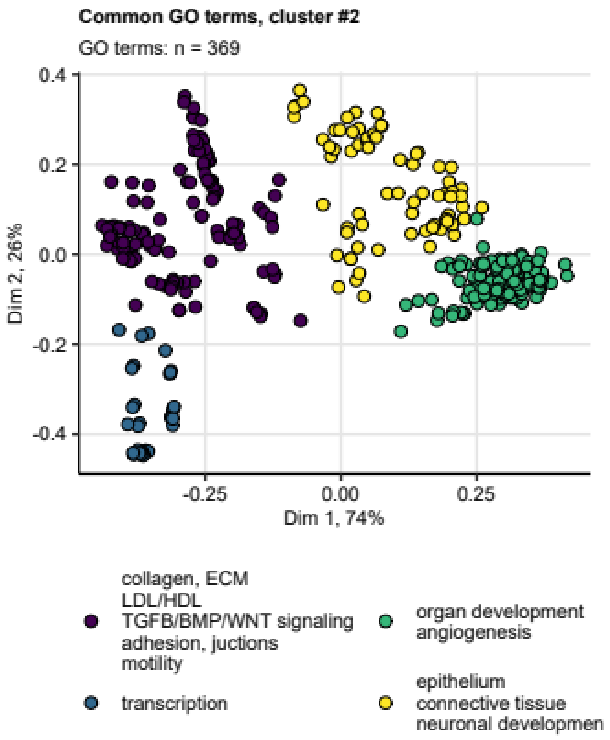
Common GO terms, cluster #2
GO terms: n = 369
Dim 2, 26%
Dim 1, 74%
0.4
0.2
0.0
-0.2
-0.4
-0.25
0.00
0.25
collagen, ECM
LDL/HDL
TGFB/BMP/WNT signaling
adhesion, juctions
motility
organ development
angiogenesis
transcription
epithelium
connective tissue
neuronal developmen

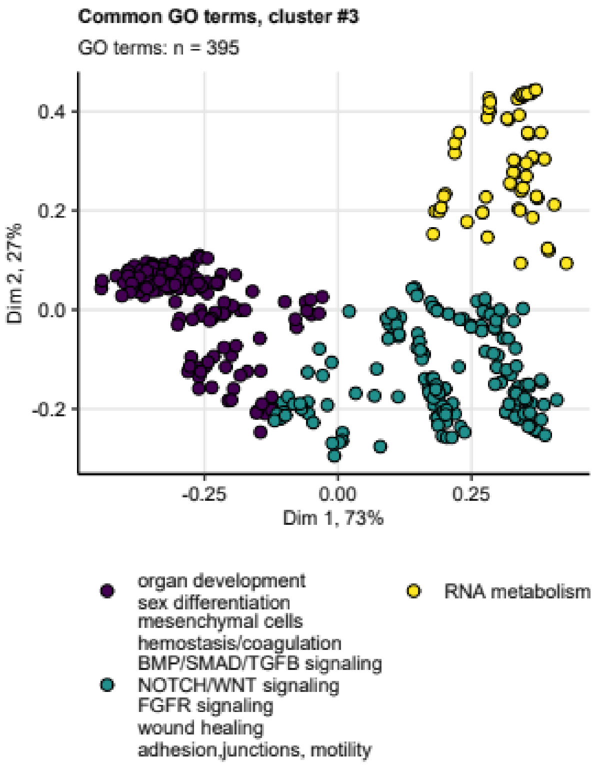
Common GO terms, cluster #3
GO terms: n = 395
Dim 2, 27%
Dim 1, 73%
0.4
0.2
0.0
-0.2
-0.25
0.00
0.25
organ development
sex differentiation
mesenchymal cells
hemostasis/coagulation
BMP/SMAD/TGFB signaling
NOTCH/WNT signaling
FGFR signaling
wound healing
adhesion,junctions, motility
RNA metabolism

Supplementary Figure S8. Biological process GO term enrichment in the hormonal clusters.

Genes found to be differentially regulated in the hormonal clusters as compared with the cohort mean were subjected to biological process gene ontology (GO) enrichment analysis. Significantly enriched GO terms shared by both the TCGA and GSE99420 cohorts were subjected to unsupervised hierarchical clustering in respect to pairwise semantic Wang distances. The clusters of those common significant GO terms were named after their characteristic biological features and visualized in two-dimensional scaling layouts of the distance matrices. Each point represents a single GO term, point color codes for semantic cluster assignment. Numbers of common cluster-enriched GO terms shared by the TCGA and GSE99420 cohorts are displayed in the plot captions.

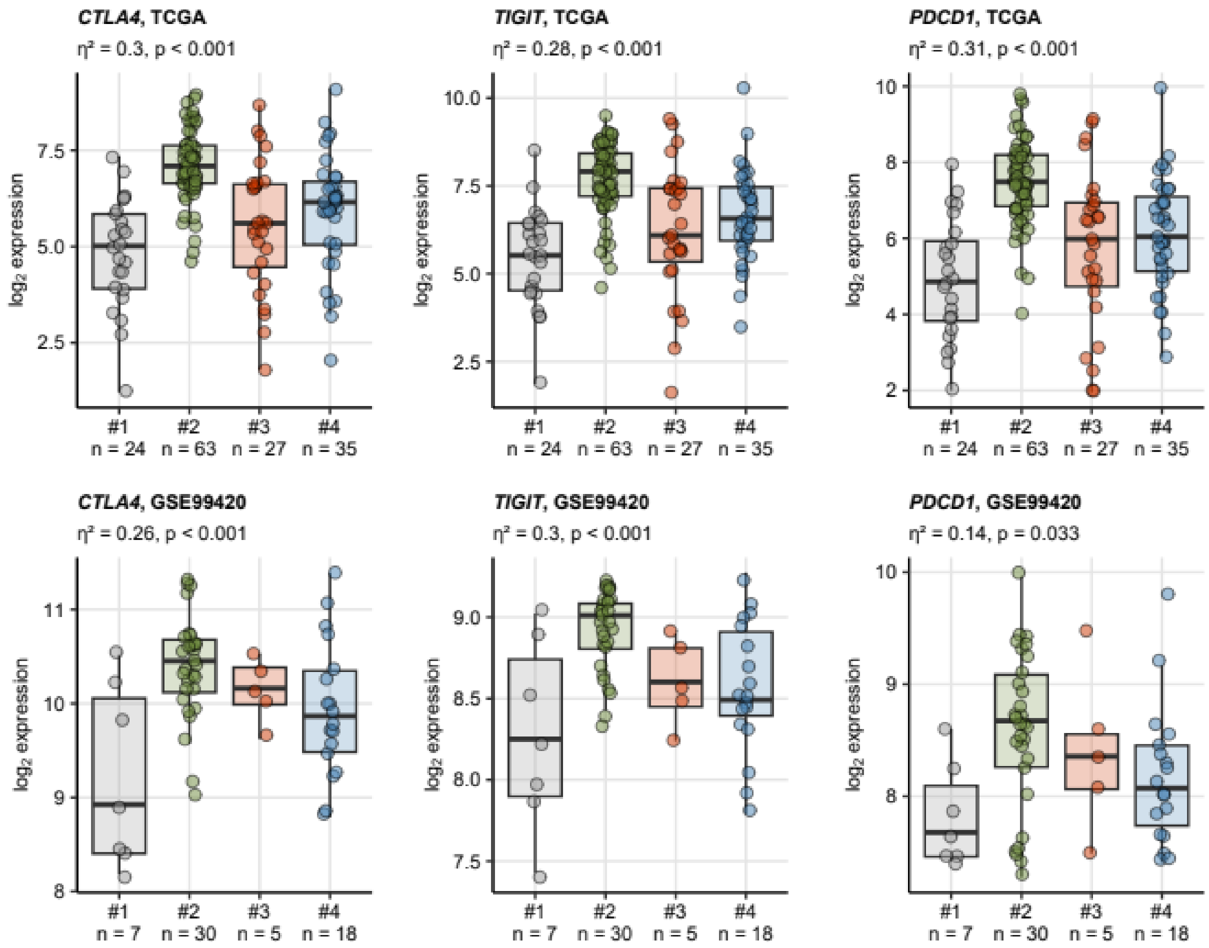

Supplementary Figure S9. Expression of genes related to immune checkpoint in the hormonal clusters.

Expression of CTLA4, TIGIT, and PDCD1 genes of relevance for immune checkpoint was found to differ significantly between the hormonal clusters of both the TCGA and GSE99420 cohort. Median log_2-transformed mRNA levels with interquartile ranges are presented as boxes with whiskers spanning over 150% of the interquartile ranges. Single cancer samples are depicted as points. Effect sizes and p values of differences between the clusters assessed by one-way ANOVA are displayed in the plot captions. Numbers of samples in the clusters are indicated in the X axes.

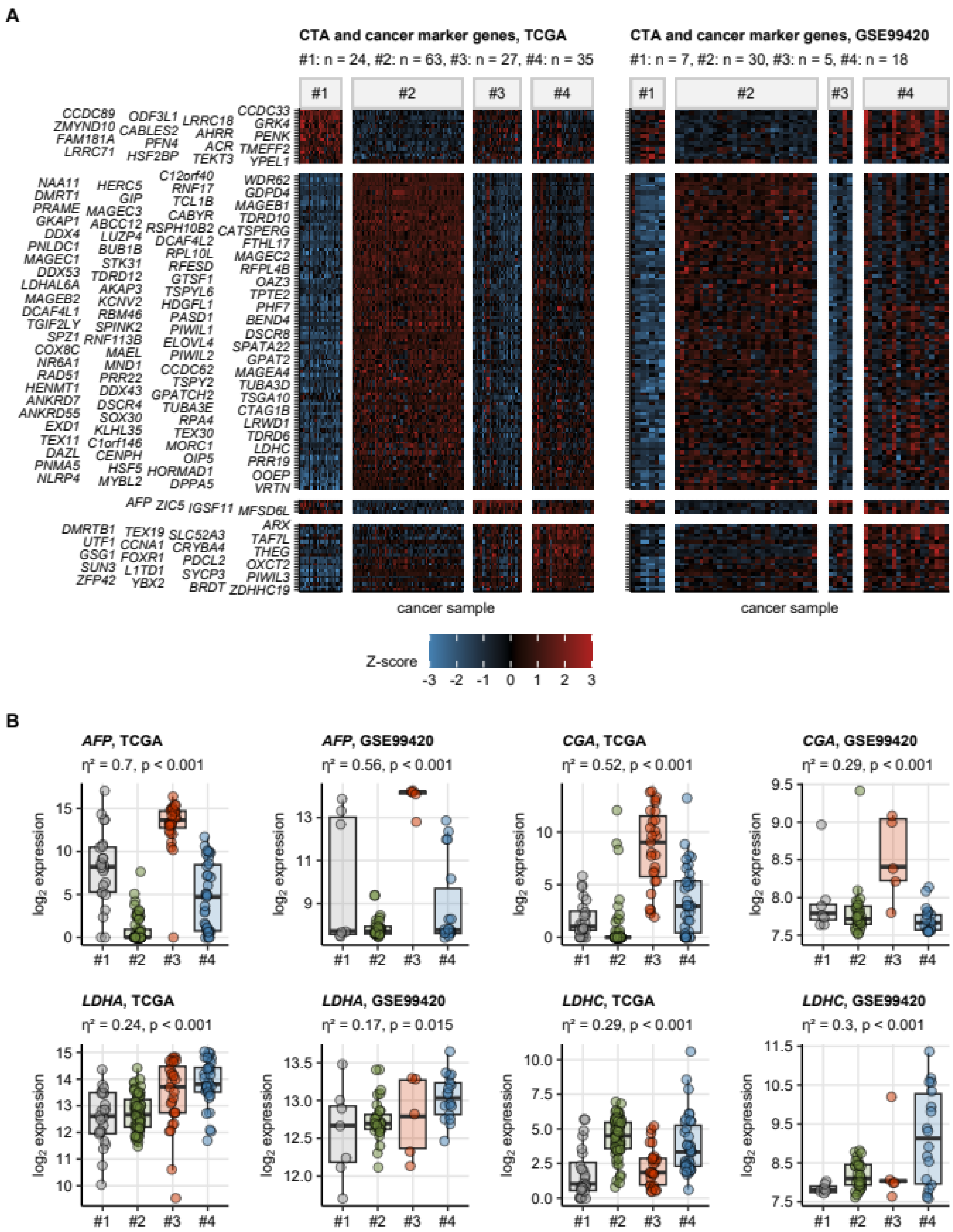
A
CTA and cancer marker genes, TCGA
#1: n = 24, #2: n = 63, #3: n = 27, #4: n = 35
CTA and cancer marker genes, GSE99420
#1: n = 7, #2: n = 30, #3: n = 5, #4: n = 18
#1
#2
#3
#4
CCDC89 ZMYND10 FAM181A LRRC71 ODF3L1 CABLES2 PFN4 HSF2BP LRRC18 AHRR ACR TEKT3 CCDC33 GRK4 PENK TMEFF2 YPEL1
NAA11 DMRT1 PRAME GKAP1 DDX4 PNLDC1 MAGEC1 DDX53 LDHAL6A MAGEB2 DCAF4L1 TGIF2LY SPZ1 COX8C NR6A1 RAD51 HENMT1 ANKRD7 ANKRD55 EXD1 TEX11 DAZL PNMA5 NLRP4
HERC5 GIP MAGEC3 ABCC12 LUZP4 BUB1B STK31 TDRD12 AKAP3 KCNV2 RBM46 SPINK2 RNF113B MAEL MND1 PRR22 DDX43 DSCR4 SOX30 KLHL35 C1orf146 CENPH HSF5 MYBL2
C12orf40 RNF17 TCL1B CABYR RSPH10B2 DCAF4L2 RPL10L RFESD GTSF1 TSPYL6 HDGFL1 PASD1 PIWIL1 ELOVL4 PIWIL2 CCDC62 TSPY2 GPATCH2 TUBA3E RPA4 TEX30 MORC1 OIP5 HORMAD1 DPPA5
WDR62 GDPD4 MAGEB1 TDRD10 CATSPERG FTHL17 MAGEC2 RFPL4B OAZ3 TPTE2 PHF7 BEND4 DSCR8 SPATA22 GPAT2 MAGEA4 TUBA3D TSGA10 CTAG1B LRWD1 TDRD6 LDHC PRR19 OOEP VRTN
AFP ZIC5 IGSF11 MFSD6L
DMRTB1 UTF1 GSG1 SUN3 ZFP42 TEX19 CCNA1 FOXR1 L1TD1 YBX2 SLC52A3 CRYBA4 PDCL2 SYCP3 BRDT ARX TAF7L THEG OXCT2 PIWIL3 ZDHHC19
cancer sample
cancer sample
Z-score
-3 -2 -1 0 1 2 3
B
AFP, TCGA
η² = 0.7, p < 0.001
AFP, GSE99420
η² = 0.56, p < 0.001
CGA, TCGA
η² = 0.52, p < 0.001
CGA, GSE99420
η² = 0.29, p < 0.001
LDHA, TCGA
η² = 0.24, p < 0.001
LDHA, GSE99420
η² = 0.17, p = 0.015
LDHC, TCGA
η² = 0.29, p < 0.001
LDHC, GSE99420
η² = 0.3, p < 0.001
log2 expression
#1 #2 #3 #4

Supplementary Figure S10. Expression of cancer testis antigens and markers of testicular carcinoma in the hormonal clusters.

Cancer testis antigens and testicular cancer markers constituted a prominent group of genes found to be differentially regulated between the hormonal clusters of both the TCGA and GSE99420.

(A) *Normalized log_2 expression (Z-scores) of cancer testis antigens and testicular markers found to be differentially regulated between the hormonal clusters in both cohorts are presented in heat maps. The genes are arranged by their maximal expression in the hormonal clusters of the TCGA cohort. Numbers of samples in the hormonal clusters are displayed in the plot captions.*

(B) *Expression of testicular cancer marker-coding genes AFP (alpha-fetoprotein), CGA (glycoprotein Hormones, alpha polypeptide), LDHA, and LDHC (A and C subunits of lactate dehydrogenase) in the hormonal clusters. Median log_2 expression values with interquartile ranges are shown in box plots with whiskers spanning over 150% of the interquartile ranges. Single cancer samples are depicted as points. Effect sizes and p values of differences between the hormonal clusters obtained by on-way ANOVA are displayed in the plot captions. Numbers of samples in the clusters are provided in (A).*

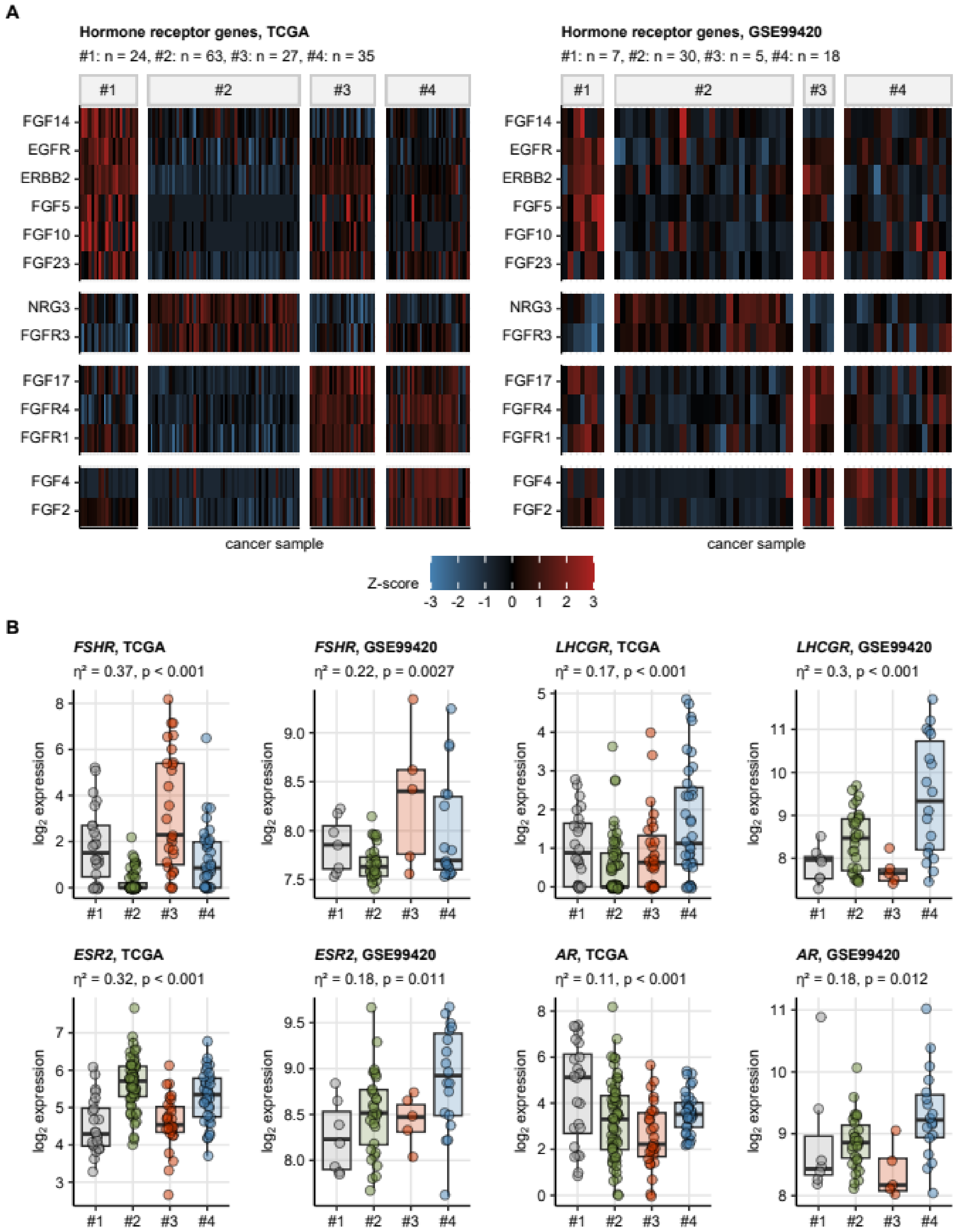
A
Hormone receptor genes, TCGA
#1: n = 24, #2: n = 63, #3: n = 27, #4: n = 35
Hormone receptor genes, GSE99420
#1: n = 7, #2: n = 30, #3: n = 5, #4: n = 18
#1
#2
#3
#4
FGF14
EGFR
ERBB2
FGF5
FGF10
FGF23
NRG3
FGFR3
FGF17
FGFR4
FGFR1
FGF4
FGF2
cancer sample
Z-score
-3 -2 -1 0 1 2 3
B
FSHR, TCGA
η² = 0.37, p < 0.001
FSHR, GSE99420
η² = 0.22, p = 0.0027
LHCGR, TCGA
η² = 0.17, p < 0.001
LHCGR, GSE99420
η² = 0.3, p < 0.001
ESR2, TCGA
η² = 0.32, p < 0.001
ESR2, GSE99420
η² = 0.18, p = 0.011
AR, TCGA
η² = 0.11, p < 0.001
AR, GSE99420
η² = 0.18, p = 0.012
log2 expression

Supplementary Figure S11. Expression of genes coding of ERBB and FGFR family receptors, ERBB/FGFR ligands, and receptors for gonadotropins and sex hormones in the hormonal clusters.

(A) *Genes coding for ERBB and FGF receptors and ligands were significantly enriched in particular hormonal clusters. Normalized* log_2*-transformed expression levels (Z-scores) of genes coding for ERBB and FGF ligands and receptors found to be differentially regulated between the clusters in both the TCGA and GSE99420 cohorts are shown in heat maps. The genes are arranged by their maximal expression in the hormonal clusters of the TCGA cohort. Numbers of samples in the clusters are indicated in the heat map captions.*

(B) *Expression of FSHR (follicle stimulating hormone receptor), LHCGR (luteinizing hormone/choriogonadotropin receptor), ESR2 (estrogen receptor beta), and AR (androgen receptor) was found to differ between the hormonal clusters in both investigated cohorts. Median* log_2 *expression values with interquartile ranges are shown in box plots with whiskers spanning over 150% of the interquartile ranges. Single cancer samples are depicted as points. Effect sizes and p values of differences between the hormonal clusters obtained by on-way ANOVA are displayed in the plot captions. Numbers of samples in the clusters are provided in (A).*

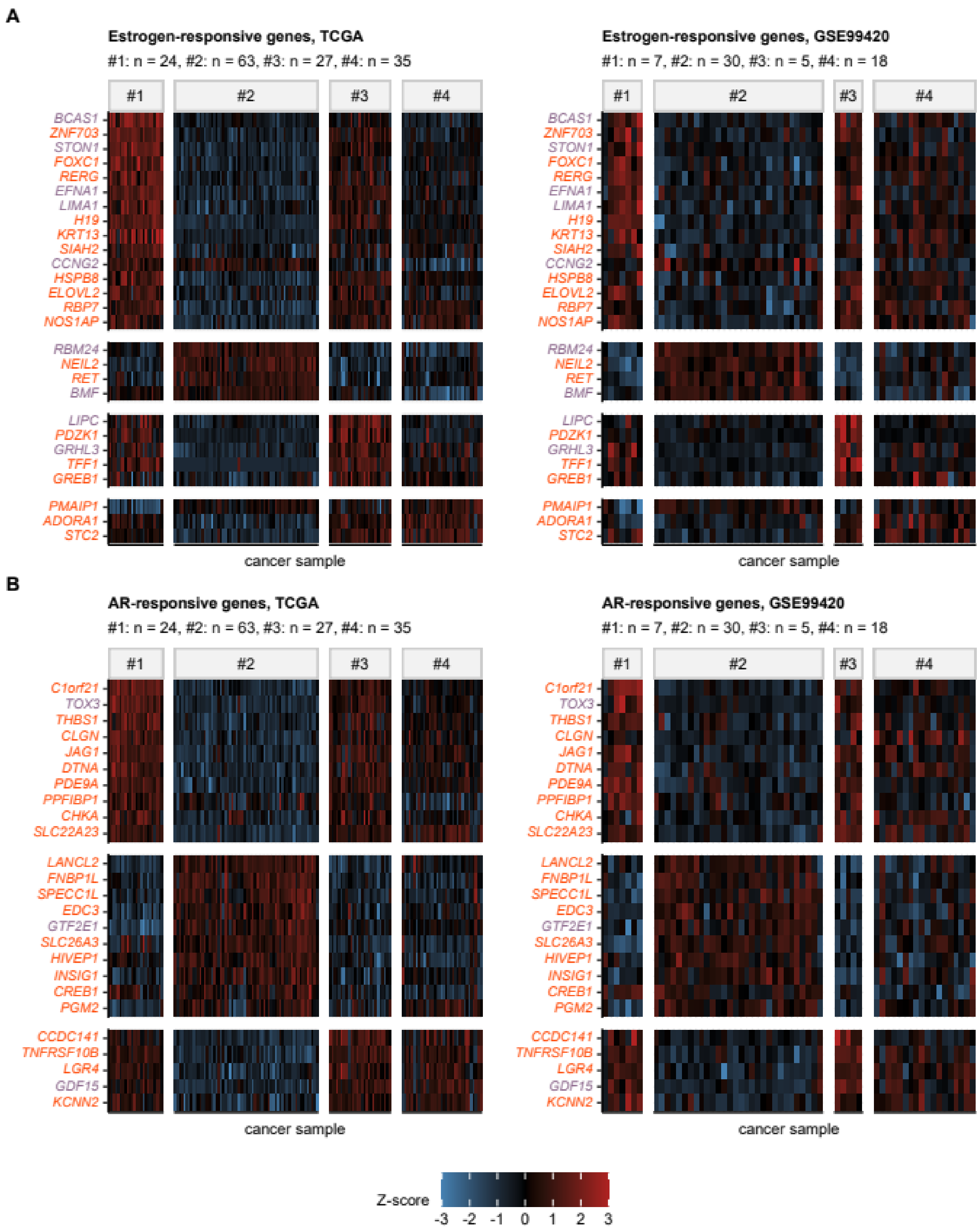
A
Estrogen-responsive genes, TCGA
#1: n = 24, #2: n = 63, #3: n = 27, #4: n = 35
#1
#2
#3
#4
BCAS1
ZNF703
STON1
FOXC1
RERG
EFNA1
LIMA1
H19
KRT13
SIAH2
CCNG2
HSPB8
ELOVL2
RBP7
NOS1AP
RBM24
NEIL2
RET
BMF
LIPC
PDZK1
GRHL3
TFF1
GREB1
PMAIP1
ADORA1
STC2
cancer sample
Estrogen-responsive genes, GSE99420
#1: n = 7, #2: n = 30, #3: n = 5, #4: n = 18
cancer sample
B
AR-responsive genes, TCGA
#1: n = 24, #2: n = 63, #3: n = 27, #4: n = 35
C1orf21
TOX3
THBS1
CLGN
JAG1
DTNA
PDE9A
PPFIBP1
CHKA
SLC22A23
LANCL2
FNBP1L
SPECC1L
EDC3
GTF2E1
SLC26A3
HIVEP1
INSIG1
CREB1
PGM2
CCDC141
TNFRSF10B
LGR4
GDF15
KCNN2
cancer sample
AR-responsive genes, GSE99420
#1: n = 7, #2: n = 30, #3: n = 5, #4: n = 18
cancer sample
Z-score
-3 -2 -1 0 1 2 3

Supplementary Figure S12. Expression of estrogen- and androgen-responsive genes in the hormonal clusters.

Normalized log_2-transformed expression levels (Z-scores) of estrogen- (A, investigated: n = 87, common regulated: n = 42) and androgen-responsive genes (B, investigated: n = 177, common regulated: n = 46) found to be differentially regulated between the hormonal clusters in the TCGA and GSE99420 cohort are presented in heat maps. Font color of the gene symbols represents the gene response sign reported in literature; genes reported to be upregulated are labeled with orange font, genes reported to be downregulated by the sex hormones are labeled with violet font. Numbers of samples in the clusters are displayed in the plot captions.

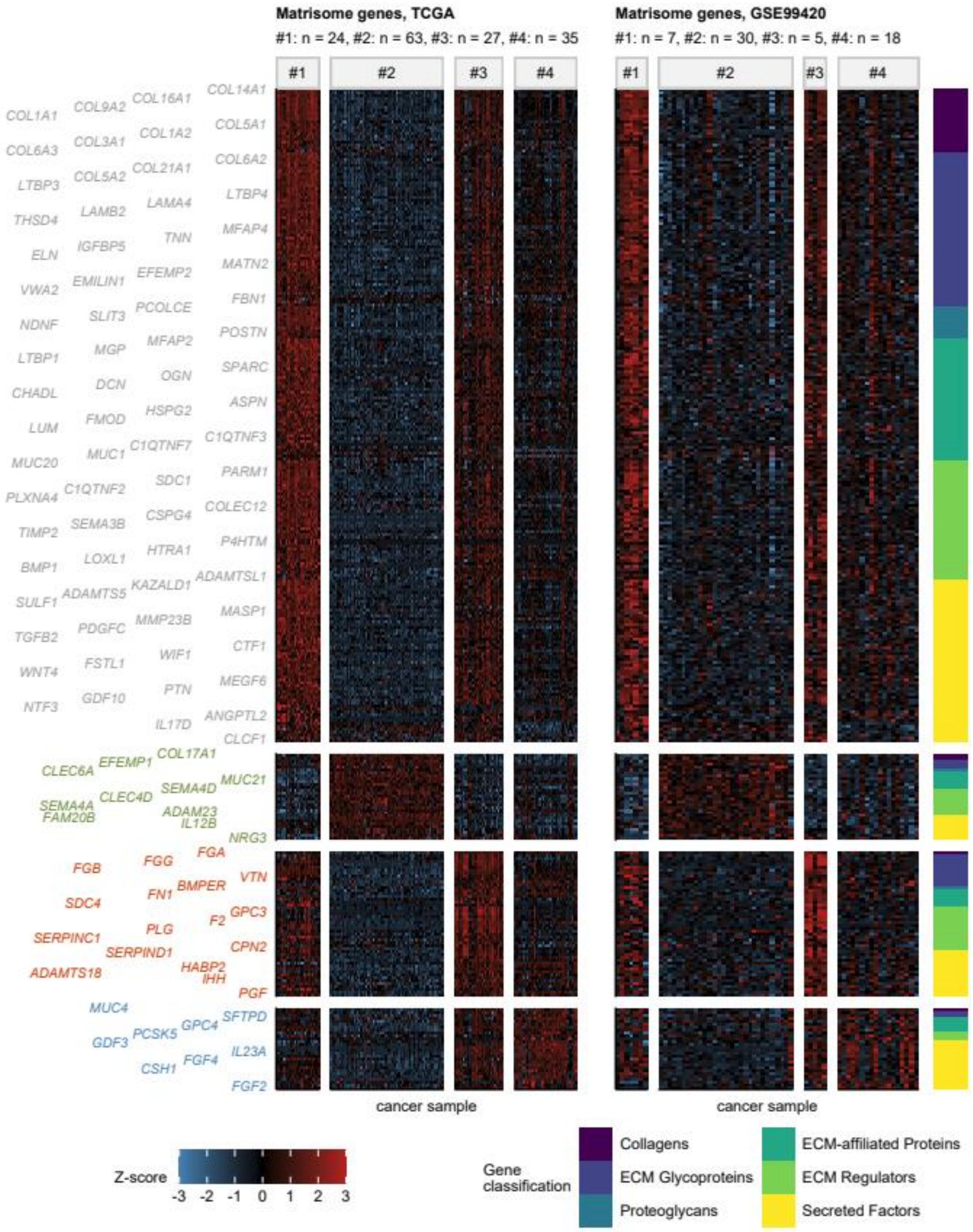
Matrisome genes, TCGA
#1: n = 24, #2: n = 63, #3: n = 27, #4: n = 35
Matrisome genes, GSE99420
#1: n = 7, #2: n = 30, #3: n = 5, #4: n = 18
#1
#2
#3
#4
COL1A1
COL9A2
COL16A1
COL14A1
COL6A3
COL3A1
COL1A2
COL5A1
LTBP3
COL5A2
COL21A1
COL6A2
THSD4
LAMB2
LAMA4
LTBP4
ELN
IGFBP5
TNN
MFAP4
VWA2
EMILIN1
EFEMP2
MATN2
NDNF
SLIT3
PCOLCE
FBN1
LTBP1
MGP
MFAP2
POSTN
CHADL
DCN
OGN
SPARC
LUM
FMOD
HSPG2
ASPN
MUC20
MUC1
C1QTNF7
C1QTNF3
PLXNA4
C1QTNF2
SDC1
PARM1
TIMP2
SEMA3B
CSPG4
COLEC12
BMP1
LOXL1
HTRA1
P4HTM
SULF1
ADAMTS5
KAZALD1
ADAMTSL1
TGFB2
PDGFC
MMP23B
MASP1
WNT4
FSTL1
WIF1
CTF1
NTF3
GDF10
PTN
MEGF6
IL17D
ANGPTL2
CLCF1
CLEC6A
EFEMP1
COL17A1
MUC21
SEMA4A
CLEC4D
SEMA4D
FAM20B
ADAM23
IL12B
NRG3
FGB
FGG
FGA
VTN
SDC4
FN1
BMPER
GPC3
SERPINC1
PLG
F2
CPN2
SERPIND1
ADAMTS18
HABP2
IHH
PGF
MUC4
SFTPD
GDF3
PCSK5
GPC4
IL23A
CSH1
FGF4
FGF2
cancer sample
cancer sample
Z-score
-3 -2 -1 0 1 2 3
Gene classification
Collagens
ECM Glycoproteins
Proteoglycans
ECM-affiliated Proteins
ECM Regulators
Secreted Factors

Supplementary Figure S13. Expression of matrisome genes in the hormonal clusters of testicular cancer.

Among 1001 matrisome genes, i.e. genes coding for components of extracellular matrix (ECM), 333 genes were found to be differentially regulated between the hormonal clusters in both the TCGA and GSE99420 cohorts. Normalized log_2*-transformed expression (Z-scores) of those common regulated genes in the hormonal clusters was presented in heat maps. Genes were arranged by their peak expression in the hormonal clusters of the TCGA cohort. Top strongest differentially regulated genes in particular clusters are shown in word clouds next to the Y axis of the heat maps (gray: genes regulated in cluster #1, green: cluster #2, red: cluster #3, blue: cluster #4). Classification of the common regulated matrisome genes into collagens, ECM glycoproteins, proteoglycans, ECM-affiliated proteins, ECM regulators, and secreted factors is color coded in the vertical rug plot.*

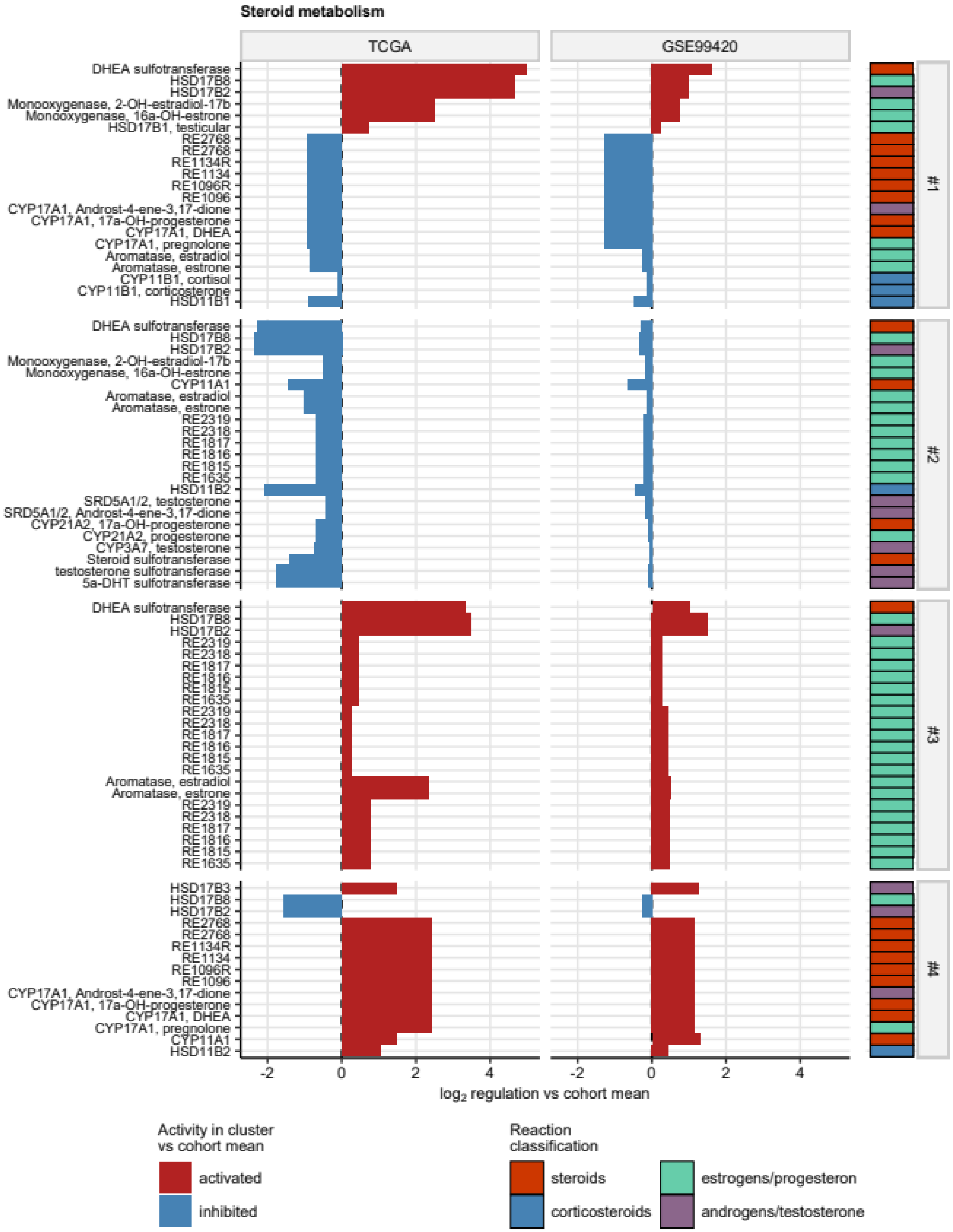
Steroid metabolism
TCGA
GSE99420
#1
#2
#3
#4
DHEA sulfotransferase
HSD17B8
HSD17B2
Monooxygenase, 2-OH-estradiol-17b
Monooxygenase, 16a-OH-estrone
HSD17B1, testicular
RE2768
RE1134R
RE1134
RE1096R
RE1096
CYP17A1, Androst-4-ene-3,17-dione
CYP17A1, 17a-OH-progesterone
CYP17A1, DHEA
CYP17A1, pregnolone
Aromatase, estradiol
Aromatase, estrone
CYP11B1, cortisol
CYP11B1, corticosterone
HSD11B1
CYP11A1
RE2319
RE2318
RE1817
RE1816
RE1815
RE1635
HSD11B2
SRD5A1/2, testosterone
SRD5A1/2, Androst-4-ene-3,17-dione
CYP21A2, 17a-OH-progesterone
CYP21A2, progesterone
CYP3A7, testosterone
Steroid sulfotransferase
testosterone sulfotransferase
5a-DHT sulfotransferase
HSD17B3
-2
0
2
4
log2 regulation vs cohort mean
Activity in cluster vs cohort mean
activated
inhibited
Reaction classification
steroids
corticosteroids
estrogens/progesteron
androgens/testosterone

Supplementary Figure S14. Predicted activity of steroid metabolism reactions in the hormonal clusters.

Modulation of RECON2 model metabolic reactions in the hormonal clusters as compared with the cohort mean was modeled by Monte Carlo simulation provided with log_2 *fold-regulation estimates of differential expression with their standard errors for all available genes.* log_2 *fold-regulation estimates of activity of reactions of steroid and steroid hormone metabolism found to be significantly activated or inhibited in the hormonal clusters as compared with the cohort mean in both the TCGA and GSE99420 cohorts are presented in bar plots. Functional classification of the reactions is color coded in the vertical rug plot. Reactions are labeled with their official names or, if the name was not available, with BIGG/RECON identifiers.*

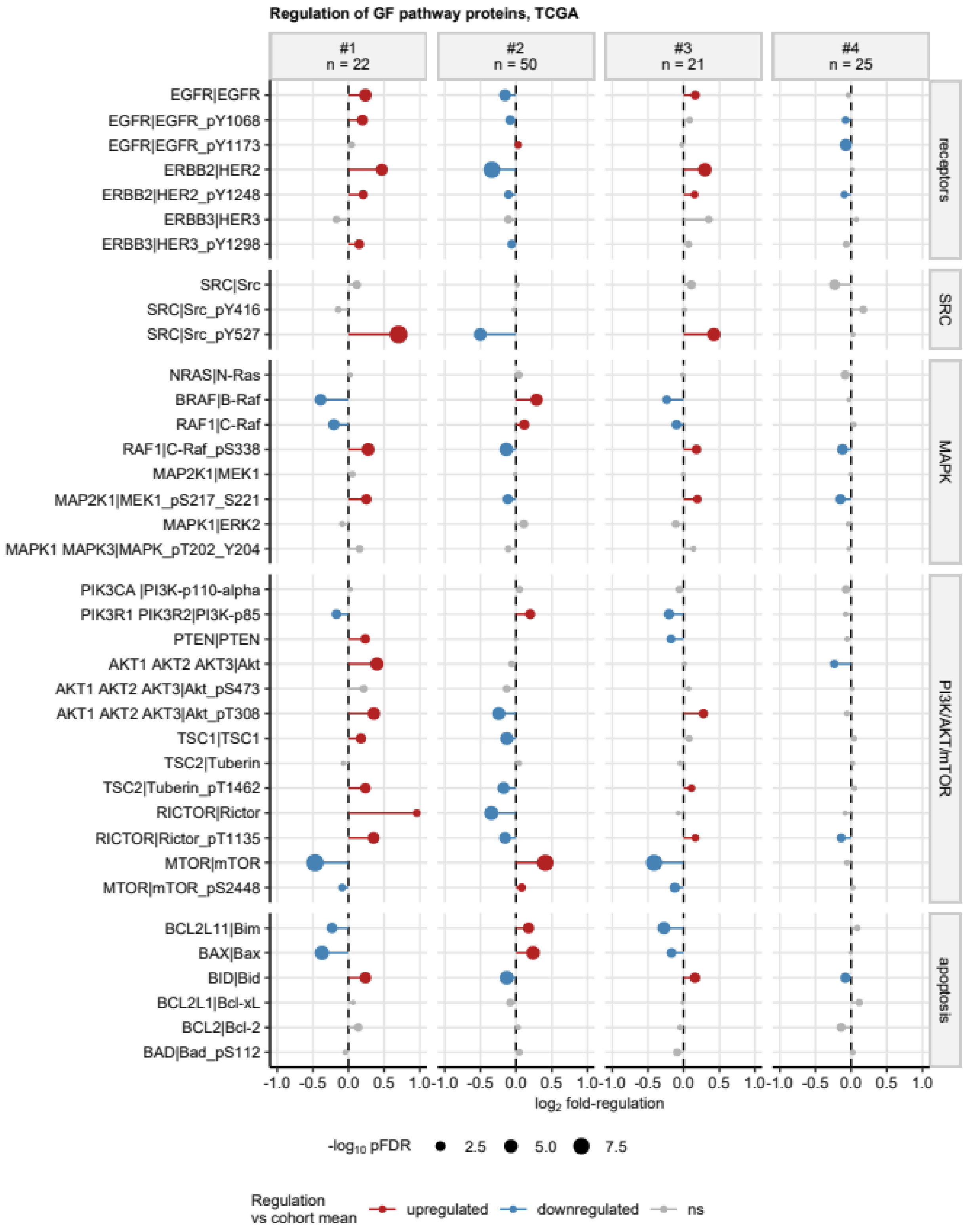
Regulation of GF pathway proteins, TCGA
#1 n = 22
#2 n = 50
#3 n = 21
#4 n = 25
EGFR|EGFR
EGFR|EGFR_pY1068
EGFR|EGFR_pY1173
ERBB2|HER2
ERBB2|HER2_pY1248
ERBB3|HER3
ERBB3|HER3_pY1298
SRC|Src
SRC|Src_pY416
SRC|Src_pY527
NRAS|N-Ras
BRAF|B-Raf
RAF1|C-Raf
RAF1|C-Raf_pS338
MAP2K1|MEK1
MAP2K1|MEK1_pS217_S221
MAPK1|ERK2
MAPK1 MAPK3|MAPK_pT202_Y204
PIK3CA |PI3K-p110-alpha
PIK3R1 PIK3R2|PI3K-p85
PTEN|PTEN
AKT1 AKT2 AKT3|Akt
AKT1 AKT2 AKT3|Akt_pS473
AKT1 AKT2 AKT3|Akt_pT308
TSC1|TSC1
TSC2|Tuberin
TSC2|Tuberin_pT1462
RICTOR|Rictor
RICTOR|Rictor_pT1135
MTOR|mTOR
MTOR|mTOR_pS2448
BCL2L11|Bim
BAX|Bax
BID|Bid
BCL2L1|Bcl-xL
BCL2|Bcl-2
BAD|Bad_pS112
receptors
SRC
MAPK
PI3K/AKT/mTOR
apoptosis
-1.0 -0.5 0.0 0.5 1.0
log2 fold-regulation
-log10 pFDR 2.5 5.0 7.5
Regulation vs cohort mean
upregulated
downregulated
ns

Supplementary Figure S15. Regulation of growth factor pathway signaling proteins in the hormonal clusters.

Expression of 194 cancer biology-relevant proteins was investigated in cancer samples of the TCGA cohort with reverse phase protein array. log_2-transformed expression levels were compared between the hormonal clusters by one-way ANOVA with η^2 effect size statistic. Differences in log_2-transformed expression between the cluster and the cohort mean were assessed by one-sample T test. P values were corrected for multiple testing with the false discovery rate (FDR) method. Proteins with pFDR(ANOVA) < 0.05, $\eta^2 \geq 0.14$, and pFDR(T test) < 0.05 were deemed differentially regulated.

log_2 fold-regulation estimates of levels of proteins of the EGFR/ERBB signaling pathways in the clusters as compared with the respective cohort averages are presented in a dot plot. Point and segment colors code for significance and regulation sign. FDR-corrected p values are coded by point size.

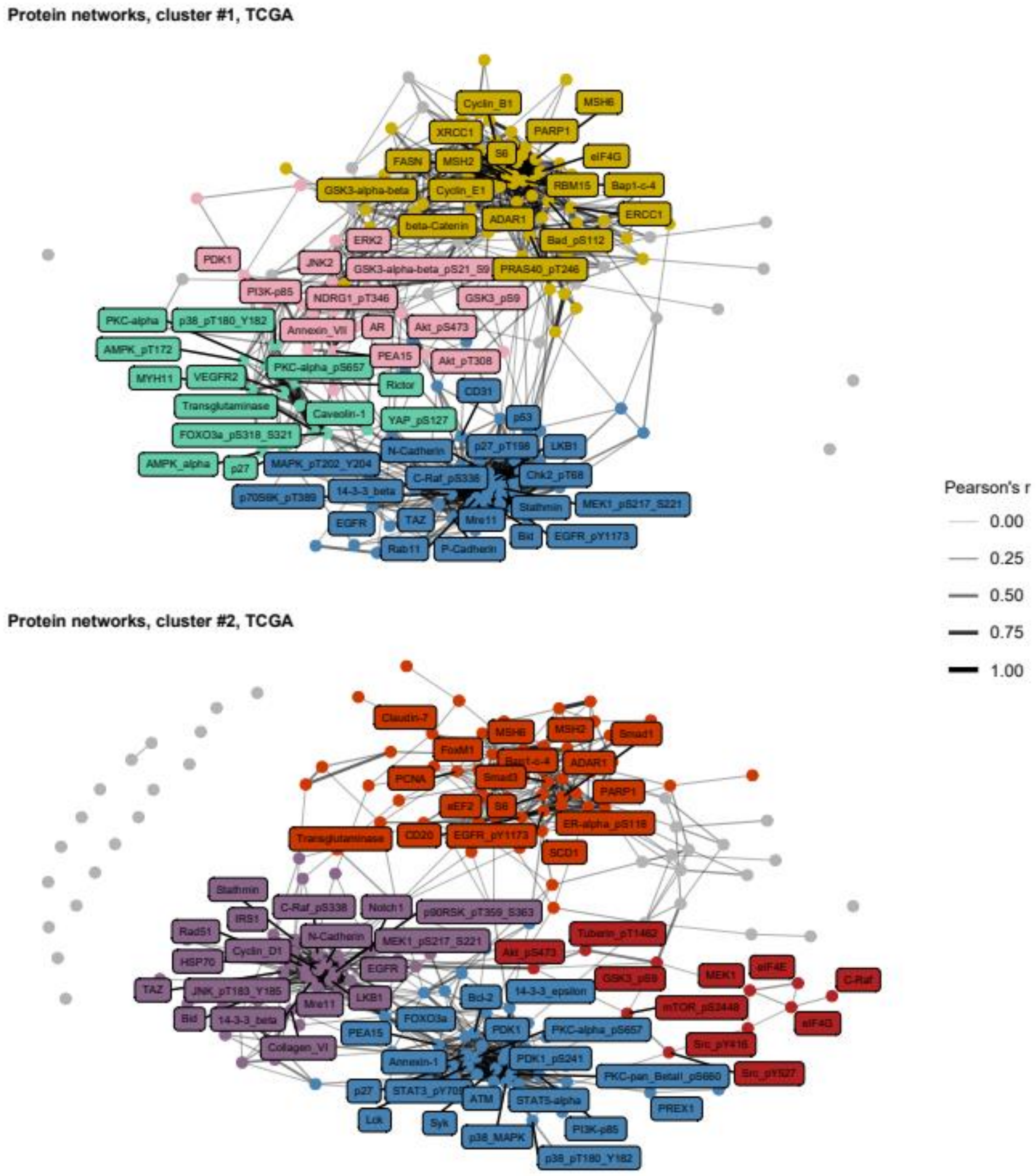

Supplementary Figure S16. Co-expression protein networks in the hormonal clusters of the TCGA cohort: clusters #1 and #2.

Expression of 194 cancer biology-relevant proteins was investigated in cancer samples of the TCGA cohort with reverse phase protein array. Pairwise correlation of log_2-transformed protein levels in the hormonal clusters was investigated by Pearson's r correlation

coefficients. Associations with r ≥ 0.5 were analyzed and visualized as undirected graphs. Graph nodes are depicted as points, whose colors code for protein community (i.e. cluster of inter-connected nodes). Edge color and width codes for Pearson's r value. Proteins with the largest node importance statistics (degree, betweenness, and hub score) are labeled in the plot with their symbols.

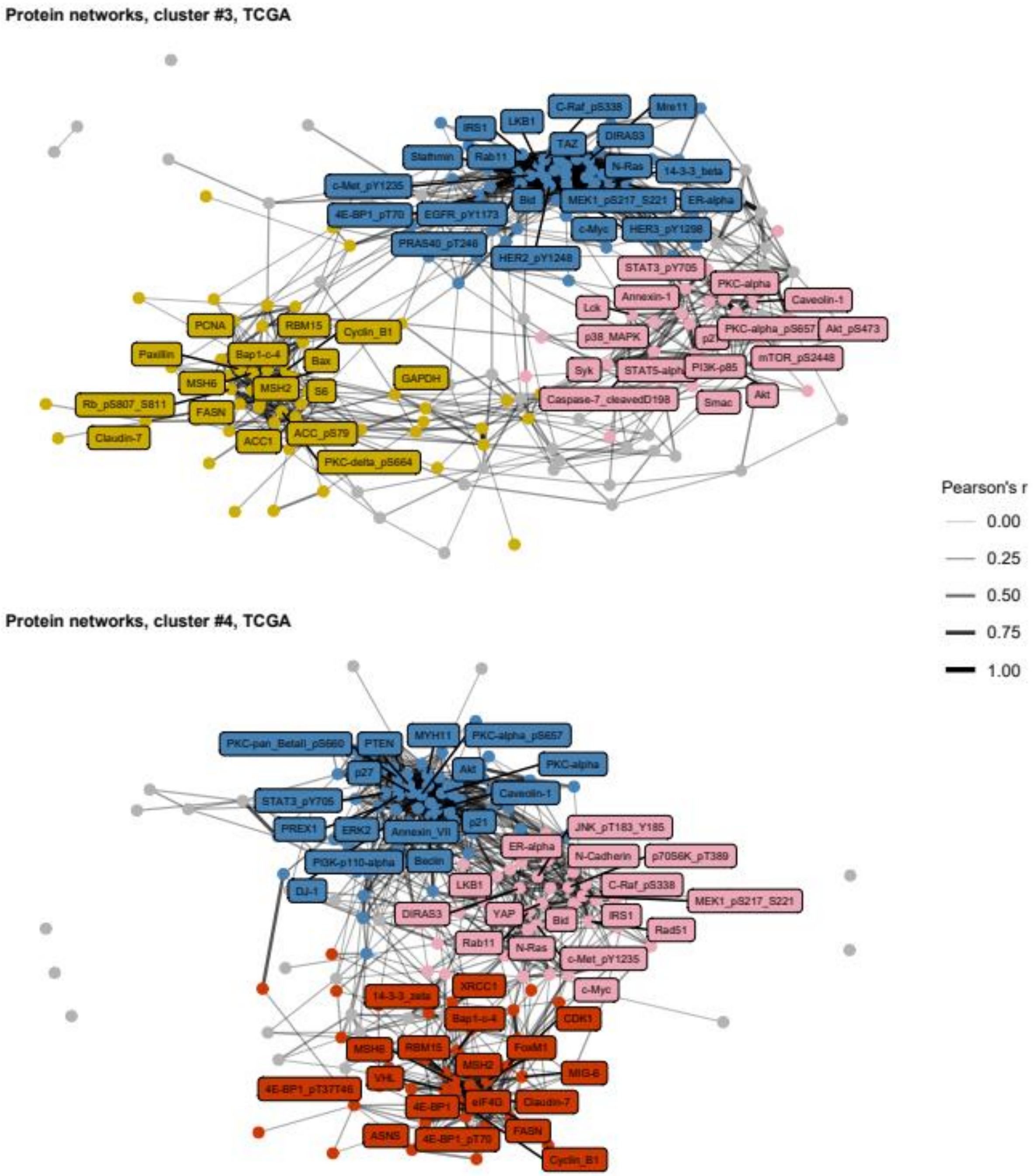

Supplementary Figure S17. Co-expression protein networks in the hormonal clusters of the TCGA cohort: clusters #3 and #4.

Expression of 194 cancer biology-relevant proteins was investigated in cancer samples of the TCGA cohort with reverse phase protein array. Pairwise correlation of log_2-transformed protein levels in the hormonal clusters was investigated by Pearson's r correlation

coefficients. Associations with $r \geq 0.5$ were analyzed and visualized as undirected graphs. Graph nodes are depicted as points, whose colors code for protein community (i.e. cluster of inter-connected nodes). Edge color and width codes for Pearson's r value. Proteins with the largest node importance statistics (degree, betweenness, and hub score) are labeled in the plot with their symbols.

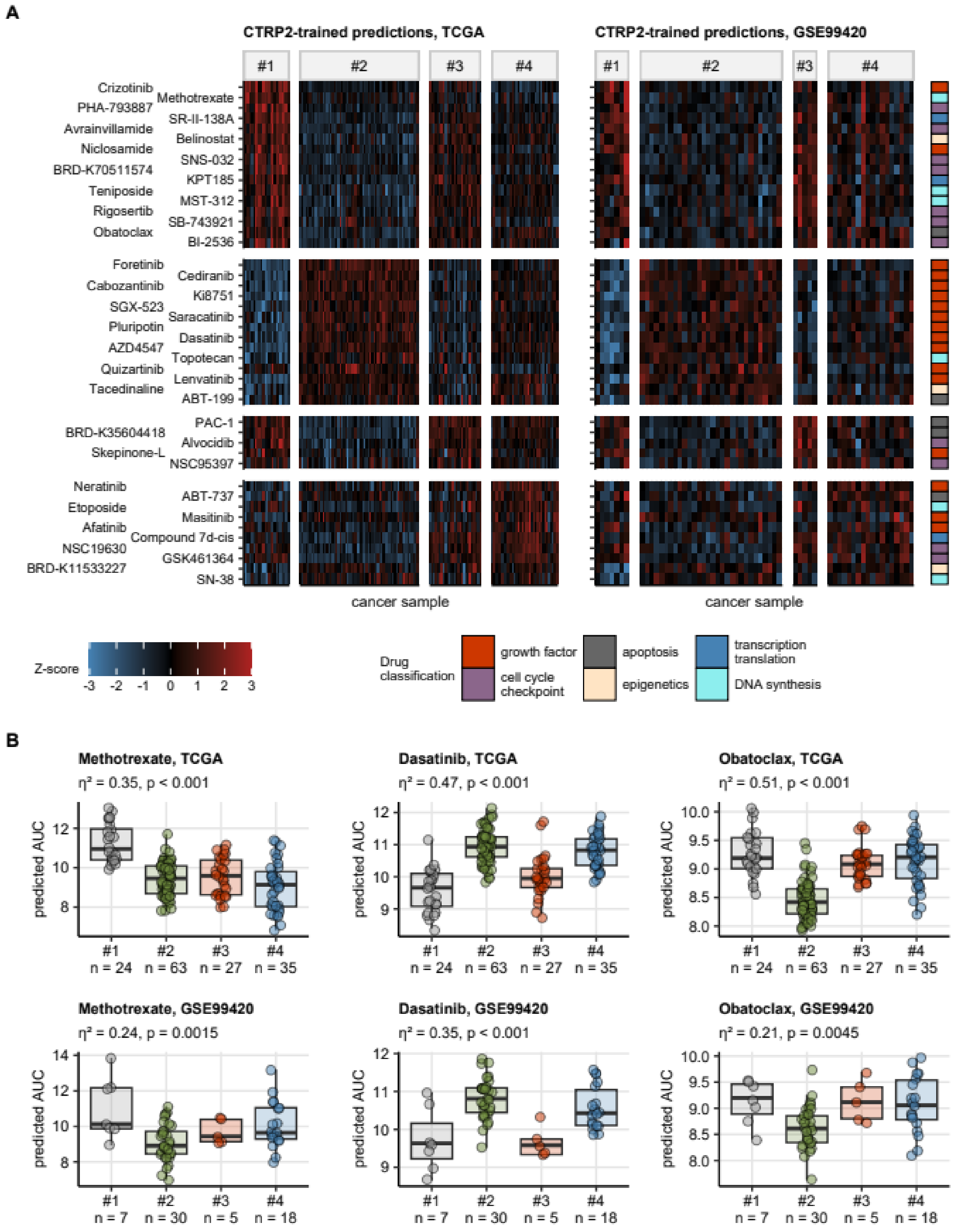
A
CTRP2-trained predictions, TCGA
CTRP2-trained predictions, GSE99420
#1
#2
#3
#4
Crizotinib
PHA-793887
Avrainvillamide
Niclosamide
BRD-K70511574
Teniposide
Rigosertib
Obatoclax
Methotrexate
SR-II-138A
Belinostat
SNS-032
KPT185
MST-312
SB-743921
BI-2536
Foretinib
Cabozantinib
SGX-523
Pluripotin
AZD4547
Quizartinib
Tacedinaline
Cediranib
Ki8751
Saracatinib
Dasatinib
Topotecan
Lenvatinib
ABT-199
BRD-K35604418
Skepinone-L
PAC-1
Alvocidib
NSC95397
Neratinib
Etoposide
Afatinib
NSC19630
BRD-K11533227
ABT-737
Masitinib
Compound 7d-cis
GSK461364
SN-38
cancer sample
Z-score
-3 -2 -1 0 1 2 3
Drug classification
growth factor
cell cycle checkpoint
apoptosis
epigenetics
transcription translation
DNA synthesis
B
Methotrexate, TCGA
η² = 0.35, p < 0.001
Dasatinib, TCGA
η² = 0.47, p < 0.001
Obatoclax, TCGA
η² = 0.51, p < 0.001
predicted AUC
n = 24
n = 63
n = 27
n = 35
Methotrexate, GSE99420
η² = 0.24, p = 0.0015
Dasatinib, GSE99420
η² = 0.35, p < 0.001
Obatoclax, GSE99420
η² = 0.21, p = 0.0045
n = 7
n = 30
n = 5
n = 18

Supplementary Figure S18. Predicted anti-cancer drug response in the hormonal clusters.

Anti-cancer drug response in form of AUC (are under the dose-response curve) in cancer samples was predicted by RIDGE linear models trained with the CTRP2 in vitro drug screening data set. The AUC values were compared between the hormonal clusters by one-way ANOVA with η^2 effect size statistic. Differences in the AUC values between the cluster and the cohort mean were assessed by one-sample T test. P values were corrected for multiple testing with the false discovery rate (FDR) method. Compounds with pFDR(ANOVA) < 0.05, $\eta^2 \geq 0.14$, and pFDR(T test) < 0.05 were considered significant.

(A) *Normalized AUC values (Z-scores) of compounds identified as significant in both the TCGA and GSE99420 cohort are presented in heat maps. The compounds are arranged by their peak expression in the hormonal clusters of the TCGA cohort. Compound classification is color coded in the vertical rug plot.*

(B) *Predicted AUC for representative compounds interfering with nucleotide and DNA synthesis (metothrexate), growth factor signaling (dasatinib), and apoptosis pathways (obatoclax). Median AUC values with interquartile ranges are visualized as boxes with whiskers spanning over 150% of the interquartile ranges. Single cancer samples are depicted as points. Effect sizes and p values for differences between the clusters investigated by one-way ANOVA are displayed in the plot captions. Numbers of samples in the clusters are indicated in the X axes.*

References

1. Wickham H, Averick M, Bryan J, Chang W, McGowan L, François R, Grolemund G, Hayes A, Henry L, Hester J, et al. Welcome to the Tidyverse. *Journal of Open Source Software* (2019) 4:1686. doi: 10.21105/joss.01686

2. Henry L, Wickham Hadley. rlang: Functions for Base Types and Core R and 'Tidyverse' Features. (2022) https://cran.r-project.org/web/packages/rlang/index.html

3. Gagolewski M, Tartanus B. Package 'stringi'. (2021) https://cran.r-project.org/web/packages/stringi/index.html http://cran.ism.ac.jp/web/packages/stringi/stringi.pdf

4. Kassambara A. rstatix: Pipe-Friendly Framework for Basic Statistical Tests. (2021) https://cran.r-project.org/package=rstatix

5. Mangiafico S. rcompanion: Functions to Support Extension Education Program Evaluation. (2022) https://cran.r-project.org/package=rcompanion

6. Kuhn M. Building predictive models in R using the caret package. *Journal of Statistical Software* (2008) 28:1–26. doi: 10.18637/jss.v028.i05

7. Signorell A. DescTools: Tools for Descriptive Statistics. (2022) https://cran.r-project.org/package=DescTools

8. Friedman J, Hastie T, Tibshirani R. Regularization paths for generalized linear models via coordinate descent. *Journal of Statistical Software* (2010) 33:1–22. doi: 10.18637/jss.v033.i01

9. Csardi G, Nepusz T. The igraph software package for complex network research. *InterJournal* (2006) Complex Sy:1695. https://igraph.org

10. Briatte F, Bojanowski M, Canouil M, Charlop-Powers Z, Fisher JC, Johnson K, Rinker T. ggnetwork: Geometries to Plot Networks with 'ggplot2'. (2021) https://cran.r-project.org/package=ggnetwork

11. Schubert E, Rousseeuw PJ. Faster k-Medoids Clustering: Improving the PAM, CLARA, and CLARANS Algorithms. *Lecture notes in computer science (including subseries lecture notes in artificial intelligence and lecture notes in bioinformatics)*. Springer (2019). p. 171–187 doi: 10.1007/978-3-030-32047-8_16

12. Raymaekers J, Zamar RH. Regularized K-means Through Hard-Thresholding. *Journal of Machine Learning Research* (2022) 23:1–48. http://jmlr.org/papers/v23/21-0052.html

13. Wright MN, Ziegler A. ranger: A Fast Implementation of Random Forests for High Dimensional Data in C++ and R. *Journal of Statistical Software* (2017) 77:1–17. doi: 10.18637/JSS.V077.I01

14. Therneau TM, Grambsch PM. *Modeling Survival Data: Extending the Cox Model*. 1st ed. New York: Springer Verlag (2000).

15. Kassambara A, Kosinski M, Biecek P. survminer: Drawing Survival Curves using ’ggplot2’. (2016) https://cran.r-project.org/package=survminer

16. Harrell FE. rms: Regression Modeling Strategies. (2023) https://cran.r-project.org/web/packages/rms/index.html

17. Müller-Dott S, Tsirvouli E, Vazquez M, Ramirez Flores RO, Badia-i-Mompel P, Fallegger R, Türei D, Lægreid A, Saez-Rodriguez J. Expanding the coverage of regulons from high-confidence prior knowledge for accurate estimation of transcription factor activities. *Nucleic acids research* (2023) 51:10934–10949. doi: 10.1093/NAR/GKAD841

18. Badia-I-Mompel P, Vélez Santiago J, Braunger J, Geiss C, Dimitrov D, Müller-Dott S, Taus P, Dugourd A, Holland CH, Ramirez Flores RO, et al. decoupleR: ensemble of computational methods to infer biological activities from omics data. *Bioinformatics Advances* (2022) 2: doi: 10.1093/BIOADV/VBAC016

19. Schubert M, Klinger B, Klünemann M, Sieber A, Uhlitz F, Sauer S, Garnett MJ, Blüthgen N, Saez-Rodriguez J. Perturbation-response genes reveal signaling footprints in cancer gene expression. *Nature Communications 2017 9:1* (2018) 9:1–11. doi: 10.1038/s41467-017-02391-6

20. Gavai AK, Supandi F, Hettling H, Murrell P, Leunissen JAM, Van Beek JHGM. Using Bioconductor Package BiGGR for Metabolic Flux Estimation Based on Gene Expression Changes in Brain. *PLOS ONE* (2015) 10:e0119016. doi: 10.1371/JOURNAL.PONE.0119016

21. King ZA, Lu J, Dräger A, Miller P, Federowicz S, Lerman JA, Ebrahim A, Palsson BO, Lewis NE. BiGG Models: A platform for integrating, standardizing and sharing genome-scale models. *Nucleic Acids Research* (2016) 44:D515–D522. doi: 10.1093/NAR/GKV1049

22. Gohel D. flextable: Functions for Tabular Reporting. (2022) https://cran.r-project.org/web/packages/flextable/index.html

23. Wilke CO. *Fundamentals of Data Visualization: A Primer on Making Informative and Compelling Figures*. 1st ed. Sebastopol: O’Reilly Media (2019).

24. Allaire J, Xie Y, McPherson J, Luraschi J, Ushey K, Atkins A, Wickham H, Cheng J. rmarkdown: Dynamic Documents for R. (2022) https://cran.r-project.org/web/packages/rmarkdown/index.html

25. Xie Y. *Bookdown: Authoring books and technical documents with R Markdown*. (2016). doi: 10.1201/9781315204963

26. Xie Y. knitr: A General-Purpose Package for Dynamic Report Generation in R. (2022) https://cran.r-project.org/web/packages/knitr/index.html

27. Liu J, Lichtenberg T, Hoadley KA, Poisson LM, Lazar AJ, Cherniack AD, Kovatich AJ, Benz CC, Levine DA, Lee AV, et al. An Integrated TCGA Pan-Cancer Clinical Data Resource to Drive High-Quality Survival Outcome Analytics. *Cell* (2018) 173:400–416.e11. doi: 10.1016/J.CELL.2018.02.052

28. Lewin J, Soltan Ghoraie L, Bedard PL, Hamilton RJ, Chung P, Moore M, Jewett MAS, Anson-Cartwright L, Virtanen C, Winegarden N, et al. Gene expression signatures prognostic for relapse in stage I testicular germ cell tumours. *BJU international* (2018) 122:814–822. doi: 10.1111/BJU.14372

29. Sturm G, Finotello F, List M. Immunedeconv: An R Package for Unified Access to Computational Methods for Estimating Immune Cell Fractions from Bulk RNA-Sequencing Data. *Methods in molecular biology (Clifton, NJ)* (2020) 2120:223–232. doi: 10.1007/978-1-0716-0327-7_16

30. Becht E, Giraldo NA, Lacroix L, Buttard B, Elarouci N, Petitprez F, Selves J, Laurent-Puig P, Sautès-Fridman C, Fridman WH, et al. Estimating the population abundance of tissue-infiltrating immune and stromal cell populations using gene expression. *Genome Biology* (2016) 17:218. doi: 10.1186/s13059-016-1070-5

31. Aran D, Hu Z, Butte AJ. xCell: Digitally portraying the tissue cellular heterogeneity landscape. *Genome Biology* (2017) 18:220. doi: 10.1186/s13059-017-1349-1

32. Finotello F, Mayer C, Plattner C, Laschober G, Rieder Di, Hackl H, Krogsdam A, Loncova Z, Posch W, Wilflingseder D, et al. Molecular and pharmacological modulators of the tumor immune contexture revealed by deconvolution of RNA-seq data. *Genome Medicine* (2019) 11:34. doi: 10.1186/s13073-019-0638-6

33. Maeser D, Gruener RF, Huang RS. oncoPredict: an R package for predicting in vivo or cancer patient drug response and biomarkers from cell line screening data. *Briefings in Bioinformatics* (2021) 22:1–7. doi: 10.1093/BIB/BBAB260

34. Geeleher P, Cox N, Stephanie Huang R. pRRophetic: An R Package for Prediction of Clinical Chemotherapeutic Response from Tumor Gene Expression Levels. *PLOS ONE* (2014) 9:e107468. doi: 10.1371/JOURNAL.PONE.0107468

35. Yang W, Soares J, Greninger P, Edelman EJ, Lightfoot H, Forbes S, Bindal N, Beare D, Smith JA, Thompson IR, et al. Genomics of Drug Sensitivity in Cancer (GDSC): a resource for therapeutic biomarker discovery in cancer cells. *Nucleic Acids Research* (2013) 41:D955–D961. doi: 10.1093/NAR/GKS1111

36. Seashore-Ludlow B, Rees MG, Cheah JH, Coko M, Price EV, Coletti ME, Jones V, Bodycombe NE, Soule CK, Gould J, et al. Harnessing Connectivity in a Large-Scale Small-Molecule Sensitivity Dataset. *Cancer discovery* (2015) 5:1210–1223. doi: 10.1158/2159-8290.CD-15-0235

37. Hänzelmann S, Castelo R, Guinney J. GSVA: Gene set variation analysis for microarray and RNA-Seq data. *BMC Bioinformatics* (2013) 14:7. doi: 10.1186/1471-2105-14-7

38. Leek JT, Johnson WE, Parker HS, Jaffe AE, Storey JD. The sva package for removing batch effects and other unwanted variation in high-throughput experiments. *Bioinformatics* (2012) 28:882. doi: 10.1093/BIOINFORMATICS/BTS034

39. Wang C, Gu Y, Zhang K, Xie K, Zhu M, Dai N, Jiang Y, Guo X, Liu M, Dai J, et al. Systematic identification of genes with a cancer-testis expression pattern in 19 cancer types. *Nature Communications 2016 7:1* (2016) 7:1–12. doi: 10.1038/ncomms10499

40. CTpedia. CTpedia. (2024) http://www.cta.lncc.br/ [Accessed June 12, 2024]

41. Li Z, Li T, Yates ME, Wu Y, Ferber A, Chen L, Brown DD, Carroll JS, Sikora MJ, Tseng GC, et al. The EstroGene Database Reveals Diverse Temporal, Context-Dependent, and Bidirectional Estrogen Receptor Regulomes in Breast Cancer. *Cancer Research* (2023) 83:2656–2674. doi: 10.1158/0008-5472.CAN-23-0539/727161/AM/THE-ESTROGENE-DATABASE-REVEALS-DIVERSE-TEMPORAL

42. Massie CE, Lynch A, Ramos-Montoya A, Boren J, Stark R, Fazli L, Warren A, Scott H, Madhu B, Sharma N, et al. The androgen receptor fuels prostate cancer by regulating central metabolism and biosynthesis. *The EMBO Journal* (2011) 30:2719. doi: 10.1038/EMBOJ.2011.158

43. Naba A, Clauser KR, Hoersch S, Liu H, Carr SA, Hynes RO. The matrisome: In silico definition and in vivo characterization by proteomics of normal and tumor extracellular matrices. *Molecular and Cellular Proteomics* (2012) 11:M111.014647. doi: 10.1074/mcp.M111.014647

44. Petrov PB, Considine JM, Izzi V, Naba A. Matrisome AnalyzeR – a suite of tools to annotate and quantify ECM molecules in big datasets across organisms. *Journal of Cell Science* (2023) 136: doi: 10.1242/JCS.261255/325836/AM/MATRISOME-ANALYZER-A-SUITE-OF-TOOLS-TO-ANNOTATE

45. Field AP. Discovering statistics using IBM SPSS Statistics: and sex and drugs and rock ‘n’ roll, 4th edition. *Choice Reviews Online* (2013) 50:xviii, 908, xxxvi. http://www.uk.sagepub.com/field4e/default.htm

46. Cohen J. Statistical Power Analysis for the Behavioral Sciences. *Statistical Power Analysis for the Behavioral Sciences* (2013) doi: 10.4324/9780203771587

47. Funder DC, Ozer DJ. Evaluating Effect Size in Psychological Research: Sense and Nonsense. *Advances in Methods and Practices in Psychological Science* (2019) 2:156–168. doi: 10.1177/2515245919847202

48. Benjamini Y, Hochberg Y. Controlling the False Discovery Rate: A Practical and Powerful Approach to Multiple Testing. *Journal of the Royal Statistical Society: Series B (Methodological)* (1995) 57:289–300. doi: 10.1111/j.2517-6161.1995.tb02031.x

49. Rousseeuw PJ. Silhouettes: A graphical aid to the interpretation and validation of cluster analysis. *Journal of Computational and Applied Mathematics* (1987) 20:53–65. doi: 10.1016/0377-0427(87)90125-7

50. Venna J, Kaski S. Neighborhood preservation in nonlinear projection methods: An experimental study. *Lecture Notes in Computer Science (including subseries Lecture Notes in Artificial Intelligence and Lecture Notes in Bioinformatics)* (2001) 2130:485–491. doi: 10.1007/3-540-44668-0_68

51. Simon N, Friedman J, Hastie T, Tibshirani R. Regularization Paths for Cox's Proportional Hazards Model via Coordinate Descent. *Journal of Statistical Software* (2011) 39:1–13. doi: 10.18637/JSS.V039.I05

52. Harrell FE, Lee KL, Mark DB. Multivariable prognostic models: Issues in developing models, evaluating assumptions and adequacy, and measuring and reducing errors. *Statistics in Medicine* (1996) 15:361–387. doi: 10.1002/(SICI)1097-0258(19960229)15:4<361::AID-SIM168>3.0.CO;2-4

53. Graf E, Schmoor C, Sauerbrei W, Schumacher M. Assessment and comparison of prognostic classification schemes for survival data. *Statistics in Medicine* (1999) 18:2529–2545. doi: 10.1002/(sici)1097-0258(19990915/30)18:17/18<2529::aid-sim274>3.0.co;2-5

54. Newman MEJ, Girvan M. Finding and evaluating community structure in networks. *Physical Review E* (2004) 69:026113. doi: 10.1103/PhysRevE.69.026113

55. Yu G. Gene Ontology Semantic Similarity Analysis Using GOSemSim. *Methods in Molecular Biology* (2020) 2117:207–215. doi: 10.1007/978-1-0716-0301-7_11

56. Yu G, Li F, Qin Y, Bo X, Wu Y, Wang S. GOSemSim: an R package for measuring semantic similarity among GO terms and gene products. *Bioinformatics* (2010) 26:976–978. doi: 10.1093/BIOINFORMATICS/BTQ064

57. Heidegger I, Frantzi M, Salcher S, Tymoszuk P, Martowicz A, Gomez-Gomez E, Blanca A, Lendinez Cano G, Latosinska A, Mischak H, et al. Prediction of Clinically Significant Prostate Cancer by a Specific Collagen-related Transcriptome, Proteome, and Urinome Signature. *European urology oncology* (2024) doi: 10.1016/J.EUO.2024.05.014

58. Pichler R, Siska PJ, Tymoszuk P, Martowicz A, Untergasser G, Mayr R, Weber F, Seeber A, Kocher F, Barth DA, et al. A chemokine network of T cell exhaustion and metabolic reprogramming in renal cell carcinoma. *Frontiers in Immunology* (2023) 14:1208. doi: 10.3389/FIMMU.2023.1095195/BIBTEX
